# Structure–activity relationship of ^18^F-labeled PD-L1-targeting small molecule ligands: impact of radiolabeling strategy on affinity and in vivo performance

**DOI:** 10.1186/s41181-025-00359-2

**Published:** 2025-07-01

**Authors:** Fabian Krutzek, Cornelius K. Donat, Sven Stadlbauer

**Affiliations:** 1 Helmholtz-Zentrum Dresden-Rossendorf, Institute of Radiopharmaceutical Cancer Research, Bautzner Landstraße 400, 01328 Dresden, Germany; 2 School of Science, Faculty of Chemistry and Food Chemistry, Technical University Dresden, 01069 Dresden, Germany; 3 Chemical Biology Program, Memorial Sloan Kettering Cancer Center, 1275 York Avenue, New York, NY 10065 USA

**Keywords:** PD-L1, Radioligands, Positron emission tomography, Small molecules, Fluorine-18, Chelators

## Abstract

**Background:**

Immune checkpoint inhibitor therapy addressing the PD-1/PD-L1 axis is a promising approach in cancer treatment. A clinically suitable radiotracer would allow molecular imaging of the temporospatial changes in tumor PD-L1 expression. This could enable the clinicians to select eligible patients for checkpoint therapy and monitor therapeutic efficacy.

**Results:**

Four biphenyl-based small-molecule PD-L1 ligands were synthesized using a convergent synthetic route, with a linear sequence of up to eleven steps. Two candidates were covalently labeled with ^18^F via either an azido glycosyl or PEG2 moiety, while the other two were modified with a RESCA chelator for Al[^18^F]F^2+^-labeling. The lipophilicity was assessed through determination of log *D*_7.4_ values. In vitro binding affinities (inhibition constant, *K*_i_) toward PD-L1 were determined in competition with one of our previously published biphenyl-based small-molecule (*K*_D_ =  ~ 21 nM). Compared to this compound, both covalently labeled ^18^F-ligands exhibited decreased water solubility (log *D*_7.4_ ~ − 2.5 and − 2.7), along with a markedly reduced (*K*_i_ = 200‒500 nM) affinity. This was in line with in vivo small animal PET, where both compounds were characterized by a negligible tumor uptake, lack of contrast between target-positive/negative tumors and exclusively unfavorable hepatobiliary excretion. Similar results were observed for the chelator-modified ligands with slightly increased hydrophilicity (log *D*_7.4_ ~ − 2.8 and − 2.9), showing a binding affinity of 150 nM for one compound, while binding was lost completely for the other. Again, a poor in vivo performance was observed, characterized by hepatobiliary clearance and lack of specific tumor uptake in the PD-L1 positive tumor.

**Conclusion:**

Four biphenyl-based, ^18^F-labeled PD-L1 radioligands were developed using prosthetic groups (azido glycosyl or PEG2) for covalent fluorination and Al[^18^F]F^2^⁺-complexation with the RESCA chelator. Despite limited in vitro and in vivo performance, these fluorination approaches offer a foundation for developing improved PD-L1 radioligands after increasing the hydrophilicity and the spacing between the radiolabel and binding motif.

**Supplementary Information:**

The online version contains supplementary material available at 10.1186/s41181-025-00359-2.

## Background

The tumor microenvironment (TME) operates as a dynamic and immunological network, involving a variety of cellular actors. Among others, T- and B-cells, natural killer (NK cells) and endothelial cells interact in the TME with both the extracellular matrix and cancer cells (Anderson and Simon 2020; Balkwill et al. 2012). Moreover, the changes in comparison to homeostatic tissues often presents biomarkers, such as receptors and enzymes, which can be targeted using molecular imaging. Potentially, this allows an early diagnosis of neoplastic changes, or a staging, thus aiding treatment decisions and therapeutic monitoring. The programmed cell death ligand 1, PD-L1 (CD274), has been shown to be an attractive target for novel immunotherapies and has consequently gained considerable attention in recent years. Its interaction with PD-1 (CD279) forms an immune checkpoint which is an important regulator of the immune response under homeostatic conditions, preventing autoimmunity effects (Han et al. 2020; Santini and Hellmann 2018). Throughout cancer progression, overexpression of the immune checkpoint target, such as PD-L1, enables tumor growth via evasion of the local immune response in the TME. Consequently, blockade of PD-1 or PD-L1 with inhibitors represents a promising therapeutic strategy, as reactivation of the local immune response in the TME leads to the identification and targeting of malignant tissue, e.g. via T-cells (Budimir et al. 2022). Antibodies constitute the dominant structural class for this type of treatment. However, antibody production and therapies cause substantial medical care costs and were found to be associated with several adverse effects, for instance caused by immunogenicity (Wu et al. 2022). Most importantly, PD-L1 antibody monotherapies resulted in low response rates of about 30% (Farid 2007; Guardascione and Toffoli 2020; Huang et al. 2020).

At present, patient stratification and treatment decisions rely on biopsies and immunohistochemical staining. For the patient, this causes distress when performed regularly (Munari et al. 2017). Because PD-L1 expression in the tumor can be characterized by a temporospatial heterogeneity, immunostaining of small bioptic samples can result in either a false-positive or -negative diagnosis (Yi et al. 2018). Conversely, noninvasive molecular imaging approaches, such as positron emission tomography (PET) and single-photon emission computed tomography (SPECT), offer the possibility to accurately and quantitatively assess PD-L1 expression in the whole tumor and over time (Rudin and Weissleder 2003). This can eventually support patient stratification and thus increase success rates of checkpoint inhibitor therapies. In addition, PD-L1 imaging could allow assessing the treatment response of bispecific T-cell engagers (BiTEs), targeting e.g. CD3 and PD-L1 (Liu et al. 2021) or CD3 and DLL3 (Tang and Kang 2023). In the latter case, Tarlatamab was recently approved for the treatment of small cell lung cancer (SCLC) by the FDA resulting in an upregulation of the PD-L1 expression (Rudin et al. 2023). A kinetically fast-acting small molecule PD-L1 radiotracer would allow to monitor this treatment-specific response.

To date, a variety of radiotracers targeting PD-L1 or PD-1 have been developed (Krutzek et al. 2022), including large and midsized constructs, such as antibodies (Heskamp et al. 2015; Jagoda et al. 2019; Kikuchi et al. 2017; Li et al. 2018), nanobodies (Bridoux et al. 2020; Broos et al. 2017), and adnectines (Donnelly et al. 2018; Stutvoet et al. 2020). Additionally, smaller structures like peptides (Kuan et al. 2019; Zhang et al. 2023) and small molecules (Bamminger et al. 2024; Hu et al. 2023; Huang et al. 2023; Lu et al. 2024; Lv et al. 2021; Maier et al. 2023; Miao et al. 2020; Xu et al. 2023; Yang et al. 2024; Zhu et al. 2023) were described. The use of radiolabeled clinical antibodies has yielded promising results, mainly through their high affinity and thorough prior clinical trials. However, they exhibit long circulation times (Pollack et al. 2018), necessitating the use of long-lived radioisotopes and thereby increasing the patient's radiation burden. Furthermore, accumulation in nonmalignant tissues, e.g. lymph nodes, are commonly found (Bensch et al. 2018; Smit et al. 2022). In contrast, peptides and small molecules offer favorable properties as imaging agents due to their enhanced tissue and tumor penetration, along with rapid clearance (Adams et al. 2015). This combination can result in remarkable imaging contrast within a short time. The cyclic peptide WL12 is such an example, showing excellent binding affinity and specificity, along with suitable pharmacokinetics in vivo (Chatterjee et al. 2017; De Silva et al. 2018; Lesniak et al. 2019). Hence, this peptide has recently been advanced to clinical trials, marking a significant step forward in this field (Wu et al. 2024; Zhou et al. 2022, 2025). In contrast, small molecule-based PD-L1 radiotracers yielded fewer promising outcomes so far, mainly caused by high non-specific binding, relatively low in vivo tumor uptake, and primarily hepatobiliary clearance (Bamminger et al. 2024; Hu et al. 2023; Huang et al. 2023; Lu et al. 2024; Lv et al. 2021; Maier et al. 2022; Miao et al. 2020; Xu et al. 2023; Yang et al. 2024; Zhu et al. 2023). While not the single cause of their shortcoming, high lipophilicity of the inhibitor lead structures are certainly affecting the in vivo performance.

**Fig. 1:**
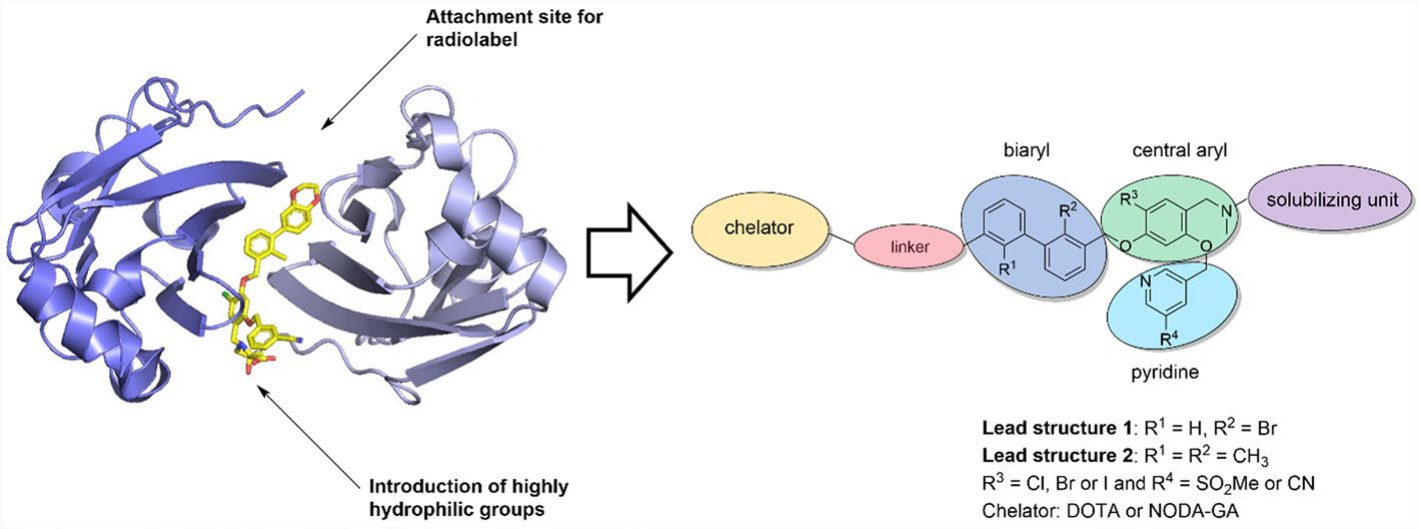
Left: Cocrystal structure of dimeric PD-L1 with the small molecule inhibitor BMS1166 (PDB code: 6R3K) and identified attachment sites for radiolabel and hydrophilic groups; Right: generalized structure of our PD-L1 small molecule-based radioligands, with relevant modification sites for solubilizer, linker and chelator (Stadlbauer et al. 2023)

To address this, we have developed a number of small molecule-based PET ligands for the imaging of PD-L1 overexpressing tumors, derived from biphenyl small molecule immune checkpoint inhibitors reported in the patent literature. In order to overcome the high lipophilicity of the parent molecules, highly hydrophilic sulfonic and/or phosphonic acid groups were introduced in the so-called solubilizing unit (Fig. 1) of the molecule (Krutzek et al. 2024, 2023a, 2023b, 2023c). The ^64^Cu-labeled radiotracers exhibited favorable low log *D*_7.4_ values between ‒2.7 and ‒4.3. In vitro binding affinities (expressed as dissociation constants *K*_D_) were between 1.8 and 608 nM and in vivo PET imaging revealed vastly different pharmacokinetic profiles, depending on the number, pattern and type of water-solubilizing groups. Among these previously reported ^64^Cu-radioligands from five different compound series, the radiotracer containing three sulfonate groups exhibited the most promising properties (Krutzek et al. 2023b). For that radiotracer, the real-time radioligand binding showed an affinity of 21 nM (expressed as *K*_D_), but more importantly, a vastly superior pharmacokinetic in vivo profile. However, binding affinity and tumor uptake of these small molecule PET tracers could still be improved, while retaining the fast pharmacokinetics including the desired renal clearance. For that reason, we aimed to replace the bulky chelator with fluorine-18 as radiolabel. On the one hand, fluorine-18 is the clinical PET radionuclide of choice due to its commercial availability, balanced half-life and its favorable imaging properties (Vallabhajosula 2009). On the other hand, its smaller size and the possibility to attach fluorine-18 covalently to the targeting molecule usually affects binding affinity to a lesser extent. Furthermore, it may also prove beneficial to other pharmacokinetic properties of the radioligands, e.g. resulting in shorter circulation times, better tissue penetration and faster/higher accumulation in target tissue.

## Results

### Design of PD-L1 radioligands

Previously, we reported several candidates with lowered lipophilicity through hydrophilic chelators and linkers, a successful strategy for the clinically applied PSMA and FAPI small molecule radioligands (Benesova et al. 2016; Lindner et al. 2021) (Fig. 1).

**Fig. 2:**
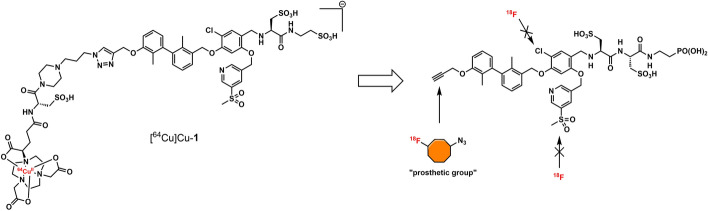
Lead structure [^64^Cu]Cu-**1** and derived structure of ^18^F ligands with potential labeling sites for ^18^F within the binding motif

**Fig. 3:**
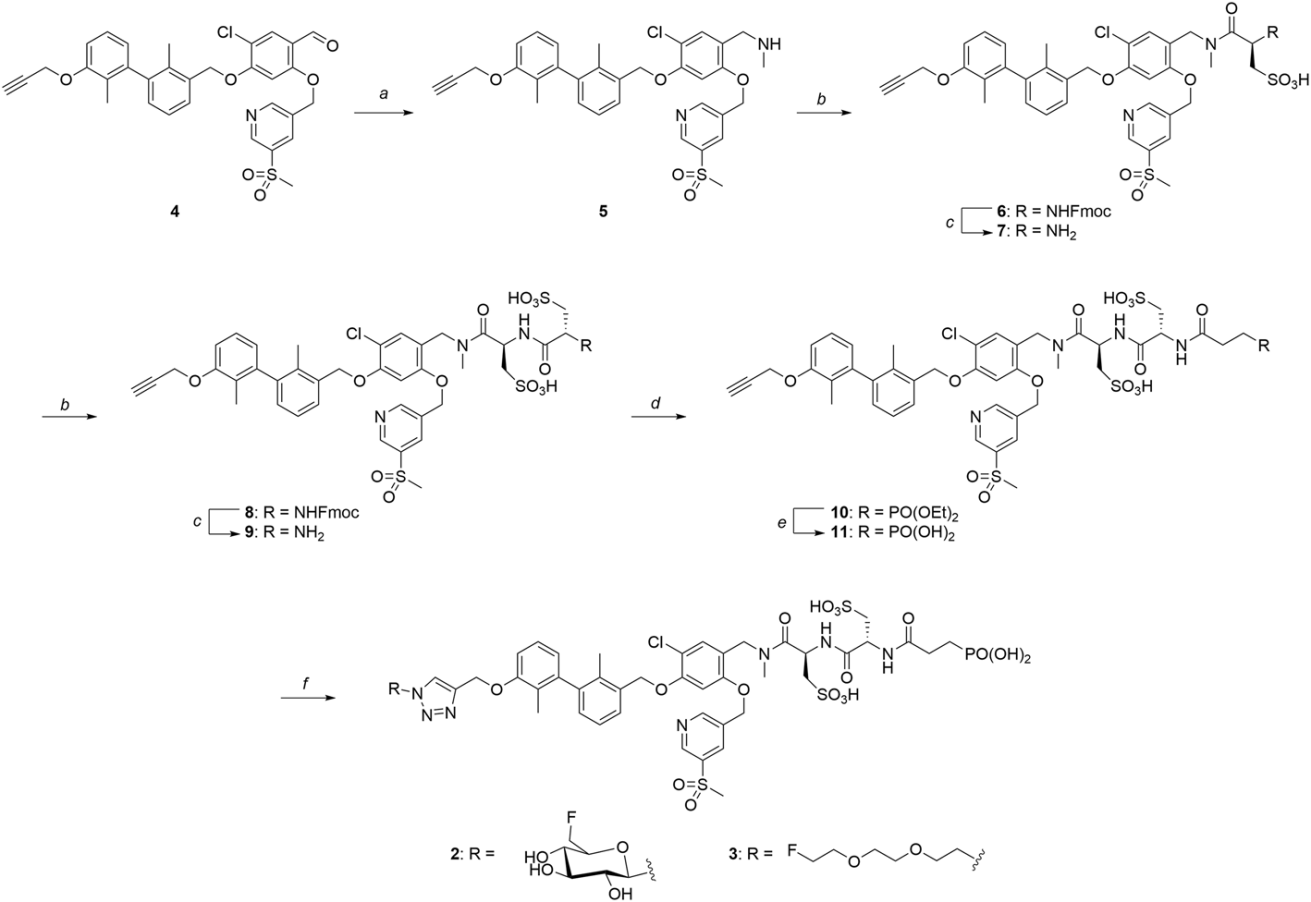
Synthesis scheme towards ^19^F-compounds **2** and **3**: **a** methylamine (33% in EtOH), NaBH_3_CN, abs. THF/MeOH (1:1), 0–70 °C, 16 h, 84%; **b** Fmoc-l-cysteic acid, HATU, HOBt, DIPEA, DMF, r.t., 3 h; **c** piperidine (20% in DMF), r.t., 1 h, 64% for **7** and 61% for **9** (over two steps, respectively); **d** diethyl ciliatine, HATU, DIPEA, DMF, r.t., 3 h, 75%; **e** TMSBr, abs. DMF, r.t., 40 h, 48%; **f** 2-azido-6-fluoroglucose or 1-azido-2-(2-(2-fluoroethoxy)ethoxy)ethane, CuSO_4_, sodium ascorbate, THPTA, H_2_O/^*t*^BuOH (1:1), r.t., 3 h, 90% for **1** and 84% for **2**

While this resulted in much lower log *D*_7.4_ values (− 2.1 to − 4.3), affinity to PD-L1 differed markedly between 1.8 and 608 nM and vastly different pharmacokinetic profiles were observed in vivo using PET, depending on the modifications. Compound [^64^Cu]Cu-**1** (Fig. 2) with three sulfonate groups exhibited the most promising properties (Krutzek et al. 2023b) and served as lead for the ^18^F-derivatives. This was driven by fluorine-18’s role as clinical PET radionuclide of choice (Vallabhajosula 2009) and a lower molecular weight, allowed via its covalent attachment. The latter was anticipated to positively affect binding and pharmacokinetics. Replacing chlorine in the central aryl unit or SO_2_Me in the pyridine moiety with a fluorine atom would be possible in general (Fig. 2). However, inhibitors bearing a fluorine atom at either of these positions were found to exhibit reduced binding affinities (Aktoudianakis et al. 2018). Additionally, introduction of fluorine-18 via direct nucleophilic substitution is hampered due to the presence of strongly acidic groups, which would generate [^18^F]HF and thus ultimately prevent the radiofluorination of the molecule. Therefore, a prosthetic group approach seemed best suited, and the resulting radiofluorinated prosthetic group will be reacted with the alkyne moiety of the ligand structure via Cu-catalyzed click chemistry. Ideally, the prosthetic group should contribute to the water solubility of the radioligand, which makes the azido sugar labeling precursors reported by Prante et al*.* (Maschauer et al. 2010, 2014; Maschauer and Prante 2009) and PEG2-labeling (Böhmer et al. 2020) appealing for this purpose. This also comes with the advantage of increased hydrophilicity via the prosthetic group. Compared to the chelator-based radioligands, the glycoside or PEG2-substituted ^18^F-radioligands do not possess a hydrophilic linker with sulfonic or phosphonic acids. Neither the sugar nor the PEG2 moiety would be able to counteract this loss in water solubility. To compensate for the missing hydrophilic linker in the ^18^F-radioligands and to achieve the desired renal clearance, a third acid group in the form of phosphonate-bearing ciliatine was introduced in the solubilizing unit of the molecule. This resulted in structures **2** and **3** (Fig. 3).

### Synthesis

The synthesis toward **2** and **3** started from previously reported aldehyde building block **4** (Krutzek et al. 2023c), which underwent reductive amination, followed by repeated amide coupling with l-cysteic acid and Fmoc deprotection leading to amine **9** (Fig. 3). Then diethyl (2-aminoethyl) phosphonate was coupled and the free phosphonate groups liberated through McKenna reaction with TMSBr in DMF, yielding key building block **11**. With that alkyne in hands, the non-radioactive ^19^F-reference compounds **2** and **3** were synthesized through copper(I)-catalyzed azide–alkyne cycloaddition (CuAAC), employing either 2-azido-6-fluoroglucose (Böhmer et al. 2020; Maschauer et al. 2014) or commercially available 1-azido-2-(2-(2-fluoroethoxy)ethoxy)ethane. Corresponding NMR (S1-S34) and MS-Spectra (S48-S65) as well as HPLC chromatograms (S35-S47) are reported in the Supplementary Material.

### Radiochemistry

**Fig. 4:**
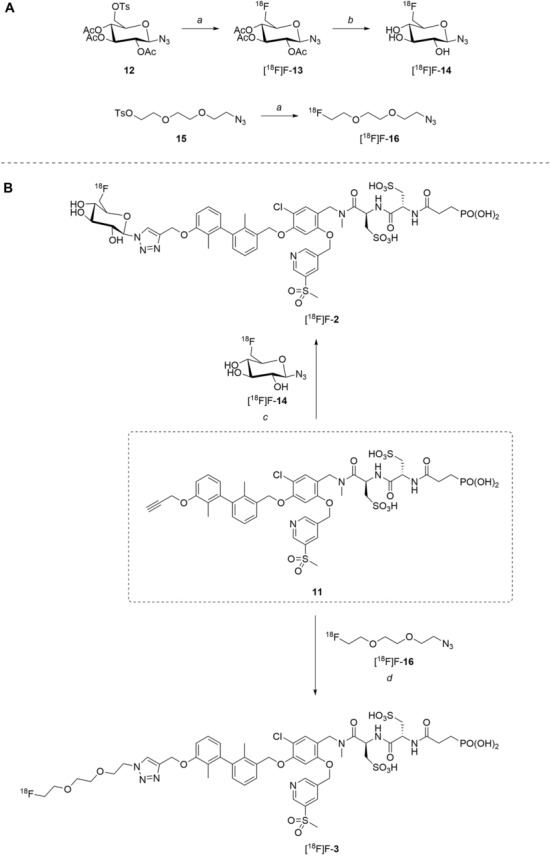
A) Radiolabeling procedures using prosthetic groups [^18^F]F-**14** and [^18^F]F-**16**: **a** **12** (10 mg) or **15** (3 mg), [K^+^  ⊂ Kryptofix®_222_][^18^F]F^−^ complex, dry MeCN, 90 °C, 10 min; **b** 120 mM NaOH, 70 °C, 5 min, RCY = 11–21% (n = 10); B) CuAAC reaction of prosthetic groups to PD-L1 binding motif **11**: **c** [^18^F]F-**14**, CuSO_4_, sodium ascorbate, THPTA, EtOH/H_2_O (1:1), 70 °C, 10 min, **d** [^18^F]F-**16**, CuSO_4_, sodium ascorbate, THPTA, ^*t*^BuOH/H_2_O (1:1), 70 °C, 10 min, RCY = 16–25% (n = 10)

For accessing the ^18^F-ligands, first the labeling precursors **12** and **15** were synthesized as previously reported (Maschauer et al. 2014). Starting from **12**, radiofluorination was performed according to the procedures established by Prante et al., followed by removal of acetyl protecting groups under basic conditions, yielding [^18^F]F-**14** (Fig. 4A). The PEG2 derivative [^18^F]F-**16** was available in a one-step S_N_2 reaction with [^18^F]F^−^. Employing CuAAC, both prosthetic groups were attached to alkyne **11** yielding [^18^F]F-**2** in radiochemical yields of 11–21% and [^18^F]F-**3** in 16–24% (n = 10, respectively; Fig. 4B, HPLC-chromatograms S66-S67).

With both ^18^F-ligands in hand, distribution coefficients (log *D*_7.4_) were determined using the shake flask method with *n*-octanol and PBS. The introduction of three hydrophilizing units led to log *D*_7.4_ values of − 2.54 ± 0.02 for [^18^F]F-**2** and − 2.68 ± 0.08 for [^18^F]F-**3**, which indicates moderate hydrophilicity. Subsequently, proteolytic stability was investigated (Supplementary Fig. S68 and S69), by incubating in human serum at 37 °C for up to 4 h. Before incubation (0 h) and 0.5, 1, 2 and 4 h after incubation, an aliquot was taken, and serum proteins were precipitated. Analysis of radiotracers was performed with radio-HPLC, showing the intact radiotracers (0 h) at 7.0 ([^18^F]F-**2**) and 7.7 min ([^18^F]F-**3**). At all time points, radiotracers remained intact up to 99%.

### In vitro studies

#### Competition binding

**Fig. 5:**
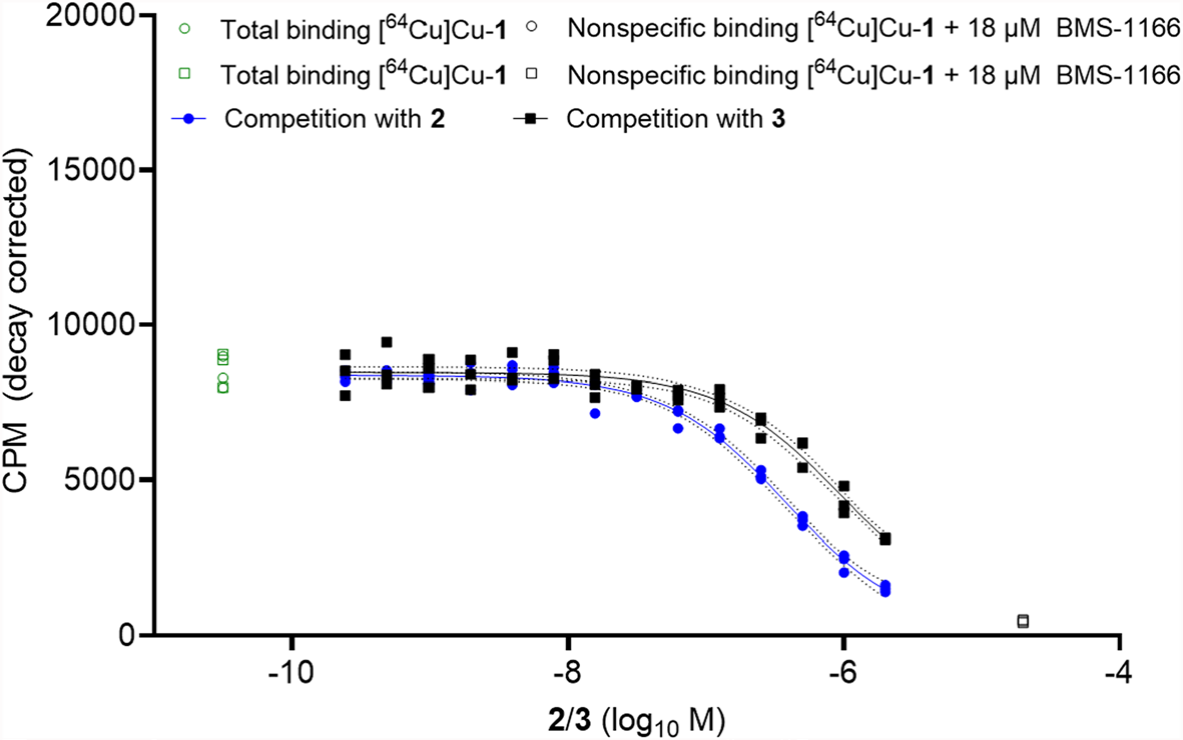
Inhibition of [^64^Cu]Cu-**1** binding to PC3 PD-L1 cells by compounds **2**/**3**. Data is derived from 14 dilutions (1 µM–120 pM) of the respective compound. Inhibitory constant (*K*_i_) along with 95% CI are provided (n = 2/compound, from triplicates)

Initial findings using real-time radioligand binding yielded minimal to low signal increase over time, complicating accurate fitting of the trace (Supplementary Fig. S72). This prompted us to utilize a competition assay (Fig. 5, Table 1). We employed our most promising compound so far, [^64^Cu]Cu-**1** with an affinity of ~ 21 nM. Over a wide concentration range (1 µM–120 pM), fluorinated compounds **2** and **3** were competing with [^64^Cu]Cu-**1** for binding to PC3 PD-L1 overexpressing cells. Unfortunately, the findings observed in real-time radioligand binding were confirmed. Both **2** and **3** showed a severely reduced ability to inhibit binding of [^64^Cu]Cu-**1** to PC3 PD-L1 cells. This resulted in substantially increased *K*_i_ for **2** and **3** with 189 and 475 nM, respectively. It must therefore be concluded that the particular structural modifications exerted a negative effect on binding capability.

**Table 1:** In vitro data (^1^ Inhibitory constant *K*_i_ from competition binding assay) for **2**/**3** against [^64^Cu]Cu-**1**, 95% confidence intervals and their respective log *D*_7.4_ values (determined for [^18^F]F-**2**/[^18^F]F-**3**)

Compound	*K*_i_ [nM]^1^	95% CI, [nM]	Log *D*_7.4_
[^18^F]F-**2**	189	157–226	− 2.54 ± 0.02
[^18^F]F-**3**	475	358–678	− 2.68 ± 0.08

### In vivo* binding pattern*

**Fig. 6:**
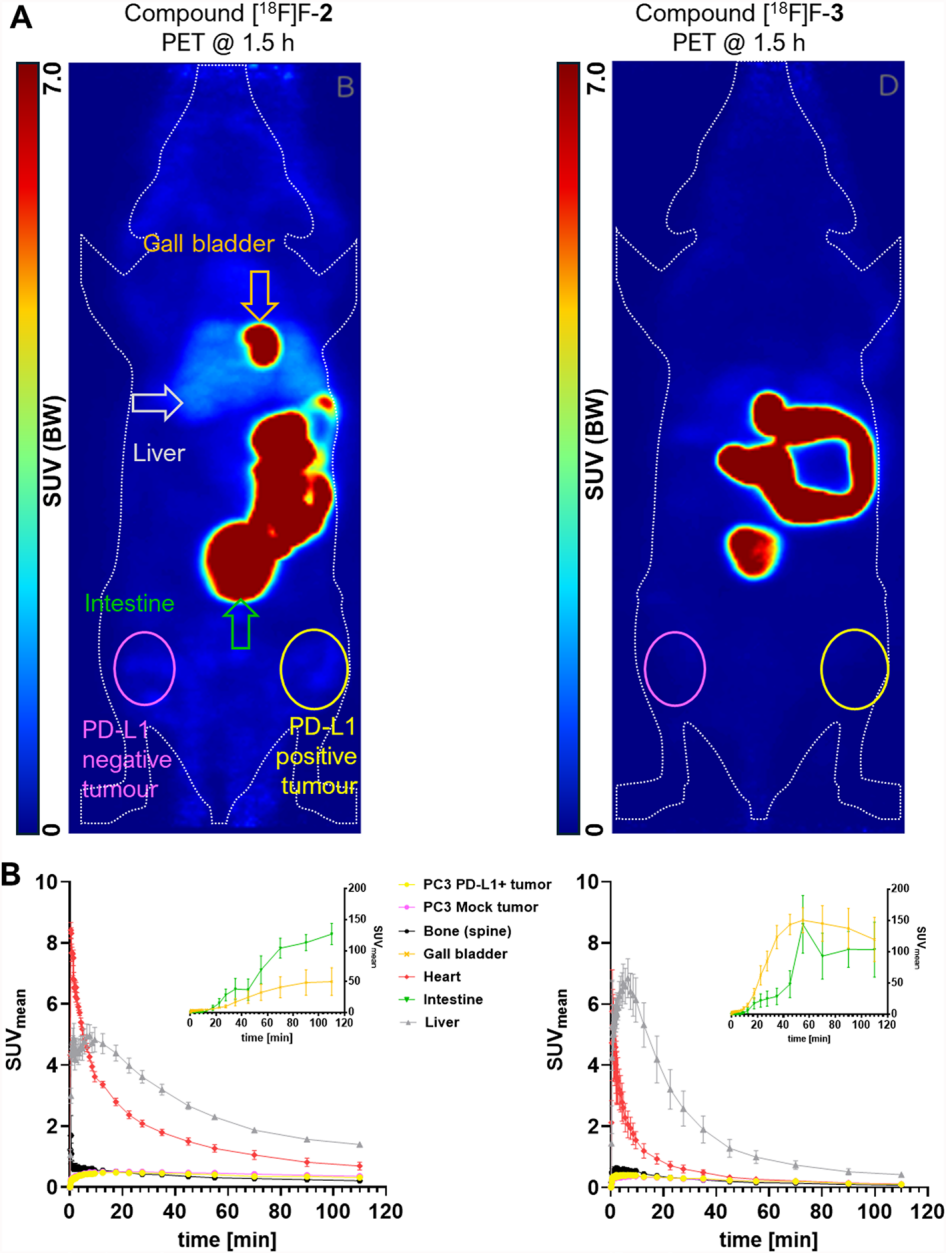
**A** Small animal PET images (as Maximum intensity projections) of [^18^F]F-**2** (left) and [^18^F]F-**3** (right) in NRMI nude mice (n = 4 for each compound) bearing target-positive (PC3 PD-L1, right thigh) and target-negative (PC3 mock, left thigh) tumors. Organs of interest (liver, gall bladder, intestine, heart and joints/bone) are highlighted here or in Fig. 8. **B** Time-activity curves of radioactivity distribution in selected volumes of interest for [^18^F]F-**2** (left) and [.^18^F]F-**3** (right)

Small animal PET added further proof to the observed low inhibitory constant/affinity of compounds [^18^F]F-**2** and [^18^F]F-**3**. The pharmacokinetic pattern (Fig. 6A/B) of radiolabeled [^18^F]F-**2** was characterized by a slightly longer circulation time than [^18^F]F-**3**, as indicated by activity in ascending aortae and heart (Fig. 6B, Table 2). Both radiotracer candidates were exclusively excreted via the hepatobiliary route. This was characterized by a very fast transition (within 10–15 min) from liver and gall bladder into the intestine (Fig. 6B). In the intestine, activity slowly decreased towards the end of the observation period (2 h p.i.), still remaining very high (SUV_mean_: 80–220). Owing to their covalent labeling, compounds [^18^F]F-**2** and [^18^F]F-**3** lacked a discernible bone uptake. Both radiotracer candidates also showed a marked absence of target-specific uptake. PC3 PD-L1 tumors were found to exhibit very low uptake ([^18^F]F-**2**; SUV_max_, 1–2 h p.i.: 0.66 (n = 4); [^18^F]F-**3**; SUV_max_, 1–2 h p.i.: 0.30 (n = 4)). More importantly, this uptake was always in the similar range as found for the PC3 mock tumor uptake ([^18^F]F-**2**; SUV_max_, 1–2 h p.i.: 0.77 (n = 4); [^18^F]F-**3**; SUV_max_, 1–2 h p.i.: 0.34 (n = 4)). Hence, all compounds were completely lacking contrast between target-positive and -negative tumor.

**Table 2:** In vivo tumor uptake (SUV_mean_) of [^18^F]F-**2**/[^18^F]F-**3** at 1–2 h p.i.; data is mean (n = 4) ± SD

Volume of interest	Compound [^18^F]F-**2**		Compound [^18^F]F-**3**	
	Uptake (SUV_mean_)		Uptake (SUV_mean_)	
	Mean	SD	Mean	SD
PC3 PD-L1 tumor	0.34	0.07	0.17	0.04
PC3 mock tumor	0.39	0.11	0.17	0.08
Liver	1.61	0.10	0.70	0.27
Gall bladder	46.45	39.79	150.54	52.49
Small intestine	114.55	19.37	105.54	54.83
Heart	0.86	0.27	0.20	0.08
Bone (spine)	0.25	0.06	0.13	0.03

### *Synthesis of chelator-modified PD-L1 ligands for Al[*^*18*^*F]F*^*2*+^*-labeling*

**Fig. 7:**
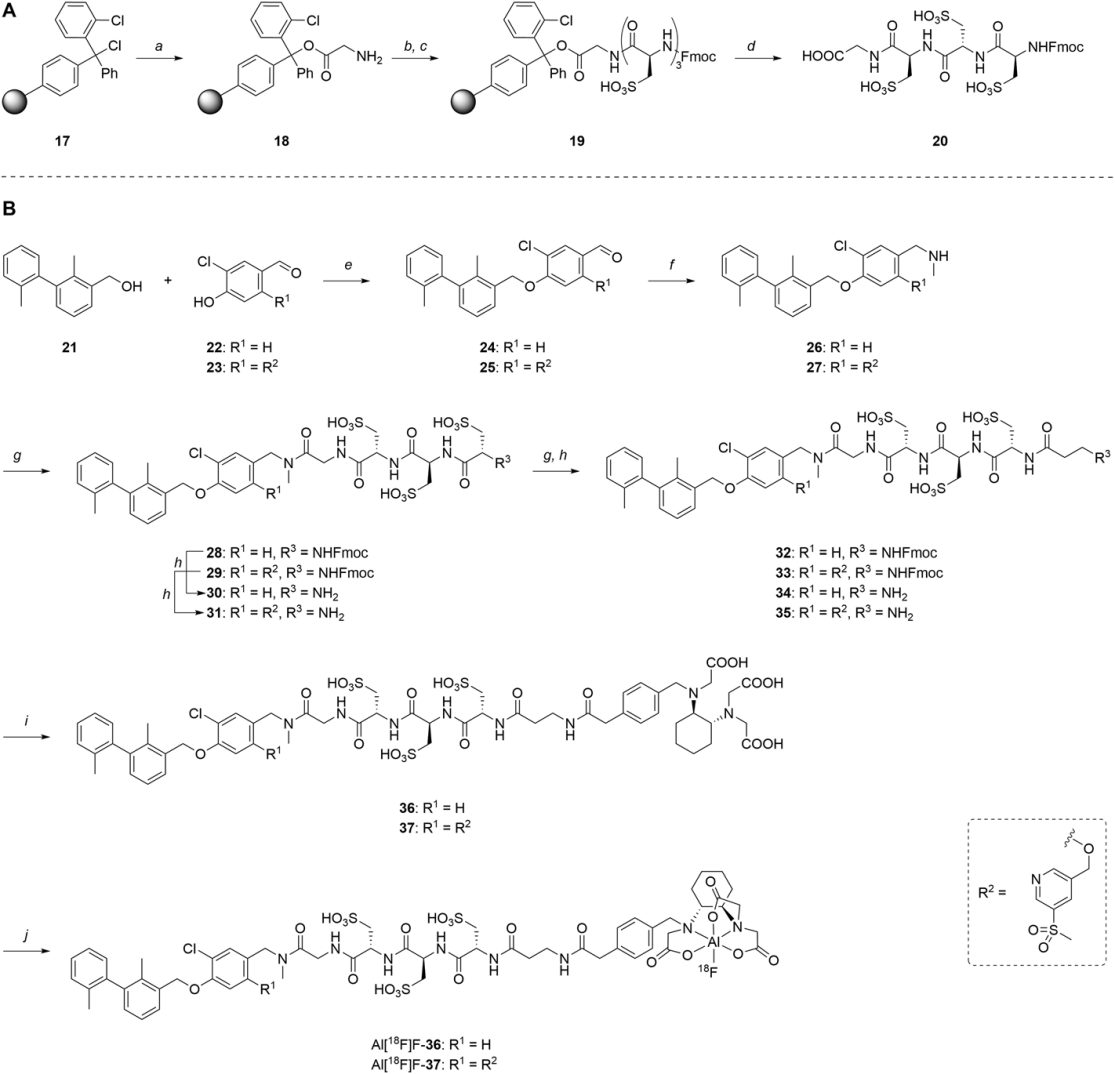
Synthesis scheme of chelator compounds **36** and **37** and their respective [^18^F]AlF^2^^+^-complex. **A** SPPS of linker structure **20**: a) Fmoc-Gly-OH, DIPEA, DMF, r.t., 2 h; b), Fmoc-cysteic acid, HATU, DIPEA, DMF, r.t., 1 h; c) piperidine (20% in DMF), r.t., 30 min; d) TFA (1% in DCM), r.t., 30 min. **B** e) DEAD, PPh_3_, abs. DMF, 0 °C to r.t., 16 h, 47% for **24** and 89% for **25**; f) methylamine (33% in EtOH), NaBH_3_CN, abs. THF/MeOH (1:1), 0 °C to 70 °C, 16 h, 84% for **26** and 73% for **27**; g) **20** or Fmoc-β-Ala-OH, HATU, HOBt, DIPEA, DMF, r.t., 3 h; h) piperidine (20% in DMF), r.t., 1 h, 45% for **30** and 65% for **31**, 40% for **34**, 74% for **35**; i) (+)-H_3_RESCA-TFP, DIPEA, abs. DMF, r.t., 2 h, 73% for **36**, 59% for **37**; j) [^18^F]F^−^, AlCl_3_, 1 M HEPES (adjusted to pH 4.5 with 1 M HCl)/EtOH (1:1), 50 °C, 15 min, RCYs ≥ 95%

We hypothesized that both the PEG2 and glycosyl moiety might interfere with binding to PD-L1 as compared to the ^64^Cu-ligands, which possess a linker and chelator in the same place. In contrast to our initial assumption, these results imply that a chelator-containing and therefore larger-sized molecule might not be a disadvantage and rather beneficial to binding. To test this hypothesis, aluminium-[^18^F]fluoride (Al[^18^F]F^2+^) was used to allow standard complexation chemistry of radiometals coupled with the favorable imaging properties of fluorine-18 (Archibald and Allott 2021). Complexation is possible under very mild conditions using the RESCA chelator (Cleeren et al. 2017). In addition, we wanted to study whether merging the linker-chelator construct with the hydrophilizing groups in the solubilizer unit would improve the binding affinities and tumor uptake. To this end, we designed two PD-L1 ligands containing a RESCA chelator for Al[^18^F]F^2+^-labeling (**36** and **37**, Fig. 7). Two different binding motifs were tested, the same binding motif as in **2** and **3**, and a binding motif lacking the pyridinyl ether moiety. As water-solubilizing units, three cysteic acids were used, which served at the same time as linker for conjugating the chelator. The synthesis of amines **26** and **27** is outlined in Fig. 7B. Mitsunobu reaction of biphenyl **21** with building blocks **22** or **23** provided aryl ethers **24** and **25** in 47 and 89% yield, respectively. These building blocks underwent subsequent reductive amination with methylamine, before conjugated to a tris(cysteic acid) peptide serving as a water-soluble linker. The peptide linker **20** was synthesized on solid support employing standard Fmoc peptide chemistry (Fig. 7A). After conjugation, a β-alanine was attached, which in turn allowed conjugation of the RESCA chelator using the corresponding TFP ester. Corresponding NMR (S21-S34) and MS-Spectra (S55-S63) as well as HPLC chromatograms (S41-S47) are reported in the Supplementary Material. The resulting labeling precursors **36** and **37** were then used for labeling with Al[^18^F]F^2+^ under mild reaction conditions (50 °C, 15 min), resulting in radiochemical yields (RCY) of ≥ 95%.

However, when hydrophilicity was assessed, only a marginal increase was found compared to [^18^F]F-**2** and [^18^F]F-**3**, with log *D*_7.4_ values of − 2.78 ± 0.03 for Al[^18^F]F-**36** and − 2.93 ± 0.13 for Al[^18^F]F-**37**.

### Proteolytic stability in serum

Subsequently, proteolytic stability was investigated (Supplementary Fig. S70 and S71), by incubating in human serum at 37 °C for up to 4 h. Before incubation (0 h) and 0.5, 1, 2 and 4 h after incubation, an aliquot was taken, and serum proteins were precipitated. Analysis of radiotracers was performed with radio-HPLC, showing the intact radiotracers (0 h) at 7.9 (Al[^18^F]F-**36**) and 8.1 min (Al[^18^F]F-**37**). After 4 h, both radiotracers remained intact up to 91% and 92%, respectively.

### In vitro studies

#### Competition binding

**Fig. 8:**
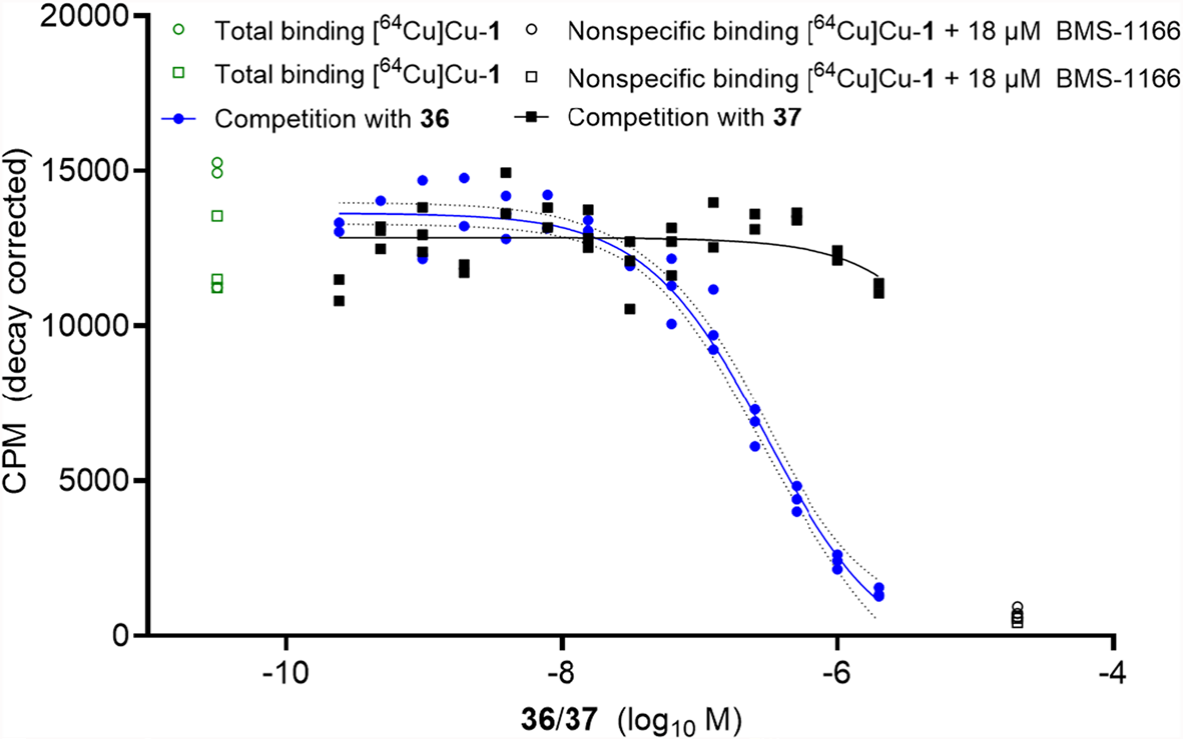
Inhibition of [^64^Cu]Cu-**1** binding to PC3 PD-L1 cells by compounds **36**/**37**. Data is derived from 14 dilutions (1 µM–120 pM) of the respective compound. Inhibitory constant (*K*_i_) along with 95% CI are provided (n = 2/compound, from triplicates)

Following an identical approach as performed for [^18^F]F-**2** and [^18^F]F-**3**, we also relied on competition binding for the Al[^18^F]F^2+^-labeled compounds Al[^18^F]F-**36** and Al[^18^F]F-**37** (Fig. 8, Table 3). When competing with [^64^Cu]Cu-**1** for binding to PC3 PD-L1 overexpressing cells, compound Al[^18^F]F-**36** yielded a *K*_i_ of 150 nM (Table 3). Affinity towards human PD-L1 of compound Al[^18^F]F-**37** was apparently lost completely, as demonstrated by lack of inhibitory potency (*K*_i_ > 1 µM) even in the highest concentration (1 µM). Again, despite the effort to compensate for changes introduced in the synthesis of [^18^F]F-**2** and [^18^F]F-**3**, no improvements in affinity were observed for Al[^18^F]F-**36** and rather a total loss for Al[^18^F]F-**37**.

**Table 3:** In vitro data (^1^ Inhibitory constant *K*_i_ from competition binding assay) for **36**/**37** against [^64^Cu]Cu-**1**, 95% confidence intervals and their respective log *D*_7.4_ values (determined for Al[^18^F]F-**36**/Al[^18^F]F-**37**). n/a: not applicable due to unstable fit

Compound	*K*_i_ [nM]^1^	95% CI, [nM]	Log *D*_7.4_
Al[^18^F]F-**36**	150	117–192	− 2.78 ± 0.03
Al[^18^F]F-**37**	> 1000	n/a	− 2.93 ± 0.13

### In-vivo studies

**Fig. 9:**
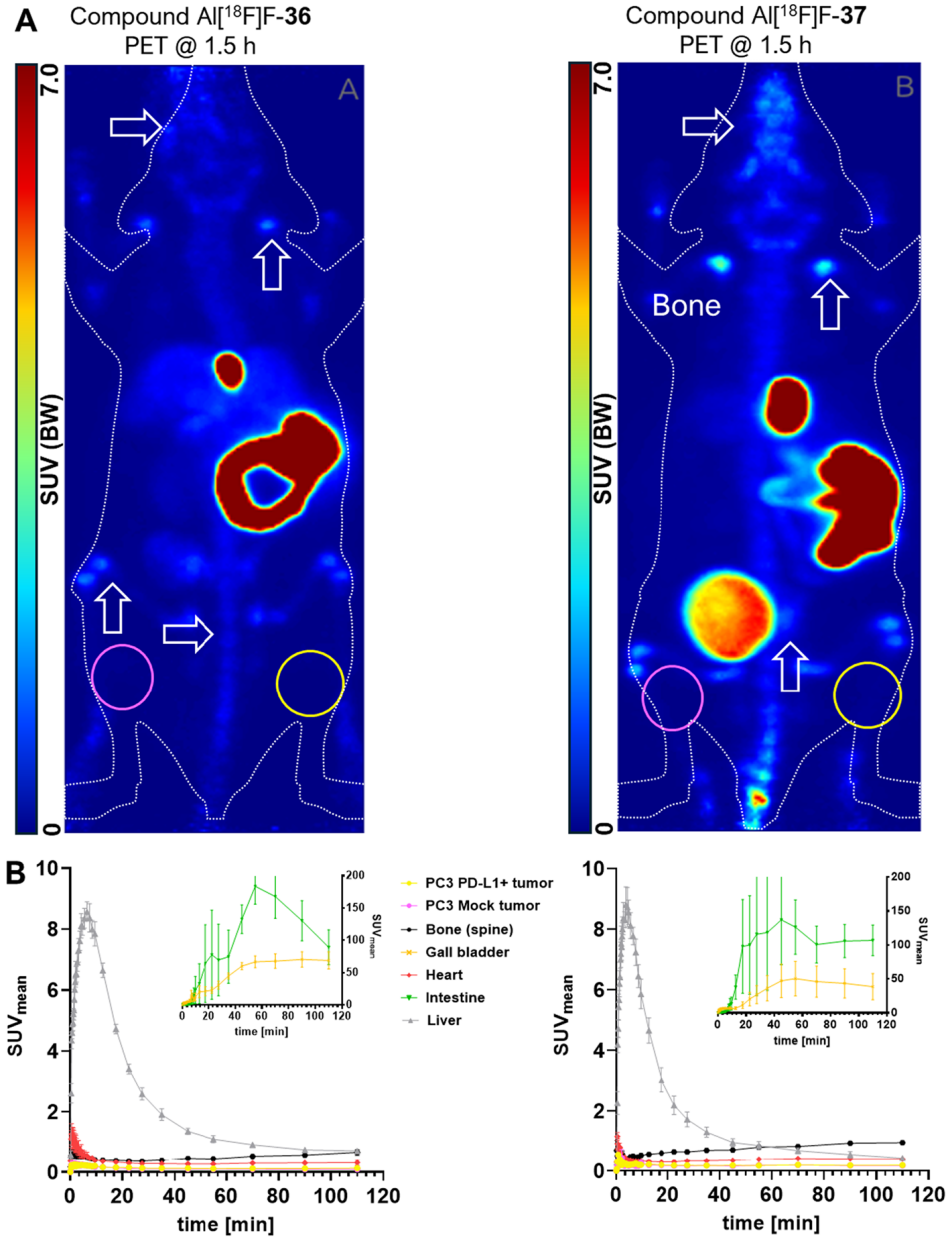
**A** Small animal PET images (as Maximum intensity projections) of Al[^18^F]F-**36** (left) and Al[^18^F]F-**37** (right) in NRMI nude mice (n = 3 for each compound) bearing target-positive (PC3 PD-L1, right thigh) and target-negative (PC3 mock, left thigh) tumors. Organs of interest are highlighted here or in Fig. 6. **B** Time-activity curves of radioactivity distribution in selected volumes of interest for Al[^18^F]F-36 (left) and Al[^18^F]F-**37** (right)

The pharmacokinetic pattern (Fig. 9A/B) of radiolabeled Al[^18^F]F-**36** and Al[^18^F]F-**37** was again found to be similar to the two covalently labeled candidates (**2** and **3**), despite the slight difference in affinity. Again, both candidates were exclusively excreted via the hepatobiliary route. Al[^18^F]F-**36** also showed a similarly fast transition from liver and gall bladder to the intestine (Fig. 9B). In contrast, small animal PET of Al[^18^F]F-**37** revealed a slightly slower intestinal transition. Interestingly, both Al[^18^F]F^2+^-labeled compounds were found to likely exhibit signs of defluorination. This was indicated by a moderate bone uptake that slowly increased over time (Fig. 9B; e.g. lower spine, SUV_mean_: 0.57/0.90).

Similarly, to the covalently labeled compounds, a total absence of target-specific uptake or contrast to the target-negative tumors was observed (Table 4). Again, a negligible uptake (Al[^18^F]F-**36**; SUV_max_, 1–2 h p.i.: 0.23 (n = 3); [Al[^18^F]F-**37**; SUV_max_, 1–2 h p.i.: 0.38 (n = 3)) was observed for PC3 PD-L1, in the same range as found for mock tumors (Al[^18^F]F-**36**; SUV_max_, 1–2 h p.i.: 0.19 (n = 3); Al[^18^F]F-**37**; SUV_max_, 1–2 h p.i.: 0.35 (n = 3)).

**Table 4:** In vivo tumor uptake (SUV_mean_) of Al[^18^F]F-**36**/Al[^18^F]F-**37** at 1–2 h p.i.; data is mean (n = 3) ± SD

Volume of interest	Compound Al[^18^F]F-**36**		Compound Al[^18^F]F-**37**	
	Uptake (SUV_mean_)		Uptake (SUV_mean_)	
	Mean	SD	Mean	SD
PC3 PD-L1 tumor	0.12	0.05	0.19	0.05
PC3 mock tumour	0.09	0.04	0.18	0.11
Liver	0.85	0.14	0.56	0.16
Gall bladder	69.52	20.31	42.74	36.70
Small intestine	149.11	65.77	104.36	42.66
Heart	0.30	0.15	0.41	0.04
Bone (spine)	0.57	0.12	0.90	0.04

## Discussion

Checkpoint inhibitor therapy provides a compelling curative approach for eligible cancer patients. However, not all patients benefit from the currently available antibodies (Yi et al. 2022; Yin et al. 2021), causing potential adverse side effects for the patient and high healthcare costs. Molecular imaging via SPECT and PET can provide the clinician with a better tool to ascertain the PD-L1 status of a tumor prior to a therapeutic decision, along with the chance to monitor the effectiveness of such a therapy. Furthermore, small molecule PD-L1 radiotracers offer additional avenues for treatment, such as receptor radionuclide therapy, given the compound’s suitability. In the past, a number of different potential radioligands for PD-L1 haven been developed, from anti- and nanobodies, to peptides, adnectines and small-molecules, all with different advantages and drawback. So far, only the cyclic peptide WL12 has shown favorable properties which translated to first in man and a clinical trial in non-small cell lung cancer (Zhou et al. 2021) (Zhou et al. 2025), followed by further human application (Wu et al. 2024).

In contrast, small-molecule radiotracers targeting PD-L1 were found to exhibit few promising results. This was often attributed to high non-specific and low specific binding, resulting in inadequate in vivo tumor uptake. Low hydrophilicity of the tracer candidates is one deciding factor contributing to the aforementioned problems and additionally often results in a primarily hepatobiliary clearance (Maier et al. 2022; Miao et al. 2020; Ważyńska et al. 2023; Xu et al. 2023). A number of recent studies attempted to improve this, with varying results (Bamminger et al. 2023; Hu et al. 2023; Xu et al. 2023; Yang et al. 2024; Zhu et al. 2023). In our previous attempts, we employed the biphenyl binding motif and probed various extensive modifications (Krutzek et al. 2024, 2023a, 2023b, 2023c). We succeeded in terms of hydrophilicity and achieved better in vivo pharmacokinetics, with renal clearance and higher tumor uptake (Krutzek et al. 2023b). However, none of the structural changes increased affinity and several modifications negatively impacted in vitro and/or in vivo performance (Krutzek et al. 2024, 2023a). Lowering molecular weight was one of the remaining strategies, however, difficult to achieve with a chelator-radiometal complex. Hence, by modifying [^64^Cu]Cu-**1**, we were able to achieve covalent or RESCA-chelator labeling with fluorine-18. This dual strategy allowed us to test two aspects: molecular weight and hydrophilicity. The covalently labeled compounds exhibited lower molecular weight, but with higher lipophilicity, while the RESCA-chelator had increased hydrophilicity but larger size. For the covalently labeled compounds, we hypothesized that the smaller ^18^F-labeled sugar/PEG2 unit may interfere less with PD-L1 dimer formation than the linker/chelator construct used previously, which could compensate for the loss of hydrophilicity. In contrast, the RESCA chelator could potentially compensate for the loss of hydrophilicity due to the lack of additional solubilizing groups. Due to the bulky tetra-peptide linker with the conjugated RESCA chelator, the construct was attached at the chloro-aryl position instead of the biaryl. As shown in Fig. 1 and supported by the literature (Guzik et al. 2017; Liang et al. 2023), this site lies in the solvent-exposed region of dimeric PD-L1, and should allow modifications without disrupting dimerization and therefore impairing binding affinity. However, both approaches were unsuccessful, as demonstrated by in vitro and in vivo data. Both synthetic strategies resulted neither in increased affinity towards PD-L1, but instead a drastically decreased binding capability in comparison to the NODA-GA compound [^64^Cu]Cu-**1**. In vitro, initial tests using real-time binding yielded very little signal for compound [^18^F]F-**2**/[^18^F]F-**3**, which prompted us to rather use a competition binding assay. There, three of the compounds were able to prevent binding of [^64^Cu]Cu-**1** to PD-L1 only in the high nanomolar range, while one compound lost its affinity completely. The reason for this pronounced loss of in vitro affinity remains to be elucidated. As the binding motif is very similar to [^64^Cu]Cu-**1**, the effect must be related to other aspects of the molecule. This could be caused by the number and positioning of solubilizing units in the linker structure. A larger space between the binding motif and the hydrophilizing units, e.g. by attaching a PEG-linker could be beneficial and increase binding affinity. This is supported by our previous findings, showing that number, type and position of solubilizing units, charge and chelator type (Krutzek et al. 2024, 2023a) can decisively influence binding capability. Furthermore, it demonstrates the peculiarity of the binding of biphenyl-based inhibitors towards PD-L1. This emphasizes that molecular size seems less important than striking the correct balance of relevant molecular properties of hydrophilicity, charge, linker length etc. Unsurprisingly, the reduced affinity and more lipophilic nature of the compounds resulted in negligible in vivo uptake in PD-L1 overexpressing tumors for all compounds. More importantly, none of the four candidates yielded any substantial contrast between target-positive and target-negative PC3 tumors. Interestingly, compound Al[^18^F]F-**36**, with a *K*_i_ of 150 nM and compound Al[^18^F]F-**37** with > 1 µM showed no difference in PC3 PD-L1 tumor uptake. Several of our previous chelator compounds had affinities in the high nanomolar range but showed at least some contrast between target-positive and target-negative tumors. Again, we hypothesize that affinity of biphenyl-based PD-L1 inhibitors is an important predictor of binding, however, not the only one. Metabolization and circulation time also contributes to in vivo performance. All four compounds are quickly excreted, as relatively low liver but high gall bladder and intestinal distribution at 1.5 h p.i. indicate. At the same time, little signal in the heart and ascending aortae supports the short circulation and fast excretion time of all compounds. Interestingly, compound Al[^18^F]F-**37** exhibited some activity in the bladder, implying at least some renal clearance. Although speculative, both fast excretion and short circulation time could explain low tumor uptake to an extent. Previously investigated compounds showed longer circulation times, which could at least partially explain their higher tumor uptake (Krutzek et al. 2023b, 2023c). However, considering the overall unfavorable properties of all tracers, we did not investigate later time points in vivo. Additionally, molar activities of all four compounds were relatively low (5–18 GBq/μmol). It can be hypothesized that this could contribute to the negligible specific tumor uptake. Other groups previously demonstrated that a high molar activity affects tumor uptake favorably, e.g. for PSMA (Piron et al. 2021) and FDOPA (Stormezand et al. 2021). However, we consider this effect less important in comparison to affinity and, most importantly, overall structure. This assumption is based on our extensive experience with the copper-64 labeled compounds. There, compounds with similarly low affinity did indeed yielded specific tumor uptake, as demonstrated by a positive contrast to the target-negative xenograft.

While the covalently labeled compounds [^18^F]F-**2** and [^18^F]F-**3** appeared very stable to defluorination, likely due to the strong C-F-bond, the RESCA based compounds showed considerable uptake to osteal structures, such as skull, spine and joints. This finding is consistent with in vitro stability studies, which demonstrated up to 9% degradation of the Al[^18^F]F2⁺-RESCA complex over a 4-h period. The observed degradation is likely due to decomplexation of Al[^18^F]F2⁺ from the RESCA chelator. While RESCA offers advantageous radiolabeling conditions—such as low temperatures and short reaction times—it is associated with suboptimal kinetic inertness, a known limitation of acyclic chelators (Price and Orvig 2014).

Taken together, these new ^18^F-labeled biphenyl-based inhibitors lack affinity and exhibit unfavorable in vivo properties, making them unsuitable as small-molecule PD-L1 radiotracers. Increasing hydrophilicity by introducing additional sulfonic acid groups, along with employing a longer linker to distance the radiolabel from the active unit, could be a promising strategy to improve the in vitro and in vivo performance of ^18^F-labeled PD-L1 small-molecule radioligands.

## Conclusions

In summary, we report the synthesis, radiochemistry, and biological evaluation of four biphenyl-based small-molecule PD-L1 radioligands using fluorine-18 radiochemistry. Following the organic synthesis of the precursors, radiolabeling was achieved either via prosthetic groups using covalent ^18^F-fluorination or through chelation of Al[^18^F]F^2^⁺ with the RESCA chelator. Once the fluorination strategies were successfully established, the radioligands were assessed for in vitro stability, binding affinity, and in vivo performance in a tumor-bearing mouse model. The radioligands [^18^F]F-**2** and [^18^F]F-**3**, modified with azido-glucosyl and PEG2-based prosthetic groups, respectively, showed excellent in vitro stability. The chelator-based compounds Al[^18^F]F-**36** and Al[^18^F]F-**37** also demonstrated good stability over a 4-h time course. Despite maintaining the core binding motif and limiting modifications to the molecules’ periphery, low or no binding affinity toward PD-L1 was observed. These findings were supported by PET imaging studies, which revealed rapid excretion, hepatobiliary clearance, and a lack of PD-L1-specific tumor uptake. To address the low binding affinity, increasing the distance between the radiolabel and the binding motif—e.g., through a PEG linker—may prove beneficial. Based on log *D*_7.4_ values, introducing more solubilizing units could enhance in vivo performance by promoting renal clearance, as demonstrated in our previous studies. Nonetheless, the robust fluorination methods established via prosthetic group labeling and Al[^18^F]F^2+^-chelation provide a valuable foundation for the development of improved PD-L1 radioligands in future work.

## Methods

### Organic chemistry

#### General remarks

All manipulations that needed the exclusion of oxygen and moisture were conducted under an argon gas atmosphere using heat-gun dried flasks and the Schlenk technique. Solvents and chemicals were purchased from Acros Organics, abcr GmbH, Sigma-Aldrich Laborchemikalien GmbH, Fisher Scientific and were utilized without any further purification. Deuterated solvents for NMR studies were obtained from Deutero GmbH. The anhydrous solvents DMF and methanol were acquired from Sigma-Aldrich Laborchemikalien GmbH in Sure/Seal™ bottles. Thin-layer chromatography (TLC) analysis was carried out on pre-coated Merck plates (silica gel 60 F254, Art 5715, 0.25 mm), and visualized using UV light. NMR spectroscopy was performed on either an Agilent DD2-400 MHz NMR or an Agilent DD2-600 MHz NMR spectrometer with ProbeOne. Chemical shifts of ^1^H and ^13^C signals were reported in parts per million (ppm) at 25 °C using TMS as the internal standard. Spectra were calibrated to the respective solvent signal. Mass spectrometry (MS) was conducted with the Xevo TQ-S mass spectrometer by using ESI (electrospray ionization). High-resolution mass spectrometry (HR-MS) was performed with the Nanomate Triversa/Q-Exactive HF by using ESI for final compounds **2**, **3**, **36** and **37**. For all intermediates, high resolution mass spectra were obtained on a Q-TOF MS using electrospray ionization: Agilent 1260 Infinity II HPLC (Santa Clara, California, USA; pump G7104C, autosampler G7129C, column oven G7116A, DAD detector G7117C) coupled to γ detector Gabi Star (Raytest Isotopenmeßgeräte GmbH, Straubenhardt, Germany) followed by accurate mass Revident Q-TOF LC/Q-TOF G6575A. The measurements were performed via a chromatographic separation using an Poroshell 120 EC-C18 column (2.7 µm, 3 × 50 mm) and the following gradient of A/B *t*_0 min_ 5/95 – *t*_1 min_ 95/5 – *t*_1.5 min_ 95/5 – *t*_2.0 min_ 5/95 – *t*_4.0 min_ 5/95. A reference mass solution containing hexakis(1H,1H,3H-tetrafluoropropoxy)phosphazene and purine was continuously co-injected via dual AJS ESI source. The system was operated using Agilent Masshunter Workstation 3.6 – LC/MS data aquisition software (Version 12.0) and data evaluation was performed using Agilent Masshunter Workstation 3.6 Qualitative Analysis software (Version 12.0 Update 1).

Analytical reversed-phase HPLC was performed using an Agilent C18 column (Agilent Zorbax 300SB-C18, 100 mm × 4.6 mm) with acetonitrile/water (0.1% TFA each) as the mobile phase. Semi-preparative reversed-phase HPLC separations were conducted on the Knauer Azura system using Zorbax SB C-18 5 μm 80 Å, 9.4 × 250 mm columns as the stationary phase, with acetonitrile/water (0.1% TFA each) as the mobile phase.

### General procedures

#### GP-1: reductive amination

The aldehyde (1.0 equiv.) and methylamine (33% in EtOH, 20.0 equiv.) were stirred in a 1:1 mixture of abs. THF/MeOH at room temperature for 10 min. Afterwards, the solution was cooled to 0 °C and sodium cyanoborohydride (3 equiv.) were added. The mixture was allowed to reach room temperature and was stirred then for 16 h. After complete conversion, monitored by TLC, the solvent was removed and ethyl acetate and water (1:1) were added. The phases were separated, and the aqueous one extracted three times with water. The combined organic phases were washed with brine, dried over sodium sulfate and filtered. The solvent was removed *in vacuo* and the residue purified by column chromatography on SiO_2_ to yield the secondary amine.

### GP-2: HATU-coupling

The carboxylic acid (1.2 equiv.), HATU (1.2 equiv.), HOBt (1.2 equiv.) and DIPEA (3.0 equiv.) were dissolved in abs. DMF. The amine (1.0 equiv.) was added in abs. DMF and the reaction mixture was stirred at room temperature for 3 h. After complete conversion (monitored by HPLC, System A), the solvent was removed under reduced pressure and the residue was purified with semi-preparative HPLC. After lyophilization, the final compound was obtained as a colorless, fluffy powder.

### GP-3: HATU-coupling and Fmoc-deprotection

The carboxylic acid (1.2 equiv.), HATU (1.2 equiv.), HOBt (1.2 equiv.) and DIPEA (3.0 equiv.) were dissolved in abs. DMF. The amine (1.0 equiv.) was added in abs. DMF and the reaction mixture was stirred at room temperature for 3 h. After complete conversion (monitored by HPLC, System A), piperidine (20 equiv.) was added and the reaction mixture was stirred at room temperature for one more hour. After complete cleavage of the Fmoc-group (monitored by HPLC, System A), the solvent was removed *in vacuo*. The residue was taken up in MeCN/H_2_O (1:1), filtered to remove cleavage products and then purified by semi-preparative HPLC. After lyophilization, the final compound was obtained as a colorless, fluffy powder.

### HPLC systems

#### System A

RP-HPLC, analytical (Agilent Zorbax 300 C-18.5 µm, 4.6 150 mm) with 10–95% MeCN (0.1% TFA) in H_2_O (0.1% TFA) with a linear gradient over 15 min, 1 mL/min.

#### System B

RP-HPLC, analytical (Agilent Zorbax 300 C-18.5 µm, 4.6 × 200 mm) with 10–95% MeCN (0.1% TFA) in H_2_O (0.1% TFA) with a linear gradient over 30 min, 1 mL/min.

#### System C

RP-HPLC, analytical (Waters XTerra MS C18 2.5 µm, 3.0 ×  50 mm) with 0–100% 20 mM NH_4_OAc in MeCN (15% H_2_O) in 20 mM NH_4_OAc in H_2_O (15% MeCN) with a linear gradient over 13 min, 1 mL/min.

#### Synthetic procedures

*1-(5-Chloro-4-((2,2'-dimethyl-3'-(prop-2-yn-1-yloxy)-[1,1'-biphenyl]−3-yl)methoxy)−2-((5-(methylsulfonyl)pyridin-3-yl)methoxy)phenyl)-N-methylmethanamine (* ***5*** *)* 
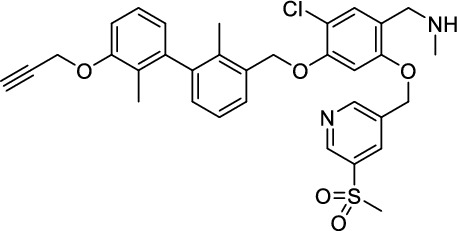


The aldehyde **4** (314 mg, 532 µmol, 1.0 equiv.) and methylamine (33%in EtOH, 437 µL, 20.0 equiv.) reacted in THF/MeOH (8 mL) according to GP-1. After column chromatography on SiO_2_ (CH_2_Cl_2_:MeOH:NH_4_OH, 94:5:1, *R*_f_ = 0.3), the amine **5** (270 mg, 447 µmol, 84%) was isolated as yellowish oil. ^1^H-NMR (400 MHz, CDCl_3_) *δ* = 9.05 (d, ^4^* J* = 2.2 Hz, 1H), 8.87 (d, ^4^* J* = 1.9 Hz, 1H), 8.29 (t, ^4^* J* = 2.0 Hz, 1H), 7.39 (d, ^3^* J* = 7.2 Hz, 1H), 7.11–7.20 (m, 2H), 7.04–7.06 (m, 1H), 6.91 (d, ^3^* J* = 8.1 Hz, 1H), 6.72 (d, ^3^* J* = 7.5 Hz, 1H), 6.59 (s, 1H), 5.09 (s, 4H), 2.08 (d, ^4^* J* = 2.5 Hz), 3.65 (s, 2H), 3.06 (s, 3H), 2.36 (t, ^4^* J* = 2.4 Hz, 1H), 2.36 (s, 3H), 2.02 (s, 3H), 1.86 ppm (s, 3H). ^13^C-NMR (101 MHz, CDCl_3_) *δ* = 171.2, 155.9, 155.2, 154.1, 153.1, 153.0, 148.1, 142.9, 142.2, 137.1, 134.6, 134.4, 134.1, 133.3, 131.4, 131.3, 129.7, 127.5, 126.0, 125.6, 125.6, 125.5, 122.7, 122.3, 115.9, 110.5, 100.6, 100.5, 79.1, 79.0, 75.5, 75.4, 70.7, 67.4, 56.2, 49.8, 44.8, 35.9, 35.8, 15.7, 13.0 ppm. HR-MS (ESI^+^): Mass calculated for [M + H]^+^: *m/z* = 605,1872, measured: *m/z* = 605.1873.

*(R)−2-Amino-3-((5-chloro-4-((2,2'-dimethyl-3'-(prop-2-yn-1-yloxy)-[1,1'-biphenyl]−3-yl)methoxy)−2-((5-(methylsulfonyl)pyridin-3-yl)methoxy)benzyl)(methyl)amino)−3-oxopropane-1-sulfonic acid (* ***7*** *)* 
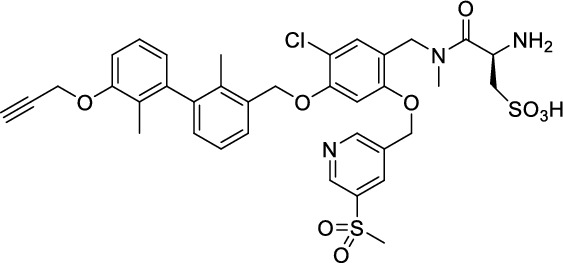


The coupling of Fmoc-l-cysteic acid (69.9 mg, 178 µmol, 1.2 equiv.), HATU (67.7 mg, 178 µmol, 1.2 equiv.), HOBt (24.0 mg, 178 µmol, 1.2 equiv.), DIPEA (75.9 µL, 446 µmol, 3.0 equiv.) and the amine **5** (90.0 mg, 149 µmol, 1.0 equiv.) in abs. DMF (3 mL) and the subsequent Fmoc-deprotection with piperidine (352 µL, 3.56 mmol, 20 equiv.) were performed according to GP-3. After semi-preparative RP-column chromatography (Agilent Zorbax SB C-18 5 µm 80 Å, 9.4 × 250 mm with 45–90% MeCN (0.1% Et_3_N) in H_2_O (0.1% Et_3_N) in a linear gradient over 45 min, 6 mL/min, *R*_t_ = 6 min) and lyophilization, **7** (86.1 mg, 114 µmol, 64%) was isolated as a colorless, fluffy powder. *R*_t_ = 12.02 min (system A), purity = 99.5%. ^1^H-NMR (400 MHz, DMSO-*d*_6_) *δ* = 9.05–9.09 (m, 2H), 8.47 (bs, 1H), 8.11 (bs, 1H), 8.04 (bs, 2H), 7.46–7.51 (m, 1H), 7.21–7.28 (m, 4H), 7.04–7.10 (m, 2H), 6.74 (d, ^3^*J* = 7.5 Hz, 1H), 5.42–5.50 (m, 2H), 5.25–5.28 (m, 2H), 4.85 (bs, 2H), 4.36–4.66 (m, 4H), 3.37 (s, 3H), 2.91–3.04 (m, 3H), 2.70–2.76 (m, 2H), 2.02–2.07 (m, 3H), 1.83 (bs, 3H), 1.24–1.27 ppm (2H). ^13^C-NMR (101 MHz, DMSO-*d*_6_) *δ* = 167.5, 167.2, 155.4, 153.6, 153.2, 147.3, 142.3, 141.4, 137.0, 134.7, 134.5, 134.3, 133.2, 133.0, 129.2, 127.8, 126.2, 125.5, 124.1, 122.1, 118.3, 117.5, 113.5, 110.9, 109.5, 100.7, 79.5, 78.1, 69.7, 67.2, 55.8, 53.6, 49.3, 48.8, 48.6, 46.7, 45.3, 43.6, 43.5, 34.9, 33.1, 18.1, 16.7, 15.3, 12.8 ppm. HR-MS (ESI^−^): Mass calculated for [M-H]^−^: *m/z* = 754.1665, measured: *m/z* = 754.1665.

*(R)−2-Amino-3-(((R)−1-((5-chloro-4-((2,2'-dimethyl-3'-(prop-2-yn-1-yloxy)-[1,1'-biphenyl]−3-yl)methoxy)−2-((5-(methylsulfonyl)pyridin-3-yl)methoxy)benzyl)(methyl)amino)−1-oxo-3-sulfopropan-2-yl)amino)−3-oxopropane-1-sulfonic acid (* ***9*** *)* 
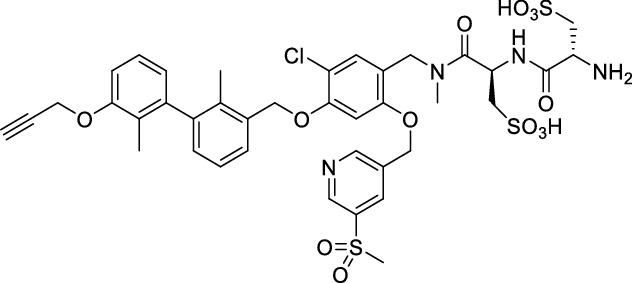


The coupling of Fmoc-l-cysteic acid (51.5 mg, 132 µmol, 1.2 equiv.), HATU (50.1 mg, 132 µmol, 1.2 equiv.), HOBt (17.8 mg, 132 µmol, 1.2 equiv.), DIPEA (56.0 µL, 329 µmol, 3.0 equiv.) and the amine **7** (83 mg, 110 µmol, 1.0 equiv.) in abs. DMF (2 mL) and the subsequent Fmoc-deprotection with piperidine (220 µL, 2.19 mmol, 20.0 equiv.) were performed according to GP-3. After semi-preparative RP-HPLC (Agilent Zorbax SB C-18 5 µm 80 Å, 9.4 × 250 mm with 40–90% MeCN (0.1% Et_3_N) in H_2_O (0.1% Et_3_N) in a linear gradient over 45 min, 6 mL/min, *R*_t_ = 7 min) and lyophilization, **9** (61.1 mg, 67.2 µmol, 61%) was isolated as a colorless, fluffy powder.*R*_t_ = 11.05 min (system A), purity = 98.6%. ^1^H-NMR (400 MHz, DMSO-*d*_6_) *δ* = 9.12 (d, ^3^*J* = 7.0, 1H), 9.01–9.07 (m, 2H), 8.47 (bs, 1H), 8.10 (bs, 3H), 7.47–7.53 (m, 1H), 7.16–7.33 (m, 4H), 7.04–7.09 (m, 2H), 6.75 (d, ^3^*J* = 7.4 Hz, 1H), 5.39–5.49 (m, 2H), 5.23–5.30 (m, 2H), 5.05–5.08 (m, 1H), 4.85–4.91 (m, 2H), 4.34–4.50 (m, 2H), 5.38 (bs, 1H), 3.38 (bs, 3H), 2.94–3.06 (m, 2H), 2.81–2.86 (m, 2H), 2.66–2.70 (m, 2H), 2.02–2.07 (m, 3H), 1.84 ppm (s, 3H). ^13^C-NMR (101 MHz, DMSO-*d*_6_) *δ* = 171.0, 170.4, 166.6, 166.5, 155.4, 155.1, 153.3, 142.4, 141.4, 136.9, 134.8, 134.4, 133.2, 129.1, 128.8, 127.7, 126.2, 125.5, 124.1, 122.1, 119.1, 118.9, 113.6, 110.9, 104.8, 100.7, 79.5, 78.1, 69.6, 67.2, 55.8, 52.1, 50.0, 47.3, 45.3, 43.6, 43.5, 35.0, 32.9, 14.3, 12.8 ppm. HR-MS (ESI^−^): Mass calculated for [M-H]^−^: *m/z* = 905.1604, measured: *m/z* = 905.1606.

*(R)−3-((5-Chloro-4-((2,2'-dimethyl-3'-(prop-2-yn-1-yloxy)-[1,1'-biphenyl]−3-yl)methoxy)−2-((5-(methylsulfonyl)pyridin-3-yl)methoxy)benzyl)(methyl)amino)−2-((R)−2-(3-(diethoxyphosphoryl)propanamido)−3-sulfopropanamido)−3-oxopropane-1-sulfonic acid (* ***10*** *)* 
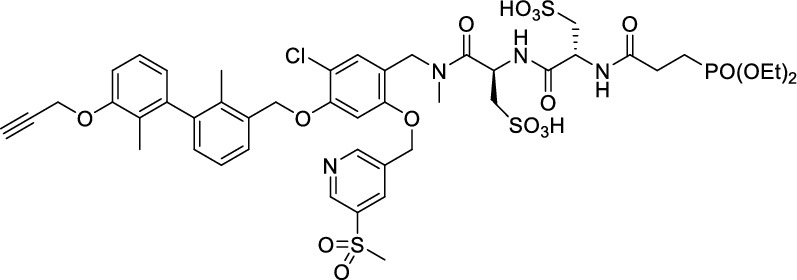


Diethyl ciliatine (13.9 mg, 66.1 µmol, 1.2 equiv.), HATU (25.1 mg, 66.1 µmol, 1.2 equiv), DIPEA (28.1 µL, 165 µmol, 3.0 equiv.) and the amine **9** (50.0 mg, 55.1 µmol, 1.0 equiv.) reacted in abs. DMF (2 mL) according to GP-2. After semi-preparative RP-HPLC (Agilent Zorbax SB C-18 5 µm 80 Å, 9.4 × 250 mm with 20–80% MeCN (0.1% Et_3_N) in H_2_O (0.1% Et_3_N) in a linear gradient over 45 min, 6 mL/min, *R*_t_ = 8 min) and lyophilization, **10** (45.5 mg,41.4 µmol, 75%) was obtained as a colorless, fluffy powder. *R*_t_ = 11.35 min (system A), purity = 98.8%. ^1^H-NMR (400 MHz, DMSO-*d*_6_) *δ* = 9.03–9.06 (m, 2H), 8.91 (bs, 2H), 8.47 (s, 1H), 8.12 (d, ^3^*J* = 6.5 Hz, 1H), 7.98–8.07 (m, 2H), 7.47–7.53 (m, 1H), 7.22–7.30 (m, 3H), 7.14–7.16 (m, 1H), 7.04–7.09 (m, 2H), 6.75 (d, ^3^*J* = 7.5 Hz, 1H), 5.39–5.47 (m, 2H), 5.23–5.29 (m, 2H), 5.01–5.03 (m, 1H), 4.77–4.93 (m, 3H), 4.36–4.46 (m, 4H),3.09 (q, ^3^*J* = 7.3 Hz, 4H), 2.82–2.92 (m, 2H), 2.50–2.70 (m, 2H), 2.23–2.29 (m, 2H), 2.02–2.04 (m, 2H), 1.88–1.96 (m, 2H), 1.84 (bs, 2H), 1.17 ppm (t, ^3^*J* = 7.3 Hz, 6H). ^13^C-NMR (101 MHz, DMSO-*d*_6_) *δ* = 171.4, 170.8, 170.3, 170.1, 155.4, 155.1, 153.6, 153.2, 147.3, 142.4, 141.4, 136.9, 134.9, 134.6, 134.5, 134.5, 134.3, 133.3, 129.2, 128.9, 127.7, 126.2, 125.5, 124.1, 122.1, 122.1, 119.2, 118.9, 113.7, 113.5, 110.9, 109.6, 100.6, 79.6, 78.2, 69.6, 67.1, 61.1, 61.0, 55.8, 52.5, 51.3, 50.7, 46.7, 45.7, 45.3, 43.6, 43.6, 35.1, 32.9, 28.4, 21.3, 19.9, 16.3, 15.3, 12.9. 8.6 ppm. ^31^P-NMR (162 MHz, DMSO-*d*_6_) *δ* = 31.15 ppm. HR-MS (ESI^−^): Mass calculated for [M-H]^−^: *m/z* = 1097.2156, measured: *m/z* = 1097.2155.

*(R)−3-((5-Chloro-4-((2,2'-dimethyl-3'-(prop-2-yn-1-yloxy)-[1,1'-biphenyl]−3-yl)methoxy)−2-((5-(methylsulfonyl)pyridin-3-yl)methoxy)benzyl)(methyl)amino)−3-oxo-2-((R)−2-(3-phosphonopropanamido)−3-sulfopropanamido)propane-1-sulfonic acid (* ***11*** *)* 
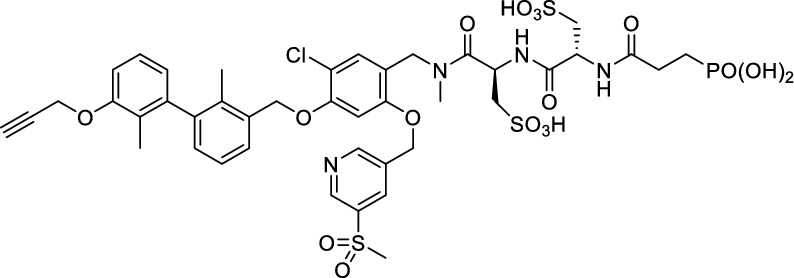


The starting material **10** (60 mg, 54.6 µmol, 1.0 equiv.) was dissolved in abs. DMF (0.5 mL) and TMSBr (144 µL, 1.09 mmol, 20.0 equiv.) was added. The reaction was stirred at room temperature for 40 h and the solvent was then removed under reduced pressure. The residue was purified by RP-HPLC (Agilent Zorbax SB C-18 5 µm 80 Å, 9.4 × 250 mm with 30–80% MeCN (0.1% TFA) in H_2_O (0.1% TFA) in a linear gradient over 45 min, 6 mL/min, *R*_t_ = 8 min) and after lyophilization, the free phosphonic acid **11** (27.6 mg, 26.5 µmol, 64%) was isolated as colorless, fluffy solid. *R*_t_ = 18.56 min (system B), purity = 98.5%. ^1^H-NMR (400 MHz, DMSO-*d*_6_) *δ* = 9.10 (s, 1H), 9.07 (s, 1H), 8.52–8.53 (m, 1H), 8.01–8.15 (m, 2H), 7.52–7.53 (m), 7.47–7.49 (m), 7.25–7.29 (m, 2H), 7.23 (t, ^3^*J* = 7.99 Hz, 1H), 7.13–7.16 (m, 1H), 7.04–7.08 (m, 2H), 6.74 (d, ^3^*J* = 7.5 Hz), 5.37–5.49 (m, 2H), 5.22–5.28 (m, 2H), 4.90–4.92 (m, 1H), 4.85 (s, 2H), 4.38–4.48 (m, 3H), 3.58 (t, ^3^*J* = 2.3 Hz, 1H), 3.40 (s, 2H), 3.37 (s, 1H), 3.03 (s, 2H), 2.90–2.93 (m, 2H), 2.75–2.79 (m, 1H), 2.61–2.65 (m, 2H), 2.28–2.31 (m, 2H), 2.07 (s, 1H), 2.02–2.04 (m, 3H), 1.78–1.84 ppm (m, 5H). ^13^C-NMR (101 MHz, DMSO-*d*_6_) *δ* = 171.2, 171.1, 171.0, 170.6, 170.0, 169.9, 155.4, 155.1, 155.0, 153.7, 153.3, 152.8, 152.5, 146.7, 146.6, 142.4, 141.4, 137.2, 137.1, 135.3, 135.1, 134.9, 134.8, 134.5, 133.8, 129.8, 129.2, 127.7, 126.2, 125.5, 124.2, 122.2, 119.1, 118.9, 118.1, 113.7, 113.6, 110.9, 100.7, 79.6, 78.2, 69.7, 67.1, 55.9, 52.3, 51.3, 50.7, 50.5, 46.8, 45.3, 43.7, 43.6, 35.0, 32.9, 29.2, 23.7, 22.8, 15.3, 12.8, 1.2 ppm. ^31^P-NMR (162 MHz, DMSO-*d*_6_) *δ* = 26.26 ppm. HR-MS (ESI^−^): Mass calculated for [(M/2)-H]^−^: *m/z* = 520.0728, measured: *m/z* = 520.0727.

*(R)−3-((5-Chloro-4-((3'-((1-((2R,3R,4S,5S,6S)−6-(fluoromethyl)−3,4,5-trihydroxytetrahydro-2H-pyran-2-yl)−1H-1,2,3-triazol-4-yl)methoxy)−2,2'-dimethyl-[1,1'-biphenyl]−3-yl)methoxy)−2-((5-(methylsulfonyl)pyridin-3-yl)methoxy)benzyl)(methyl)amino)−3-oxo-2-((R)−2-(3-phosphonopropanamido)−3-sulfopropanamido)propane-1-sulfonic acid (* ***2*** *)* 
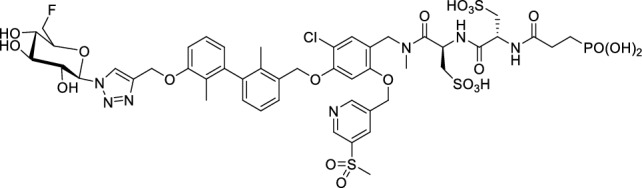


The alkyne **11** (10.0 mg, 9.58 µmol, 1.0 equiv.) and 2-azido-6-fluoroglucose (Maschauer et al. 2014) (6.0 mg, 28.8 µmol, 3.0 equiv.) were dissolved in an 1:1 mixture ^*t*^BuOH:H_2_O (1 mL). Sodium ascorbate (2.85 mg, 14.4 µmol, 1.5 equiv.), copper(II)-sulfate (0.15 mg, 0.96 µmol, 0.1 equiv.) and the THPTA-ligand (0.83 mg, 1.92 µmol, 0.2 equiv.) were premixed in water (50 µL) and the click-reagents added subsequently. The reaction was stirred at room temperature for 3 h and then directly injected in the semi-preparative RP-HPLC (Agilent Zorbax SB C-18 5 µm 80 Å, 9.4 × 250 mm with 22–80% MeCN (0.1% TFA) in H_2_O (0.1% TFA) in a linear gradient over 45 min, 6 mL/min, *R*_t_ = 10 min). After lyophilization, the ^19^F-reference compound **2** (10.8 mg, 8.64 µmol, 90%) was obtained as a fluffy colorless powder. *R*_t_ = 16.04 min (system B), purity = 97.3%. ^1^H-NMR (400 MHz, DMSO-*d*_6_) *δ* = 9.04–9.07 (m, 2H), 8.48–8.49 (m, 2H), 7.99–8.00 (m, 1H), 8.10–8.11 (m, 1H), 7.46–7.53 (m, 1H), 7.07–7.38 (m, 6H), 6.74 (d, ^3^*J* = 7.1 Hz, 1H), 5.67 (d, ^3^*J* = 9.3 Hz, 1H), 4.35–4.65 (m, 6H), 3.83–3.87 (m, 1H), 3.71–3.77 (m, 1H), 3.42–3.44 (m, 1H), 3.31–3.39 (m, 4H), 2.08 (s, 3H), 2.83–2.88 (m, 2H), 2.61–2.75 (m, 3H), 2.28–2.29 (m, 2H), 2.03–2.04 (m, 3H), 1.74–1.83 ppm (m, 5H). ^13^C-NMR (101 MHz, DMSO-*d*_6_) *δ* = 171.4, 171.1, 171.0, 170.7, 170.0, 156.2, 155.1, 153.2, 152.9, 147.0, 143.0, 142.3, 141.5, 137.0, 134.8, 134.5, 134.6, 133.5, 129.2, 126.3, 125.4, 124.0, 123.7, 121.8, 119.2, 113.7, 110.6, 109.6, 87.2, 77.3, 77.1, 76.7, 71.8, 68.3, 67.1, 61.6, 52.5, 51.2, 50.6, 46.7, 45.3, 43.6, 35.0, 32.9, 29.3, 24.1, 22.7, 15.3, 12.9 ppm. ^19^F-NMR (376 MHz, DMSO-*d*_6_) *δ* = –232.1–(–231.7) (m, 1H) ppm. ^31^P-NMR (162 MHz, DMSO-*d*_6_) *δ* = 25.51 ppm. HR-MS (ESI^+^): Mass calculated for [M + H]^+^: *m/z* = 1250.2336, measured: *m/z* = 1250.2355.

*(R)−3-((5-Chloro-4-((3'-((1-(2-(2-(2-fluoroethoxy)ethoxy)ethyl)−1H-1,2,3-triazol-4-yl)methoxy)−2,2'-dimethyl-[1,1'-biphenyl]−3-yl)methoxy)−2-((5-(methylsulfonyl)pyridin-3-yl)methoxy)benzyl)(methyl)amino)−3-oxo-2-((R)−2-(3-phosphonopropanamido)−3-sulfopropanamido)propane-1-sulfonic acid (* ***3*** *)* 
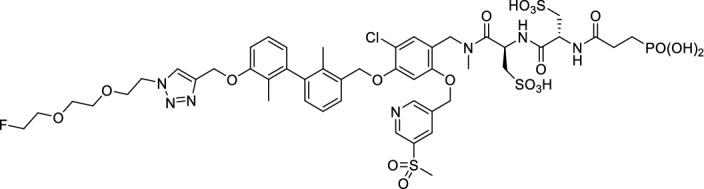


The alkyne **11** (6.00 mg, 5.75 µmol, 1.0 equiv.) and 1-azido-2-(2-(2-fluoroethoxy)ethoxy)ethane (3.1 mg, 17.3 µmol, 3.0 equiv.) were dissolved in a 1:1 mixture ^*t*^BuOH:H_2_O (1 mL). Sodium ascorbate (1.7 mg, 8.62 µmol, 1.5 equiv.), copper(II)-sulfate (0.09 mg, 0.57 µmol, 0.1 equiv.) and the THPTA-ligand (0.50 mg, 1.15 µmol, 0.2 equiv.) were premixed in water (50 µL) and the click-reagents added subsequently. The reaction was stirred at room temperature for 3 h and then directly injected in the semi-preparative RP-HPLC (Agilent Zorbax SB C-18 5 µm 80 Å, 9.4 × 250 mm with 22–80% MeCN (0.1% TFA) in H_2_O (0.1% TFA) in a linear gradient over 45 min, 6 mL/min, *R*_t_ = 12 min). After lyophilization the ^19^F-reference compound **3** (5.90 mg, 4.83 µmol, 84%) was obtained as a fluffy colorless powder. *R*_t_ = 11.79 min (system B), purity = 99.9%. ^1^H-NMR (400 MHz, DMSO-*d*_6_) *δ* = 9.04–9.07 (m, 2H), 8.49 (bs, 1H), 8.22 (s, 1H), 8.10–8.11 (m, 1H), 7.93–8.00 (m, 1H), 7.46–7.52 (m, 1H), 7.22–7.28 (m, 2H), 7.13–7.18 (m, 2H), 7.06 (d, ^3^*J* = 7.44 Hz, 1H), 6.73 (d, ^3^*J* = 7.5 Hz, 1H), 4.51–4.57 (m, 3H), 4.35–4.44 (m, 3H), 3.82–3.85 (m, 2H), 3.61–3.62 (m, 1H), 3.52–3.55 (m, 4H), 3.36–3.38 (m, 2H), 3.04 (s, 2H), 2.82–2.91 (m, 2H), 2.59–2.74 (m, 3H), 2.25–2.29 (m, 2H), 2.02–2.03 (m, 3H), 1.76–1.81 ppm (m, 5H). ^13^C-NMR (101 MHz, DMSO-*d*_6_) *δ* = 171.4, 171.1, 170.9, 170.0, 156.2, 155.1, 153.2, 152.9, 147.0, 142.9, 142.3, 141.5, 137.0, 134.8, 134.5, 134.4, 133.4, 129.1, 127.6, 126.3, 125.4, 124.7, 124.0, 121.8, 119.2, 113.7, 110.7, 100.7, 83.8, 82.2, 69.7, 69.6, 69.5, 68.7, 67.1, 61.7, 52.5, 51.2, 50.6, 49.6, 46.8, 45.3, 43.6, 43.6, 35.0, 32.8, 29.3, 24.1, 22.7, 15.3, 12.8 ppm. ^19^F-NMR (376 MHz, DMSO-*d*_6_) *δ* = –221.7–(–221.3) (m, 1H) ppm. ^31^P-NMR (162 MHz, DMSO-*d*_6_) *δ* = 25.51 ppm. HR-MS (ESI^+^): Mass calculated for [M + H]^+^: *m/z* = 1220.2595, measured: *m/z* = 1220.2606.

*3-Chloro-4-((2,2'-dimethyl-[1,1'-biphenyl]−3-yl)methoxy)benzaldehyde (* ***24*** *)* 
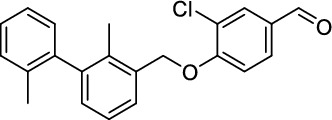


The phenol **22** (550 mg, 3.51 mmol, 1.0 equiv.), biaryl **21** (820 mg, 3.86 mmol, 1.1 equiv.) and triphenylphosphine (1.11 g, 4.22 mmol, 1.2 equiv.) were dissolved in abs. DMF (20 mL) at 0 °C. DEAD (889 µL, 4.22 mmol, 1.2 equiv.) was added in small portions and the reaction mixture was then stirred at room temperature for 16 h. After full conversion (monitored by TLC), the solvent was removed and the residue purified by column chromatography on SiO_2_ (PE:EA, 15:1, *R*_f_ = 0.25), to obtain the aldehyde **24** (581 mg, 1.66 mmol, 47%) as an orange oil. ^1^H-NMR (400 MHz, CDCl_3_) *δ* = 9.85 (s, 1H), 7.93 (d, ^4^*J* = 1.9 Hz, 1H), 7.77 (dd, ^4^*J* = 1.9 Hz, ^3^*J* = 8.5 Hz, 1H), 7.48 (d, ^3^*J* = 7.6 Hz, 1H), 7.22–7.29 (m, 4H), 7.10–7.16 (m, 3H), 5.26 (s, 2H), 2.07 (s, 3H), 2.04 ppm (s, 3H). ^13^C-NMR (101 MHz, CDCl_3_) *δ* = 189.8, 159.2, 142.7, 141.4, 136.1, 134.5, 133.8, 131.6, 130.6, 130.5, 130.0, 129.5, 127.5, 127.4, 125.8, 125.8, 124.5, 113.2, 70.3, 20.0, 15.7 ppm. HR-MS (ESI^−^): Mass calculated for [M-H]^−^: *m/z* = 349.1001, measured: *m/z* = 349.1001.

*5-Chloro-4-((2,2'-dimethyl-[1,1'-biphenyl]−3-yl)methoxy)−2-((5-(methylsulfonyl)pyridin-3-yl)methoxy)benzaldehyde (* ***25*** *)* 
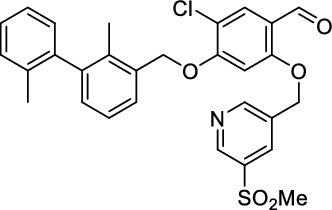


The phenol **23** (240 mg, 702 µmol, 1.0 equiv.), the biaryl **21** (164 mg, 772 µmol, 1.1 equiv.) and triphenylphosphine (221 g, 843 µmol, 1.2 equiv.) were dissolved in abs. DMF (5 mL) at 0 °C. DEAD (179 µL, 843 µmol, 1.2 equiv.) was added in small portions and the reaction mixture was then stirred at room temperature for 16 h. After full conversion (monitored by TLC), the solvent was removed and the residue purified by column chromatography on SiO_2_ (PE:EA, 9:1, *R*_f_ = 0.25), to obtain the aldehyde **25** (335 mg, 625 µmol, 89%) as an orange oil.^1^H-NMR (400 MHz, CDCl_3_) *δ* = 10.23 (s, 1H), 9.15 (bs, 1H), 8.97 (bs, 1H), 8.37 (s, 1H), 7.86 (s, 1H), 7.62–7.67 (m, 1H), 7.45–7.47 (m, 2H), 7.09–7.27 (m, 8H), 6.73 (s, 1H), 5.26 (s, 3H), 3.15 (s, 3H), 2.09 (s, 3H), 2.03 ppm (s, 3H). ^13^C-NMR (101 MHz, CDCl_3_) *δ* = 186.4, 160.2, 160.1, 153.2, 148.5, 142.8, 141.2, 134.4, 133.3, 130.6, 130.2, 129.9, 129.4, 127.5, 127.5, 125.8, 125.7, 119.3, 117.3, 98.7, 70.7, 67.9, 59.0, 44.9, 19.9, 15.8 ppm. HR-MS (ESI^+^): Mass calculated for [M + H]^+^: *m/z* = 536.1293, measured: *m/z* = 536.1295.

*1-(3-Chloro-4-((2,2'-dimethyl-[1,1'-biphenyl]−3-yl)methoxy)phenyl)-N-methylmethanamine (* ***26*** *)* 
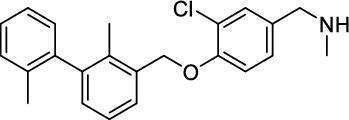


The aldehyde **25** (560 mg, 1.60 mmol, 1.0 equiv.) and methylamine (33%in EtOH, 1.30 mL, 20.0 equiv.) reacted in THF/MeOH (15 mL) according to GP-1. After column chromatography on SiO_2_ (CH_2_Cl_2_:MeOH:NH_4_OH, 94:5:1, *R*_f_ = 0.3), the amine **26** (492 mg, 1.34 mmol, 84%) was isolated as yellowish oil. ^1^H-NMR (400 MHz, MeCN-*d*_3_) *δ* = 7.45 (d, ^3^*J* = 7.5 Hz, 1H), 7.34–7.35 (m, 1H), 7.18–7.27 (m, 5H), 7.04–7.11 (m, 3H), 3.59 (s, 2H), 2.29 (s, 3H), 2.02 (s, 3H), 1.99 ppm (s, 3H). ^13^C-NMR (101 MHz, MeCN-*d*_3_) *δ* = 153.8, 143.2, 142.5, 136.8, 136.2, 135.8, 135.8, 130.8, 130.7, 130.4, 130.2, 128.7, 128.7, 128.4, 126.7, 126.5, 123.0, 118.3, 115.0, 70.7, 36.0, 20.0, 15.8 ppm. HR-MS (ESI^−^): Mass calculated for [M-H]^−^: *m/z* = 364.1473, measured: *m/z* = 364.1472.

*1-(5-Chloro-4-((2,2'-dimethyl-[1,1'-biphenyl]−3-yl)methoxy)−2-((5-(methylsulfonyl)pyridin-3-yl)methoxy)phenyl)-N-methylmethanamine (* ***27*** *)* 
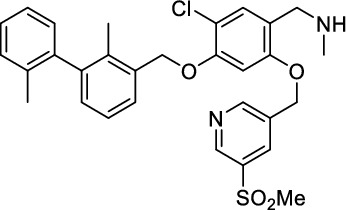


The aldehyde **25** (330 mg, 616 µmol, 1.0 equiv.) and methylamine (33%in EtOH, 503 µL, 20.0 equiv.) reacted in THF/MeOH (8 mL) according to GP-1. After column chromatography on SiO_2_ (CH_2_Cl_2_:MeOH:NH_4_OH, 92:7:1, *R*_f_ = 0.3), the amine **27** (246 mg, 446 µmol, 73%) was isolated as yellowish oil^1^H-NMR (400 MHz, MeCN-*d*_3_) *δ* = 9.05 (d, ^4^*J* = 2.1 Hz, 1H), 8.94 (d, ^4^*J* = 1.6 Hz, 1H), 8.37 (s, 1H), 7.47 (d, ^3^*J* = 7.5 Hz, 1H), 7.31 (s, 1H), 7.22–7.27 (m, 4H), 7.07 (d, ^3^*J* = 7.8 Hz, 2H), 6.90 (s, 1H), 5.25 (s, 2H), 5.20 (s, 2H), 3.65 (s, 2H), 3.14 (s, 3H), 2.32 (s, 3H), 2.06 (s, 3H), 2.00 ppm (s, 3H). ^13^C-NMR (101 MHz, MeCN-*d*_3_) *δ* = 157.2, 155.2, 154.6, 149.1, 143.8, 143.0, 138.6, 137.3, 136.5, 136.4, 135.6, 135.2, 131.4, 131.0, 130.8, 129.4, 128.9, 127.2, 127.1, 124.3, 118.9, 115.6, 102.0, 71.6, 68.9, 50.7, 45.4, 36.7, 20.7, 16.5 ppm. HR-MS (ESI^+^): Mass calculated for [M + H]^+^: m/z = 551.1766, measured: m/z = 551.1766.

*(((((9H-fluoren-9-yl)methoxy)carbonyl)(sulfo)-* *d* *-alanyl)(sulfo)-* *d* *-alanyl)(sulfo)-* *d* *-alanylglycine (* ***20*** *)* 
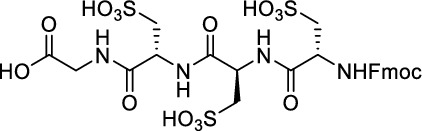


The tris(sulfonic acid) linker **20** was synthesized on standard SSP-synthesis on the N-Terminus using a 2-chlortrityl resin. *R*_t_ = 6.63 min (system A), purity = 98.9%. ^1^H-NMR (400 MHz, DMSO-*d*_6_) *δ* = 7.95 (s, 1H), 7.87 (d, ^3^*J* = 7.4 Hz, 2H), 7.72 (d, ^3^*J* = 7.4 Hz, 2H), 7.41 (t, ^3^*J* = 7.4 Hz, 2H), 7.33 (t, ^3^*J* = 7.4 Hz, 2H), 4.25–4.47 (m, 6H), 3.61–3.70 (m, 4H), 3.12–3.15 ppm (m, 1H). ^13^C-NMR (101 MHz, DMSO-*d*_6_) *δ* = 171.4, 171.1, 170.4, 162.7, 144.3, 141.1, 128.0, 127.6, 125.8, 120.4, 54.2, 52.0, 51.8, 51.5, 47.1, 42.3, 41.5, 36.2, 31.2, 18.6, 17.2, 12.7 ppm. MS (ESI^−^): HR-MS (ESI^−^): Mass calculated for [M-H]^−^: *m/z* = 749.0746, measured: *m/z* = 749.0744.

*(7R,10R,13R)−13-Amino-1-(3-chloro-4-((2,2'-dimethyl-[1,1'-biphenyl]−3-yl)methoxy)phenyl)−2-methyl-3,6,9,12-tetraoxo-7,10-bis(sulfomethyl)−2,5,8,11-tetraazatetradecane-14-sulfonic acid (* ***30*** *)* 
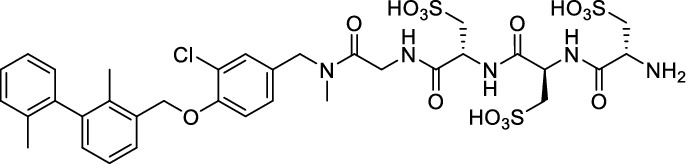


The coupling of the tris(sulfonic acid) linker **20** (90.9 mg, 121 µmol, 1.2 equiv.), HATU (46.0 mg, 121 µmol, 1.2 equiv.), HOBt (16.3 mg, 121 µmol, 1.2 equiv.), DIPEA (51.5 µL, 303 µmol, 3.0 equiv.) and the amine **26** (37.0 mg, 101 µmol, 1.0 equiv.) in abs. DMF (2 mL) and the subsequent Fmoc-deprotection with piperidine (499 µL, 5.04 mmol, 20 equiv.) were performed according to GP-3. After semi-preparative RP-column chromatography (Agilent Zorbax SB C-18 5 µm 80 Å, 9.4 × 250 mm with 20–80% MeCN (0.1% Et_3_N) in H_2_O (0.1% Et_3_N) in a linear gradient over 45 min, 6 mL/min, *R*_t_ = 8 min) and lyophilization, **30** (40.1 mg, 45.6 µmol, 45%) was isolated as a colorless, fluffy powder. *R*_t_ = 9.99 min (system A), purity = 99.9%. ^1^H-NMR (400 MHz, DMSO-*d*_6_) *δ* = 9.18 (d, ^4^*J* = 6.7 Hz, 1H), 8.22–8.27 (m), 8.05–8.10 (m), 7.49–7.50 (m, 1H), 7.26–7.32 (m, 7H), 7.07 (d, ^3^*J* = 7.3 Hz, 2H), 5.22–5.24 (m, 2H), 4.35–4.50 (m, 4H), 4.05–4.09 (m, 1H), 3.91–3.94 (m, 2H), 2.78–3.14 (m, 14H), 2.01 (s, 3H), 1.98 (s, 3), 1.63–1.65 (m, 4H), 1.54–1.55 (m, 2H), 1.18 ppm. (t, ^3^*J* = 7.3 Hz, 2H). ^13^C-NMR (101 MHz, DMSO-*d*_6_) *δ* = 170.6, 170.5, 169.9, 168.4, 168.3, 167.1, 152.9, 152.8, 141.6, 141.1, 135.3, 135.0, 134.3, 131.2, 129.8, 129.1, 129.1, 127.5, 127.3, 125.8, 125.5, 121.8, 121.5, 114.6, 114.4, 69.2, 51.5, 51.3, 51.2, 50.6, 50.4, 50.0, 49.3, 45.7, 43.8, 33.7, 33.4, 22.2, 21.7, 19.5, 15.2, 8.6 ppm. HR-MS (ESI^−^): Mass calculated for [(M/2)-H]^−^: *m/z* = 436.5717, measured: *m/z* = 436.5715.

*(7R,10R,13R)−13-Amino-1-(5-chloro-4-((2,2'-dimethyl-[1,1'-biphenyl]−3-yl)methoxy)−2-((5-(methylsulfonyl)pyridin-3-yl)methoxy)phenyl)−2-methyl-3,6,9,12-tetraoxo-7,10-bis(sulfomethyl)−2,5,8,11-tetraazatetradecane-14-sulfonic acid (* ***31*** *)* 
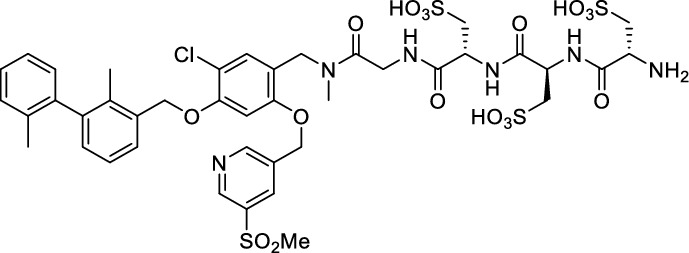


The coupling of the tris(sulfonic acid) linker **20** (89.9 mg, 120 µmol, 1.2 equiv.), HATU (45.5 mg, 120 µmol, 1.2 equiv.), HOBt (15.9 mg, 120 µmol, 1.2 equiv.), DIPEA (50.9 µL, 38.7 µmol, 3.0 equiv.) and the amine **27** (55.0 mg, 99.8 µmol, 1.0 equiv.) in abs. DMF (2 mL) and the subsequent Fmoc-deprotection with piperidine (494 µL, 4.99 mmol, 20 equiv.) were performed according to GP-3. After semi-preparative RP-column chromatography (Agilent Zorbax SB C-18 5 µm 80 Å, 9.4 × 250 mm with 20–80% MeCN (0.1% Et_3_N) in H_2_O (0.1% Et_3_N) in a linear gradient over 45 min, 6 mL/min, *R*_t_ = 7 min) and lyophilization, **31** (65 mg, 61.2 µmol, 61%) was isolated as a colorless, fluffy powder. *R*_t_ = 10.37 min (system A), purity = 99.9%. ^1^H-NMR (400 MHz, DMSO-*d*_6_) *δ* = 9.00–9.07 (m, 1H), 8.77 (bs, 1H), 8.43–8.47 (m, 1H), 8.12–8.21 (m, 1H), 8.02 (bs, 1H), 7.47–7.50 (m, 2H), 7.08–7.30 (m, 8H), 5.41–5.51 (m, 2H), 5.24–5.27 (m, 2H), 4.45 (bs, 2H), 4.31 (bs, 2H), 3.77–3.90 (m, 5H), 3.36–3.38 (m), 3.01 (bs), 2.90 (bs), 1.99–2.03 (m, 4H), 1.43–1.63 ppm (m, 10H). ^13^C-NMR (101 MHz, DMSO-*d*_6_) *δ* = 170.5, 170.5, 169.9, 168.4, 168.2, 155.2, 153.4, 153.2, 147.2, 141.7, 141.1, 136.9, 135.3, 134.4, 134.3, 133.3, 133.3, 129.8, 129.1, 127.7, 127.3, 125.8, 125.5, 119.3, 118.5, 113.5, 113.2, 100.8, 69.7, 67.1, 52.6, 51.9, 51.2, 43.8, 43.6, 34.3, 32.7, 22.3, 21.7, 19.5, 15.3 ppm. HR-MS (ESI^−^): Mass calculated for [M-H]^−^: *m/z* = 1059.165,324, measured: *m/z* = 1059.1651.

*(7R,10R,13R)−13-(3-Aminopropanamido)−1-(3-chloro-4-((2,2'-dimethyl-[1,1'-biphenyl]−3-yl)methoxy)phenyl)−2-methyl-3,6,9,12-tetraoxo-7,10-bis(sulfomethyl)−2,5,8,11-tetraazatetradecane-14-sulfonic acid (* ***34*** *).* 
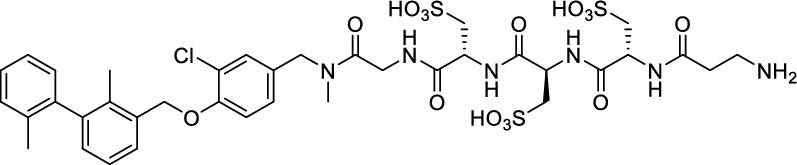


The coupling of Fmoc-β-alanine (10.9 mg, 35.1 µmol, 1.4 equiv.), HATU (13.4 mg, 35.1 µmol, 1.4 equiv.), HOBt (4.74 mg, 35.1 µmol, 1.2 equiv.), DIPEA (13.2 µL, 75.3 µmol, 3.0 equiv.) and the amine **30** (22.0 mg, 25.1 µmol, 1.0 equiv.) in abs. DMF (1 mL) and the subsequent Fmoc-deprotection with piperidine (124 µL, 1.26 mmol, 50 equiv.) were performed according to GP-3. After semi-preparative RP-column chromatography (Agilent Zorbax SB C-18 5 µm 80 Å, 9.4 × 250 mm with 20–80% MeCN (0.1% Et_3_N) in H_2_O (0.1% Et_3_N) in a linear gradient over 45 min, 6 mL/min, *R*_t_ = 8 min) and lyophilization, **34** (9.42 mg, 9.92 µmol, 40%) was isolated as a colorless, fluffy powder. *R*_t_ = 10.19 min (system A), purity = 99.9%. HR-MS (ESI^−^): Mass calculated for [M-H]^−^: *m/z* = 945.1877, measured: *m/z* = 945.1876.

*(7R,10R,13R)−13-(3-Aminopropanamido)−1-(5-chloro-4-((2,2'-dimethyl-[1,1'-biphenyl]−3-yl)methoxy)−2-((5-(methylsulfonyl)pyridin-3-yl)methoxy)phenyl)−2-methyl-3,6,9,12-tetraoxo-7,10-bis(sulfomethyl)−2,5,8,11-tetraazatetradecane-14-sulfonic acid (* ***35*** *)* 
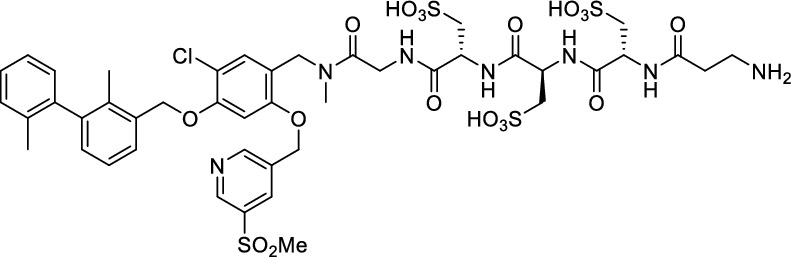


The coupling of Fmoc-β-alanine (8.5 mg, 27.1 µmol, 1.4 equiv.), HATU (10.3 mg, 27.1 µmol, 1.4 equiv.), HOBt (3.66 mg, 27.1 µmol, 1.4 equiv.), DIPEA (6.6 µL, 38.8 µmol, 3.0 equiv.) and the amine **31** (20.0 mg, 19.4 µmol, 1.0 equiv.) in abs. DMF (1 mL) and the subsequent Fmoc-deprotection with piperidine (96 µL, 969 µmol, 50 equiv.) were performed according to GP-3. After semi-preparative RP-column chromatography (Agilent Zorbax SB C-18 5 µm 80 Å, 9.4 × 250 mm with 20–80% MeCN (0.1% Et_3_N) in H_2_O (0.1% Et_3_N) in a linear gradient over 45 min, 6 mL/min, *R*_t_ = 8 min) and lyophilization, **35** (16.3 mg, 14.4 µmol, 74%) was isolated as a colorless, fluffy powder. *R*_t_ = 10.62 min (system A), purity = 99.9%. HR-MS (ESI^−^): Mass calculated for [M-H]^−^: *m/z* = 1130.2024, measured: *m/z* = 1130.2022.

*2,2'-(((1R,2R)−2-((Carboxymethyl)(4-((7R,10R,13R)−1-(3-chloro-4-((2,2'-dimethyl-[1,1'-biphenyl]−3-yl)methoxy)phenyl)−2-methyl-3,6,9,12,15,19-hexaoxo-7,10,13-tris(sulfomethyl)−2,5,8,11,14,18-hexaazaicosan-20-yl)benzyl)amino)cyclohexyl)azanediyl)diacetic acid (* ***36*** *)* 
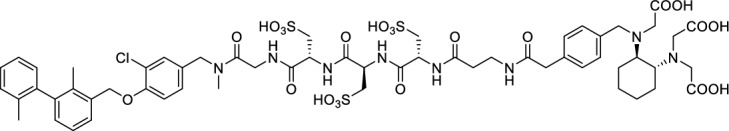


The amine **34** (9.4 mg, 9.9 µmol, 1.0 equiv.), (+)-H_3_RESCA-TFP (8.7 mg, 14.9 µmol, 1.5 equiv.) and DIPEA (5.2 µmol, 29.8 µmol, 3.0 equiv.) were dissolved in abs. DMF (0.5 mL) and stirred at room temperature for 2 h. After full conversion of the amine, the solvent was removed under reduced pressure and the residue purified by RP-HPLC (Agilent Zorbax SB C-18 5 µm 80 Å, 9.4 × 250 mm with 0–100% 20 mM NH_4_OAc in MeCN (15% H_2_O) in 20 mM NH_4_OAc in H_2_O (15% MeCN) with a linear gradient over 45 min, 6 mL/min, *R*_t_ = 14 min) to yield **36** (8.0 mg, 5.9 µmol, 59%) as a colorless, fluffy solid. *R*_t_ = 7.29 min (system C), purity = 99.9%. HR-MS (ESI^+^): Mass calculated for [M + H]^+^: *m/z* = 1365.3768, measured: *m/z* = 1365.3787.

*2,2'-(((1R,2R)−2-((Carboxymethyl)(4-((7R,10R,13R)−1-(5-chloro-4-((2,2'-dimethyl-[1,1'-biphenyl]−3-yl)methoxy)−2-((5-(methylsulfonyl)pyridin-3-yl)methoxy)phenyl)−2-methyl-3,6,9,12,15,19-hexaoxo-7,10,13-tris(sulfomethyl)−2,5,8,11,14,18-hexaazaicosan-20-yl)benzyl)amino)cyclohexyl)azanediyl)diacetic acid (* ***37*** *)* 
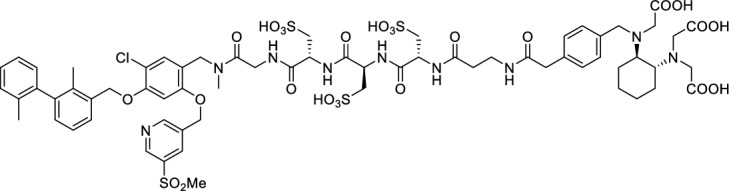


The amine **35** (16.0 mg, 14.1 µmol, 1.0 equiv.), (+)-H_3_RESCA-TFP (12.4 mg, 21.2 µmol, 1.5 equiv.) and DIPEA (7.4 µmol, 42.4 µmol, 3.0 equiv.) were dissolved in abs. DMF (0.5 mL) and stirred at room temperature for 2 h. After full conversion of the amine, the solvent was removed under reduced pressure and the residue purified by RP-HPLC (Agilent Zorbax SB C-18 5 µm 80 Å, 9.4 × 250 mm with 0–100% 20 mM NH_4_OAc in MeCN (15% H_2_O) in 20 mM NH_4_OAc in H_2_O (15% MeCN) with a linear gradient over 45 min, 6 mL/min, *R*_t_ = 14 min) to yield **37** (16.1 mg, 10.4 µmol, 73%) as a colorless, fluffy solid. *R*_t_ = 7.75 min (system C), purity = 99.9%. HR-MS (ESI^+^): Mass calculated for [(M/2) + H]^+^: *m/z* = 775.6919, measured: *m/z* = 775.7004.

### Radiochemistry

#### Covalent ^18^F-fluorination

The glycosylation of alkyne **11** was performed by modifying the procedure by Prante et al*.* (Maschauer et al. 2014).

Glucosylated radioligand [^18^F]F-**2**:

1–1.5 GBq [^18^F]fluoride (TR Flex cyclotron, in house) in 0.2 mL water was trapped on an anion exchange cartridge (Waters Accel Plus QMA Light cartridge) which was then eluted with 1 mL of an 8:2 mixture of acetonitrile/water containing 10 mg Kryptofix 2.2.2, 17.5 µL of 0.1 M solutions of K_2_CO_3_ and KH_2_PO_4_. The solution was evaporated in a stream of helium at 85 °C at 200 mbar and co-evaporated thrice with acetonitrile (500 µL each). The label precursor **12** (10 mg) in 500 µL anhydrous acetonitrile was added and the mixture was heated to 85 °C for 10 min. After cooling in a water bath, the reaction mixture was diluted with 500 µL of a 1:1 mixture of acetonitrile/water and purified by semi-preparative radio-HPLC (Agilent Zorbax SB-C18, 5 µm, 9.4 × 250 mm with 45–70% MeCN (0.1% TFA) in H_2_O (0.1% TFA) with a linear gradient over 10 min, 5 mL/min, *R*_t_ = 9 min). The obtained fraction, containing the radiolabeled product [^18^F]F-**13,** was diluted with 20 mL water, transferred to a C18-cartridge (Macherey–Nagel, 100 mg, preconditioned with 5 mL MeCN and 10 mL H_2_O) and then eluted with 700 µL ethanol. The solution was dried with helium at 200 mbar almost to dryness at 75 °C. The deprotection of the acetyl groups was initiated by addition of 250 µL of 120 mM NaOH and stirring at 75° C for 5 min. After neutralization to ~ pH 7 with 55 µL 0.5 M HCl, a mixture of 20 µL CuSO_4_ (0.2 M in H_2_O), 20 µL sodium ascorbate (120 mg/mL in H_2_O), 20 µL THPTA (0.2 M in 1:1 H_2_O/EtOH) and 20 µL alkyne **11** (10 mM in 1:1 MeCN/H_2_O) was added and the solution stirred at 75 °C for 15 min. The reaction mixture was diluted with 400 µL water and purified by semi-preparative radio-HPLC (Hamilton PRP-1, 5 µm, 10 × 250 mm with 15–45% MeCN (0.1% Et_3_N) in H_2_O (0.1% Et_3_N) with a linear gradient over 20 min, 4mL/min, *R*_t_ = 13 min). The fraction containing the triazole product [^18^F]F-**2** was diluted with 15 mL water, transferred to a C18-cartridge (Macherey–Nagel, 100 mg, preconditioned with 5 mL MeCN and 10 mL H_2_O) and then eluted with 2 mL ethanol. After removal of the solvent (75° C, helium, 200 mbar), the product was formulated with PBS ensuring less than 10% ethanol by volume for in vitro and in vivo studies. This procedure yielded 80–150 MBq [^18^F]F-**2** of the product, starting with 1–1.5 GBq [^18^F]fluoride in a total synthesis time of 85–90 min. The identity of the ^18^F-labeled product [^18^F]F-**2** was confirmed by co-injection of its ^19^F-labeled compound **2** (Supplementary Fig. S64) and the molar activity was determined by a standard curve (analytical radio-HPLC, Waters XTerra MS C18 2.5 µm, 3.0 ×  50 mm) with 0–100% 20 mM NH_4_OAc in MeCN (15% H_2_O) in 20 mM NH_4_OAc in H_2_O (15% MeCN) with a linear gradient over 13 min, 1 mL/min.

Pegylated radioligand [^18^F]F-**3**:

1–1.5 GBq [^18^F]fluoride (TR Flex cyclotron, in house) in 0.2 mL water was trapped on an anion exchange cartridge (Waters Accel Plus QMA Light cartridge) which was then eluted with 1 mL of an 8:2 mixture of acetonitrile/water containing 10 mg Kryptofix 2.2.2, 17.5 µL of 0.1 M solutions of K_2_CO_3_ and KH_2_PO_4_. The solution was evaporated in a stream of helium at 85 °C at 200 mbar and co-evaporated thrice with acetonitrile (500 µL each). The label precursor **15** (3 mg) in 500 µL anhydrous acetonitrile was added and the mixture was heated to 85 °C for 10 min. After cooling in a water bath, the reaction mixture was diluted with 500 µL of a 1:1 mixture of acetonitrile/water and purified by semi-preparative radio-HPLC (Agilent Zorbax SB-C18, 5 µm, 9.4 × 250 mm with 30–65% MeCN (0.1% TFA) in H_2_O (0.1% TFA) with a linear gradient over 10 min, 5 mL/min, *R*_t_ = 7 min). The obtained fraction, containing the radiolabeled product [^18^F]F-**16,** was diluted with 20 mL water, transferred to a polymer-cartridge (Merck KGaA, 200 mg, preconditioned with 5 mL MeCN and 10 mL H_2_O) and then eluted with 2 mL diethylether. The solution was dried with helium at 200 mbar to dryness at 40 °C, followed by the addition of 20 µL CuSO_4_ (0.2 M in H_2_O), 20 µL sodium ascorbate (120 mg/mL in H_2_O), 20 µL THPTA (0.2 M in 1:1 H_2_O/EtOH) and 20 µL alkyne **11** (10 mM in 1:1 MeCN/H_2_O). The reaction mixture was stirred at 75 °C for 15 min, diluted with 400 µL water and purified by semi-preparative radioHPLC (Hamilton PRP-1, 5 µm, 10 × 250 mm with 15–45% MeCN (0.1% Et_3_N) in H_2_O (0.1% Et_3_N) with a linear gradient over 20 min, 4 mL/min, *R*_t_ = 13 min). The fraction containing the triazole product [^18^F]F-**3** was diluted with 15 mL water, transferred to a C18-cartridge (Macherey–Nagel, 100 mg, preconditioned with 5 mL MeCN and 10 mL H_2_O) and then eluted with 2 mL ethanol. After removal of the solvent (75 °C, helium, 200 mbar), the product was formulated with PBS ensuring less than 10% ethanol by volume for in vitro and in vivo studies. This procedure yielded 120–180 MBq [^18^F]F-**3** of the product starting with 1–1.5 GBq [^18^F]fluoride in a total synthesis time of 75–85 min. The identity of the ^18^F-labeled product [^18^F]F-**3** was confirmed by co-injection of its ^19^F-labeled compound **3** (Supplementary Fig. S65) and the molar activity was determined by a standard curve (analytical radio-HPLC, Waters XTerra MS C18 2.5 µm, 3.0 ×  50 mm) with 0–100% 20 mM NH_4_OAc in MeCN (15% H_2_O) in 20 mM NH_4(._OAc in H_2_O (15% MeCN) with a linear gradient over 13 min, 1 mL/min.

Al[^18^F]F^2+^-labeling:

2.5–4 GBq [^18^F]fluoride (TR Flex cyclotron, in house) in 4 mL water were trapped on an anion exchange cartridge (Waters Accel Plus QMA Light cartridge, preconditioned with 5 mL of water) and eluted with 500 µl of HEPES (1 M, adjusted to pH 4.7). The eluate was incubated with 10 µL of AlCl_3_ in water (2 mM) for 10 min at room temperature. Then, ca. 70 µL (~ 200 MBq) of this solution was added to a mixture of ca. 70 µL ethanol and 10 µL of a 1 µM aqueous solution of the respective precursor **36** or **37** (10 nmol). The resulting reaction mixture was incubated at 50 °C for 10 min, cooled to room temperature and then used for in vitro and in vivo studies. Molar activities ranged between 5 to 18 GBq/μmol.

A minor quantity of the resultant mixture was applied onto iTLC-SG chromatography paper. The paper was developed in a TLC chamber with water/methanol (1:1) as a mobile phase and then analyzed with a radioisotope thin layer analyzer (Rita Star, Elysia-Raytest GmbH, Straubenhardt). The efficiency of the labeling process was determined by calculating the ratio between the product (*R*_f_ = 0.9) and unbound Al[^18^F]F^2+^-complex (*R*_f_ = 0) through integration of their respective peak areas. Quantitative labeling was achieved for both radiotracers.

### *Log D*_*7.4*_* determination*

The *n*-octanol/water distribution coefficients for the radioligands were determined using the shake-flask method. Each experiment was conducted in triplicate. To initiate the experiment, 30 µL of the labeling mixture was combined with 570 µL of PBS (pH 7.4) and 600 µL of *n*-octanol in a 1.5 mL Eppendorf-Tube. The resulting mixture was shaken at room temperature at 1500 rpm for 5 min before centrifugation. From each phase, an aliquot was taken and radioactivity was measured on a γ-counter (ISOMED 2160). Subsequently, mean values were calculated and adjusted for background activity. The log *D*_7.4_ value was determined utilizing the following equation:

$$\text{log}{D}_{7.4}=\text{log}(\frac{{A}_{n-octanol}}{{A}_{PBS }})$$

### Proteolytic stability studies

For proteolytic stability studies, a solution of the radiolabeled compound was diluted with human serum in a ratio of 1:10. This mixture then underwent incubation at 37 °C while being shaken at 300 rpm. At each designated timepoint (1, 24, and 4 h), an aliquot was withdrawn and added to a detergent solution (water with 20% *v*/*v* EtOH, 5% *v*/*v* 5 mM aqueous EDTA, 0.5% *v*/*v* Triton X-100, 0.1% *m*/*v* saponin, 0.05% *v*/*v* 0.5 mM aqueous *o*-phenanthroline) in a ratio of 1:2 for precipitation of serum proteins. The mixture was cooled on ice for 5 min, followed by centrifugation at 12,000 g for 5 min. A minor portion of the resulting supernatant was subjected to analytical radio-HPLC (reversed-phase, Phenomenex Jupiter 300 C-18, 5 µm, 4.6 × 250 mm) with 10–95% acetonitrile (0.1% TFA) in water (0.1% TFA) in a linear gradient over 15 min, 1 mL/min, γ-detection). The assessment and graphical representations were performed using OriginPro® 9.0.

### Cell lines and cell culture

Employed cell lines [PC3 cells transduced to overexpress human PD-L1 (PC3 PD-L1) or with a mock construct (PC3 mock) were described in detail before (Krutzek et al. 2023b). All cells were cultured under identical conditions (RPMI-1640 medium with 10% fetal calf serum, 1% Penicillin/Streptomycin, 1% alanine/glutamine and 1% non-essential amino acids) under normoxic conditions (5% CO_2_, 37 °C). Cells were usually passaged at approximately 90% confluency. Maximum number of passages was ~ 20.

Prior to competition binding experiments, cells were detached with 0.05% Trypsin–EDTA (Gibco, USA), trypsinized and counted (CASY1 cell counter, Schaerfe System, Reutlingen, Germany). Cells were then diluted in medium to ~ 320,000 cells/mL and seeded to 48 well plates (Falcon Multiwell #353078, ThermoFisher, Karlsruhe, Germany) for at least one day.

### Competition binding assay

For the determination of inhibitory constant (*K*_i_), competition binding experiments were conducted in a minimum of two independent experiments, with each data point formed from triplicates. For this purpose, all described compounds competed with a previously reported biphenyl-based PD-L1 small molecule radioligand (Krutzek et al. 2023b).

Human PD-L1 transduced PC3 cells were grown under conditions described above. For the experiments, plates were allowed to reach room temperature followed by cooling on ice (each 15 min). 200 µL of assay buffer (PBS) replaced the culture. For each compound, total binding (TB) of the radioligand was determined, along with 300 µM of BMS-1166 (synthesized according to reference (Guzik et al. 2017) (dissolved in DMSO, resulting in 0.03% *v*/*v*) for estimation of nonspecific binding (NSB).

After the 15-min preincubation, 200 µL of radioactive tracer (concentration: 30 nM, ~ 1.5 times of determined *K*_D_) containing the non-radioactive compounds (14 dilutions from 120 pM to 1 µM) was added to each well. Incubation for 90 min followed, wells were then washed with ice-cold assay buffer (3 washes) and cells subjected to lysis (500 µL of 0.1 M NaOH + 1% SDS). The radioactivity of 400 µL lysate was assessed via a gamma counter (Perkin Elmer Wizard 1480). Binding to polystyrene was determined on an extra plate without any cells under identical conditions. Activity and therefore concentration of the radioligand stock solution was confirmed via 50 µL samples. Data was corrected for radioactive decay to a reference time (end of radiolabeling). The protein content of lysates from a control plate (seeded from the same cell suspension, incubated under identical conditions) was quantified using a BCA assay, and the mean value (µg/mL) was employed for this specific dataset. Via mean protein content and molar activity, final values (pmol/mg protein) were computed from the CPM measurements. *K*_i_ was fitted via a One site model (GraphPad Prism 10), using the previously determined *K*_D_ for the radiolabeled compound and the actual concentration of the radioligand.

### Animals, biodistribution and PET imaging

Male athymic NMRI-nude mice (Rj:NMRI-Foxn1nu, Charels River, Germany) aged 8 to 16 weeks were used to investigate pharmacokinetics and tumor uptake. For subcutaneous injection of cells, general anesthesia was induced with ~ 10% desflurane (Baxter, Deerfield, IL, USA) in 30 vol% oxygen + air at controlled 37 °C. Mice were injected with 3–5 × 10^6^ PC3 PD-L1 and PC3 mock cells (in 50 µL PBS + 50 µL Matrigel, Corning, Glendale, CA, USA) into the right and left thigh, respectively. Tumor growth was monitored (3 times per week) using calipers, and animals enrolled in PET scans once tumors reached 7 mm.

Under general anesthesia, PET and X-ray computed tomography (CT) scans were conducted using a small animal PET/CT scanner (nanoScan PET/CT, Mediso, Budapest, Hungary), with four animals imaged simultaneously. CT images were used for attenuation correction and anatomical referencing.

The radiotracer candidates were diluted in 200–300 µL sterile 0.9% NaCl (and up to 2% EtOH, pH 6–7) and intravenously delivered over 30 s through a lateral tail vein catheter. PET acquisition (2 h p.i.) was started simultaneously with the administration. Injected activities were between 5 and 12 MBq (with molar activities 5–18 GBq/µmol). If animals were subjected to an additional scan later, the minimum time in between was > 3 days to allow decay and full metabolization.

The acquired three-dimensional list mode data were sorted and binned within a 400–600 keV energy window, forming 35 time frames (15 × 10 s, 5 × 30 s, 5 × 60 s, 4 × 300 s, 3 × 600 s, 3 × 1200 s). Reconstruction was performed with Mediso’s Tera-Tomo™ 3D algorithm, with a voxel size of 0.4 mm and corrections for decay, scatter, and attenuation. The images were subjected to post-processing and analysis using Rover software (ABX GmbH, Radeberg, Germany), and are shown as maximum intensity projections (MIPs) at the specified timepoint and scaling.

Three-dimensional volumes of interest were defined using fixed thresholding at 35–40% of the maximum measured intensity. Standardized uptake values (SUV = [MBq detected activity/mL tissue]/[MBq injected activity/g body weight], in mL/g) were then calculated within the selected volumes of interest, e.g. PD-L1 and mock tumors, as well as other relevant and metabolizing organs.

### Data and statistical analysis

The data are expressed as the mean ± standard deviation. If applicable, statistical analyses were conducted using GraphPad Prism, version 10 (GraphPad Software Inc., San Diego, CA, USA).

## Supplementary Information

Additional file 1.

## Data Availability

The datasets generated during and/or analyzed during the current study are available from the corresponding author on reasonable request.

## Abbreviations

abs.: Absolute
BSA: Bovine serum albumin
PET: Positron emission tomography
DIPEA: Diisopropylethylamine
DMEAD: Di-2-methoxyethylazodicarboxylate
HATU: Hexafluorophosphate azabenzotriazole tetramethyl uronium
HEPES: (4-(2-Hydroxyethyl)-1-piperazineethanesulfonic acid)
HOBt: 1-Hydroxybenzotriazol
MEM: 2-Methoxyethoxymethyl
NODA-GA: 4-(4,7-Bis(2-(*tert*-butoxy)-2-oxoethyl)-1,4,7-triazacyclononan-1-yl)-5-(*tert*-butoxy)-5-oxopentanoic acid
PD-1: Programmed Cell Death Protein 1
RESCA: Restrained complexing agent
PD-L1: Programmed Death Ligand 1
SUV: Standard up-take value
TES: Triethylsilane
