# Supplementary material for: Structure–activity relationship of 18F-labeled PD-L1-targeting small molecule ligands: impact of radiolabeling strategy on affinity and in vivo performance
Source: EJNMMI Radiopharm Chem. 2025 Jul 1;10:33. doi: 10.1186/s41181-025-00359-2 (PMC12214158; doi:10.1186/s41181-025-00359-2)
Supplement: Supplementary file 1 — Additional file 1. [file 41181_2025_359_MOESM1_ESM.docx]

**Structure****–Activity Relationship of ^18^F-Labeled PD-L1-Targeting Small Molecule Ligands: Impact of Radiolabeling Strategy on Affinity and In Vivo Performance**

Fabian Krutzek^1,^ ^†^, Cornelius K. Donat^1^, Sven Stadlbauer^1,2,^*

^1^Helmholtz-Zentrum Dresden-Rossendorf, Institute of Radiopharmaceutical Cancer Research, Bautzner Landstraße 400, D-01328 Dresden, Germany

^2^School of Science, Faculty of Chemistry and Food Chemistry, Technical University Dresden, 01069 Dresden, Germany

*Corresponding author: Sven Stadlbauer, s.stadlbauer@hzdr.de, Helmholtz-Zentrum Dresden-Rossendorf, Institute of Radiopharmaceutical Cancer Research, Bautzner Landstrasse 400, D-01328 Dresden, Germany.

† Current address: Chemical Biology Program, Memorial Sloan Kettering Cancer Center, 1275 York Avenue, New York, NY 10065, USA.

Content

[1. ^1^H, ^13^C, ^19^F and ^31^P NMR spectra 2](#_Toc199697787)

[2. HPLC-chromatograms 20](#_Toc199697788)

[3. High ResolutionMass Spectra 25](#_Toc199697789)

[4. Radiochemistry 34](#_Toc199697790)

[5. Biology 38](#_Toc199697791)

# ^1^H, ^13^C, ^19^F and ^31^P NMR spectra


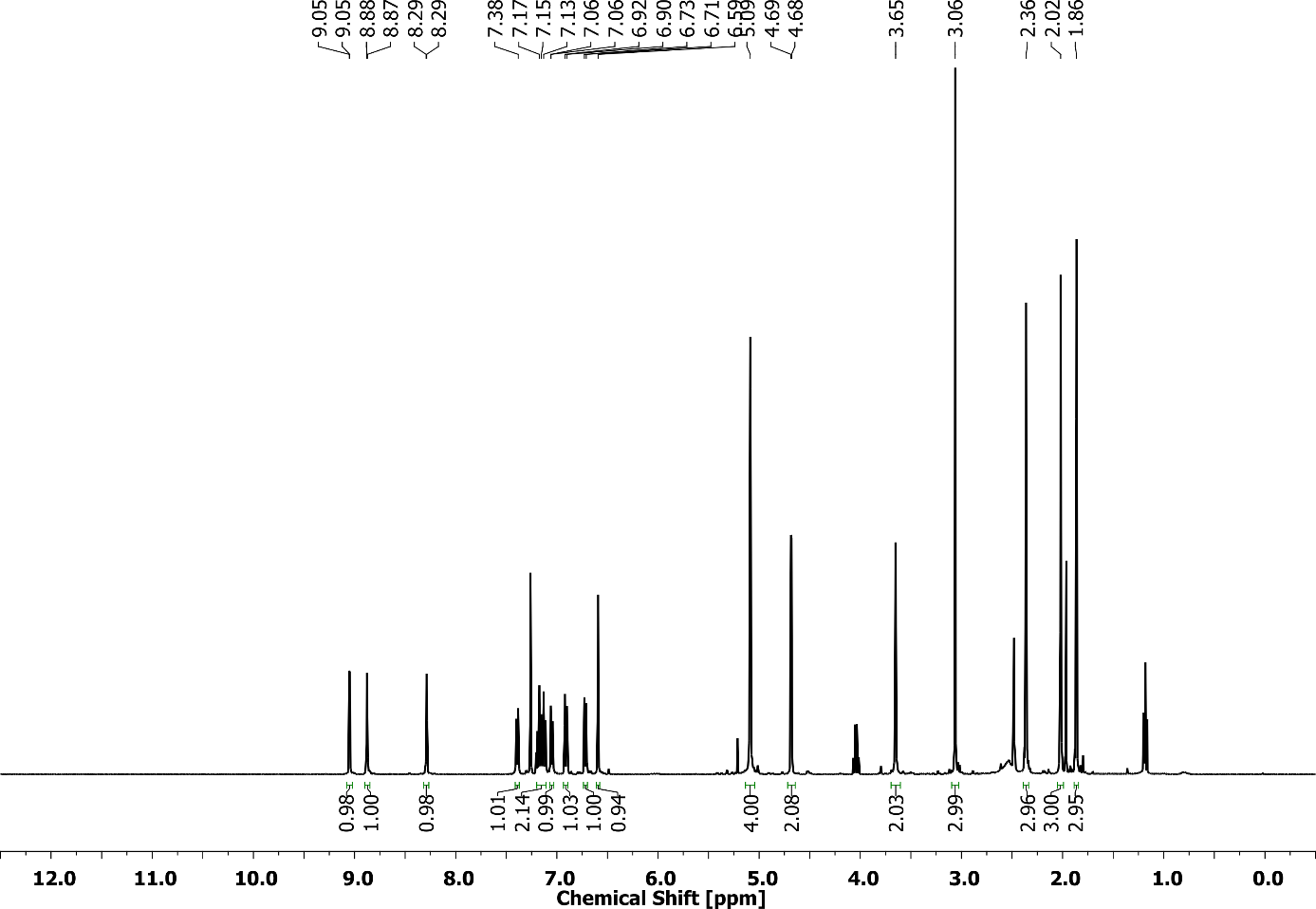


Fig. S1: ^1^H NMR spectrum (CDCl_3_, 400 MHz, 298 K) of compound 5.


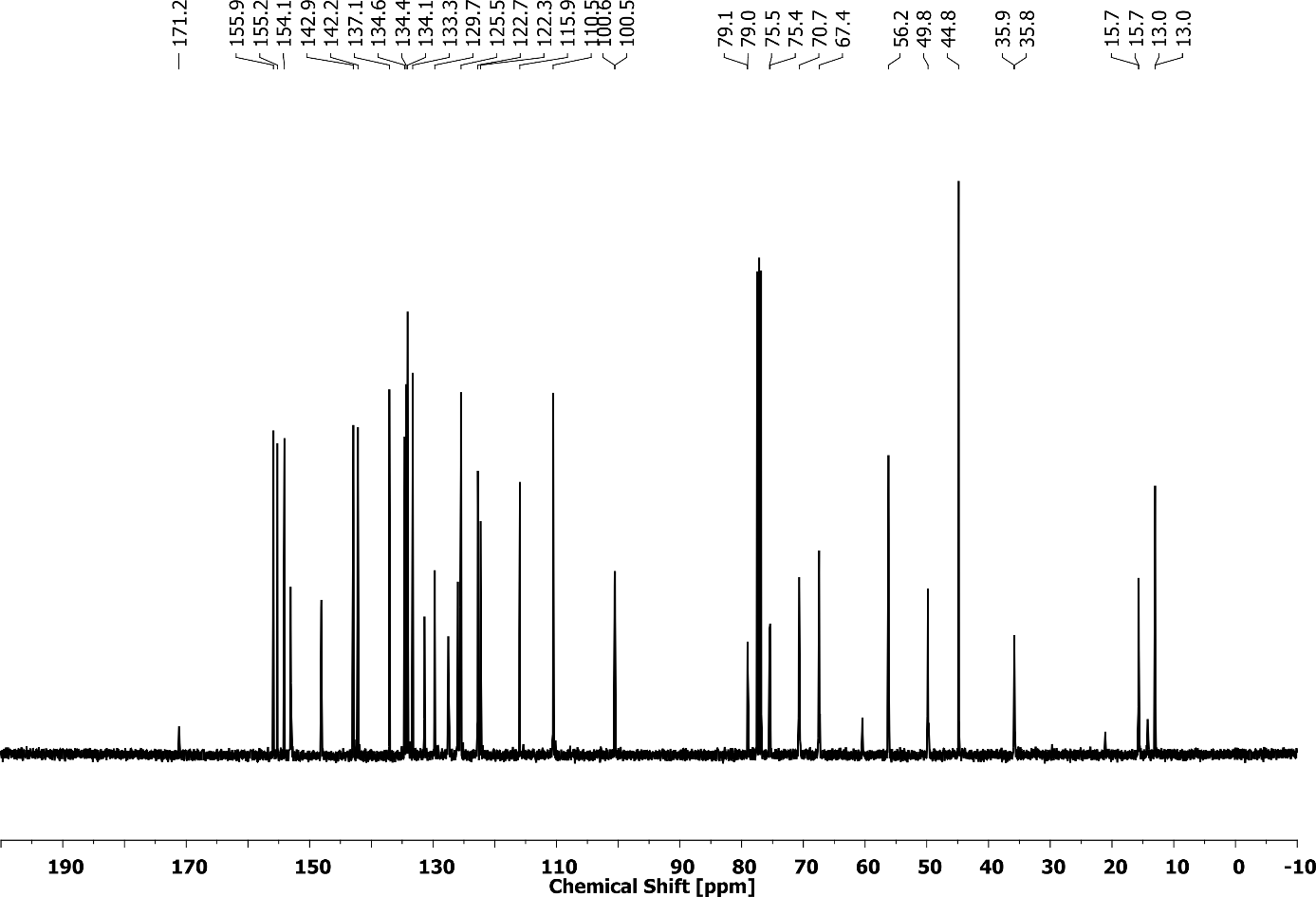


Fig. S2: ^13^C NMR spectrum (CDCl_3_, 101 MHz, 298 K) of compound 5.


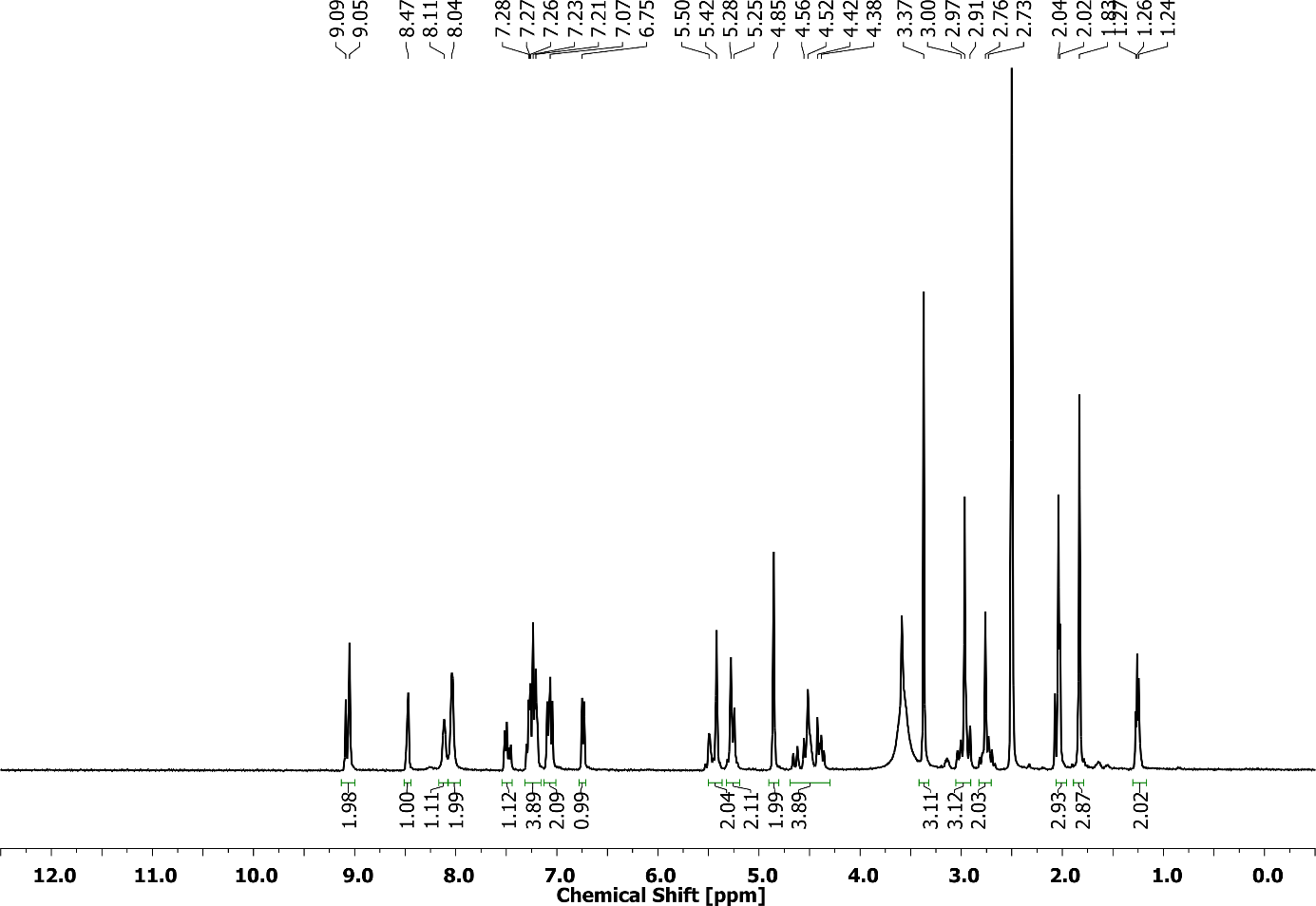


Fig. S3: ^1^H NMR spectrum (DMSO-*d*_6_, 400 MHz, 298 K) of compound 7.


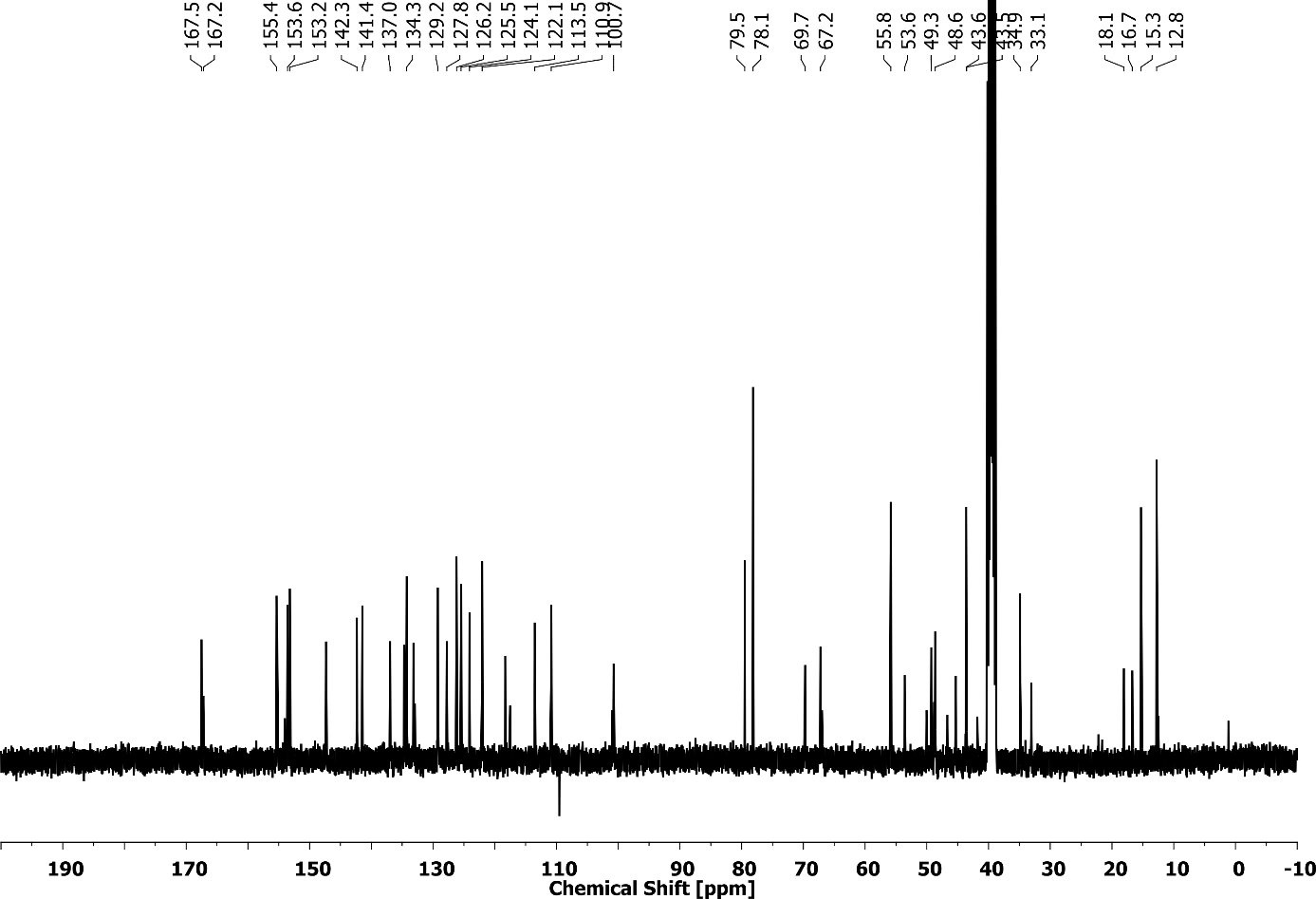


Fig. S4: ^13^C NMR spectrum (DMSO-*d*_6_, 101 MHz, 298 K) of compound 7.


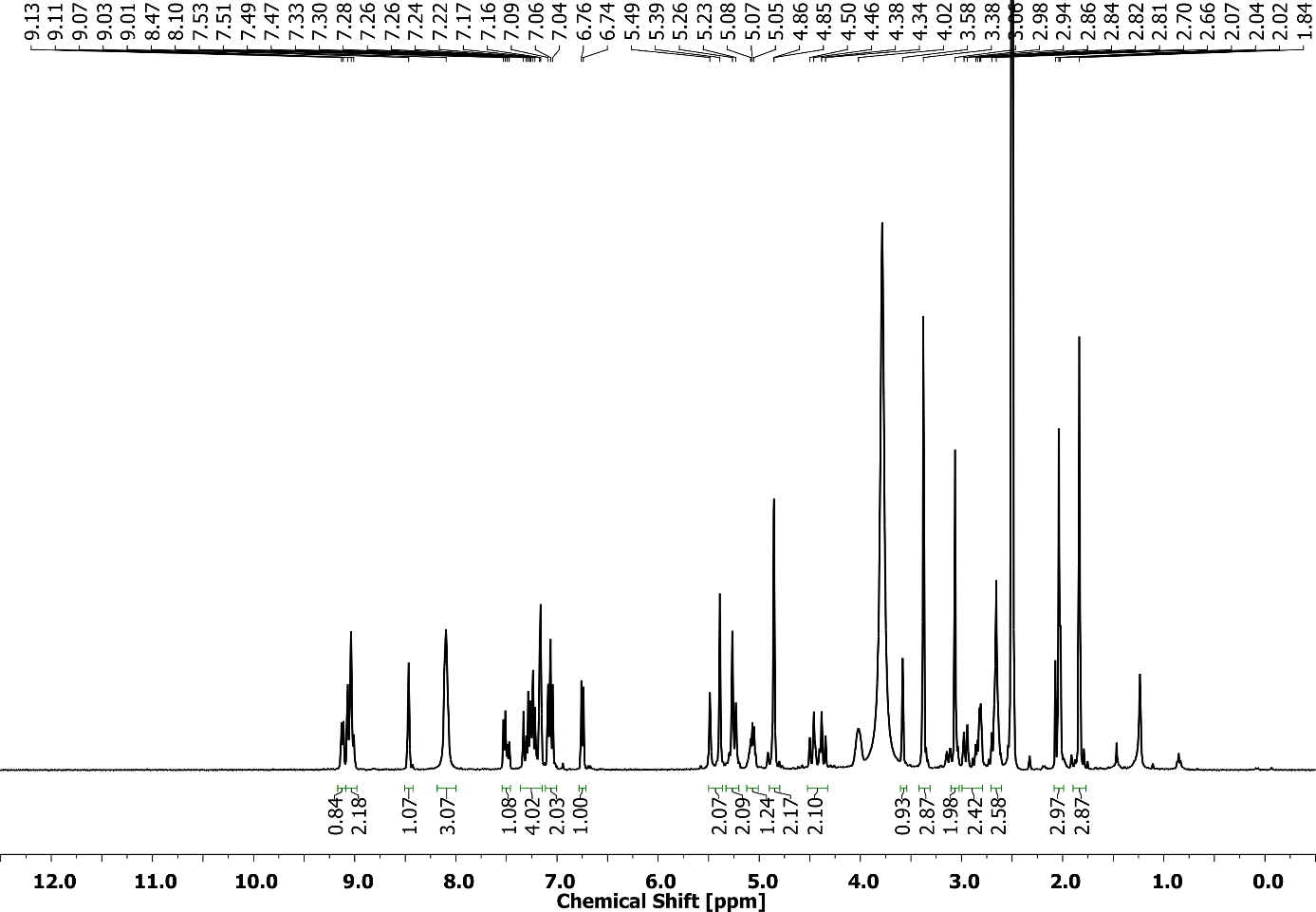


Fig. S5: ^1^H NMR spectrum (DMSO-*d*_6_, 400 MHz, 298 K) of compound 9.


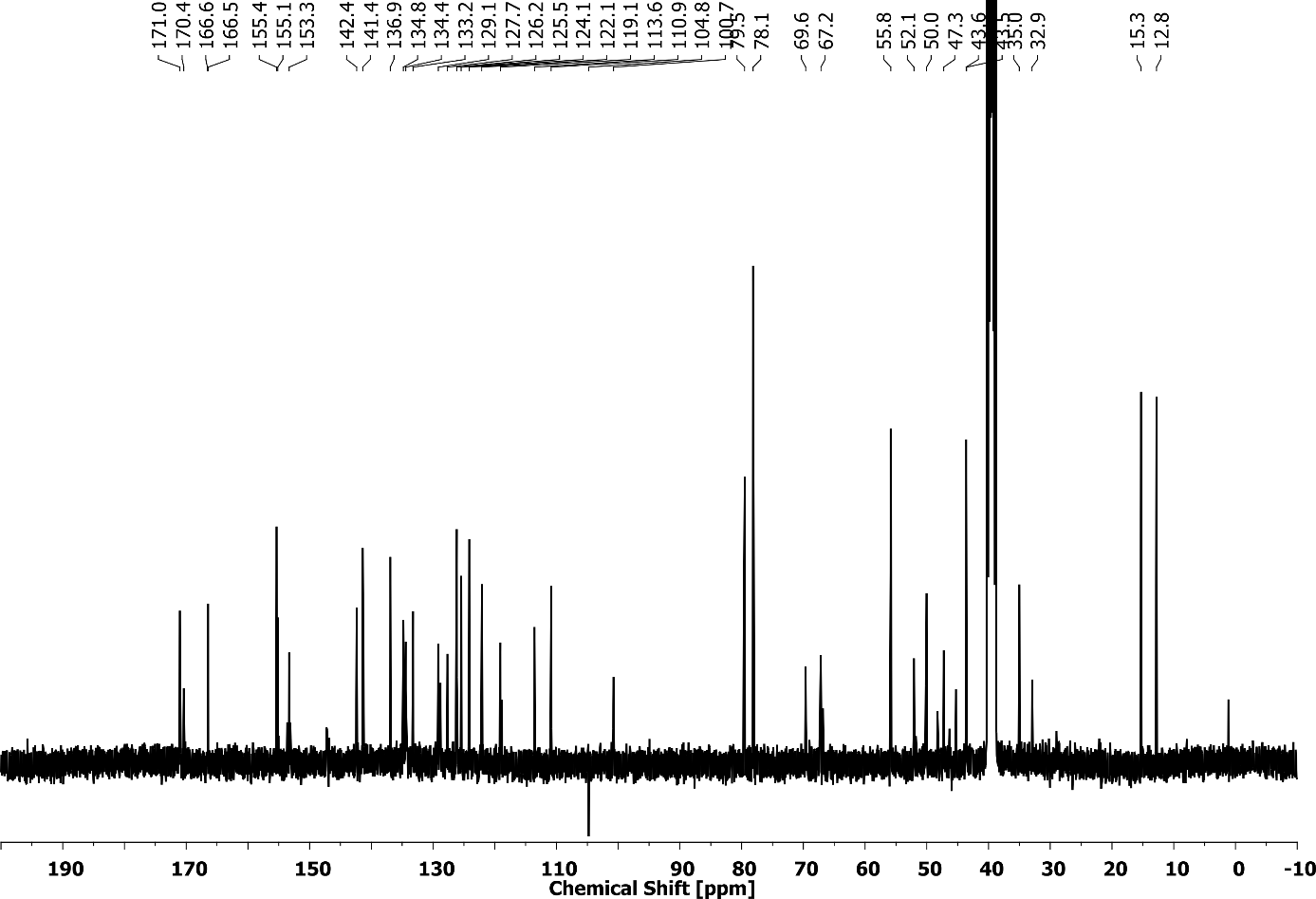


Fig. S6: ^13^C NMR spectrum (DMSO-*d*_6_, 101 MHz, 298 K) of compound 9.


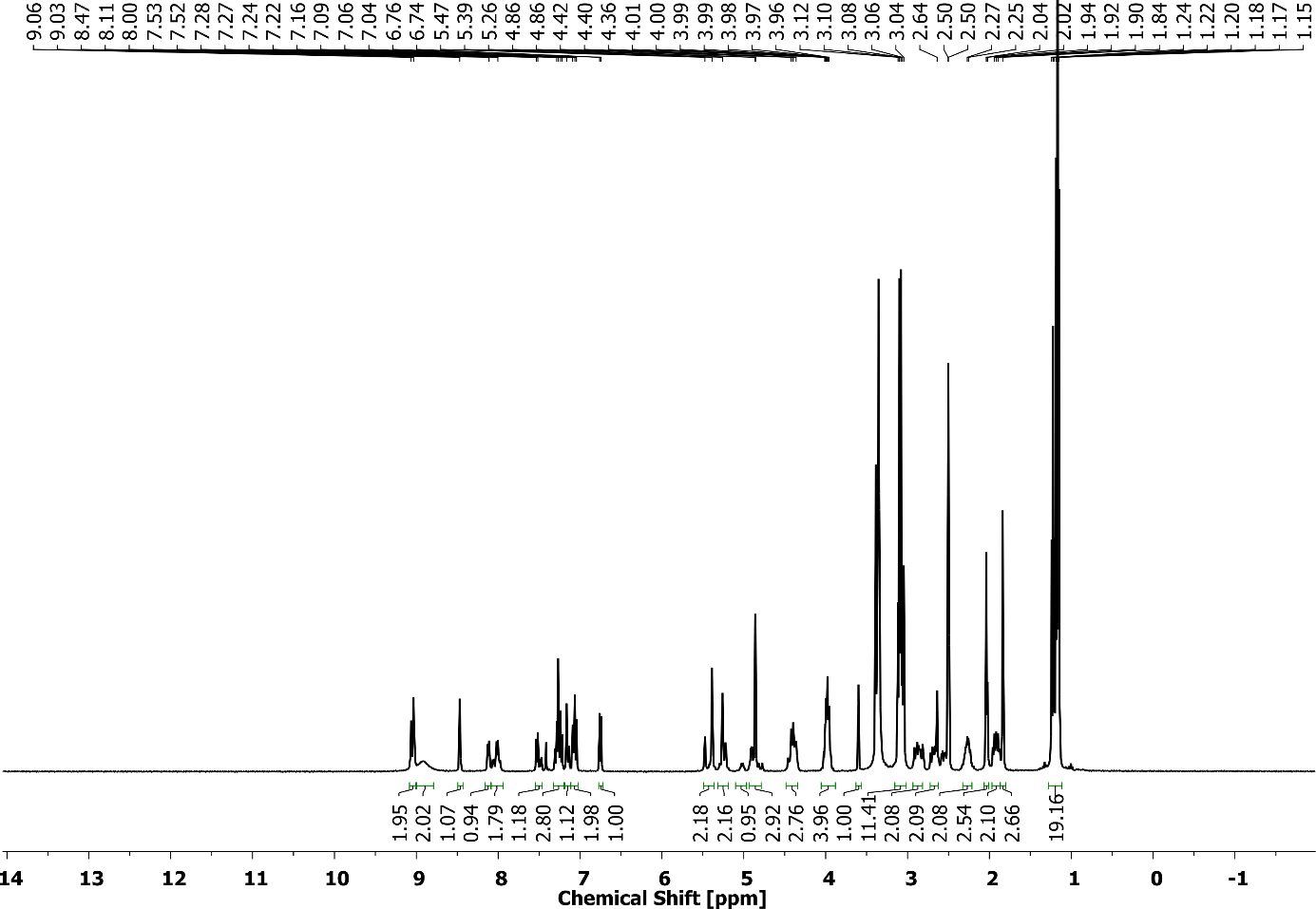


Fig. S7: ^1^H NMR spectrum (DMSO-*d*_6_, 400 MHz, 298 K) of compound 10.


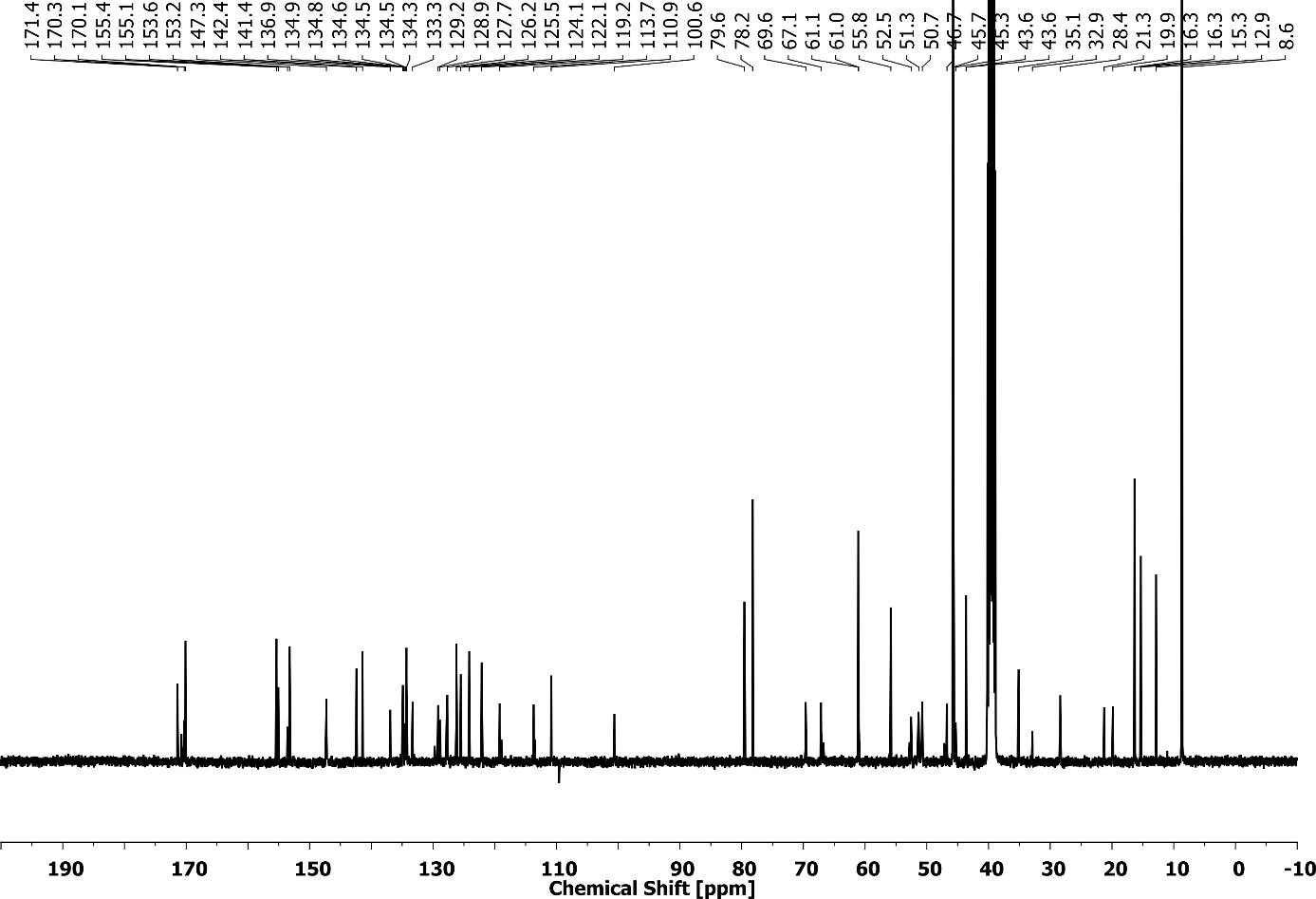


Fig. S8: ^13^C NMR spectrum (DMSO-*d*_6_, 101 MHz, 298 K) of compound 10.


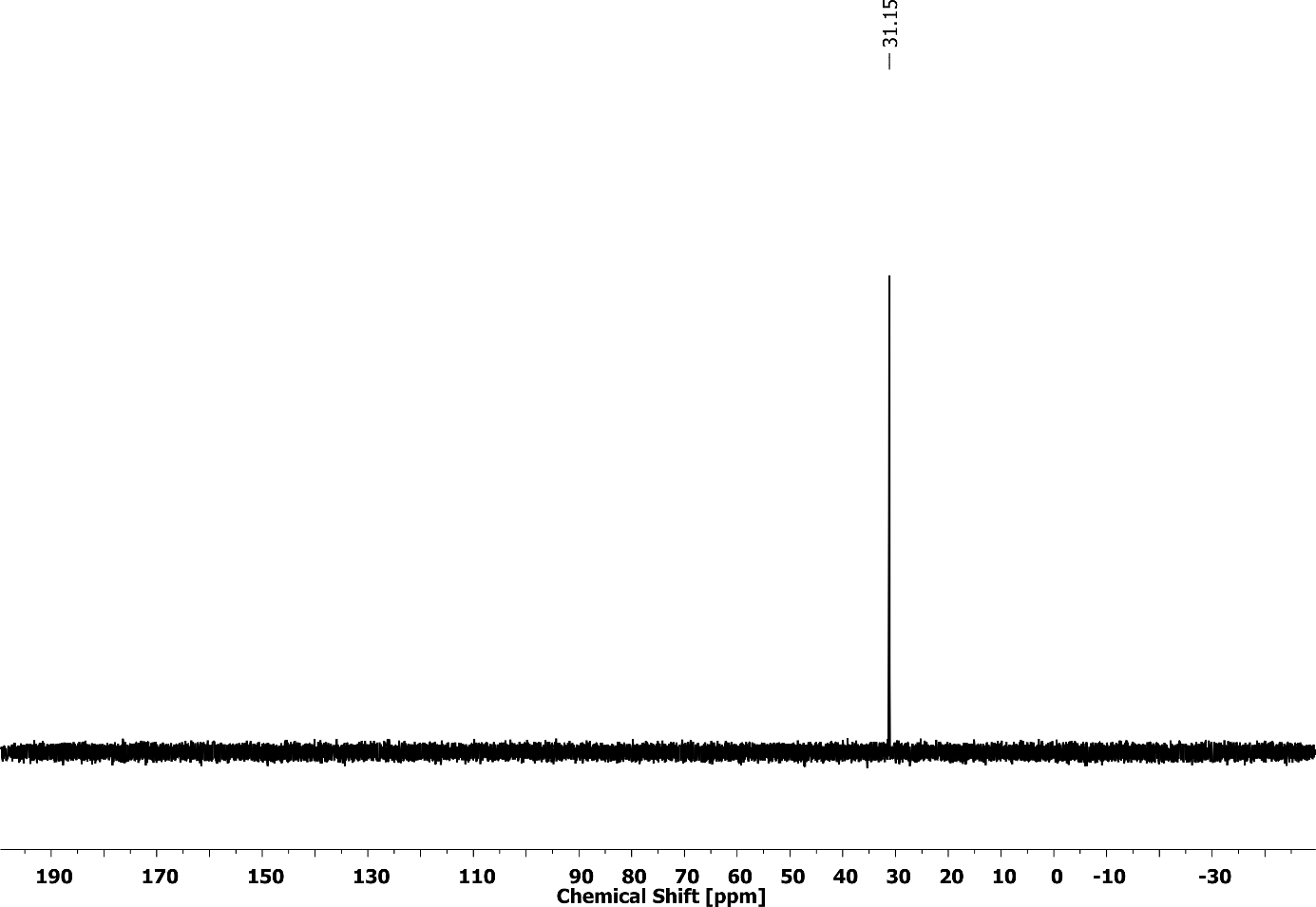


Fig. S9: ^31^P NMR spectrum (DMSO-*d*_6_, 162 MHz, 298 K) of compound 10.


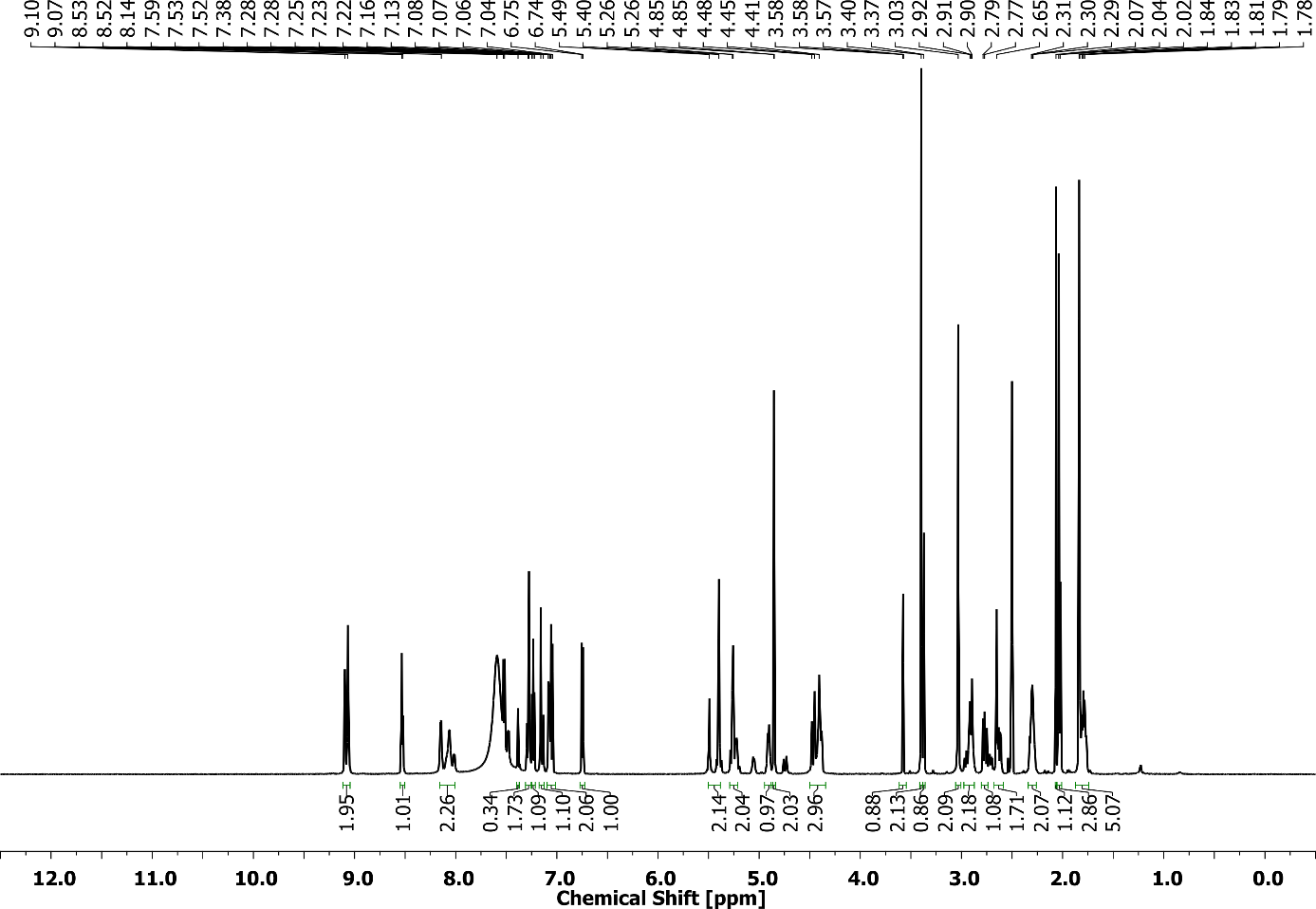


Fig. S10: ^1^H NMR spectrum (DMSO-*d*_6_, 400 MHz, 298 K) of compound 11.


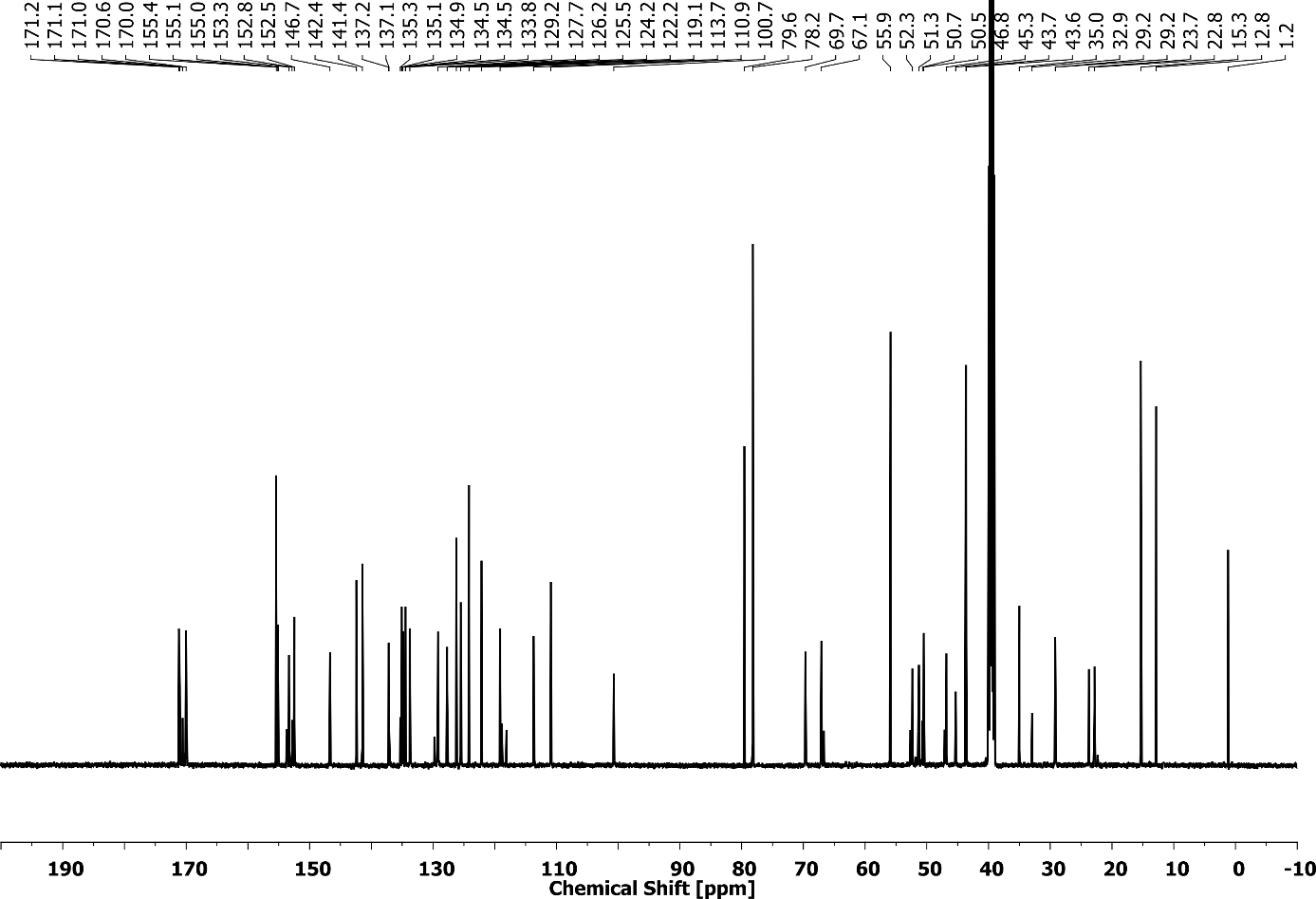


Fig. S11: ^13^C NMR spectrum (DMSO-*d*_6_, 101 MHz, 298 K) of compound 11.


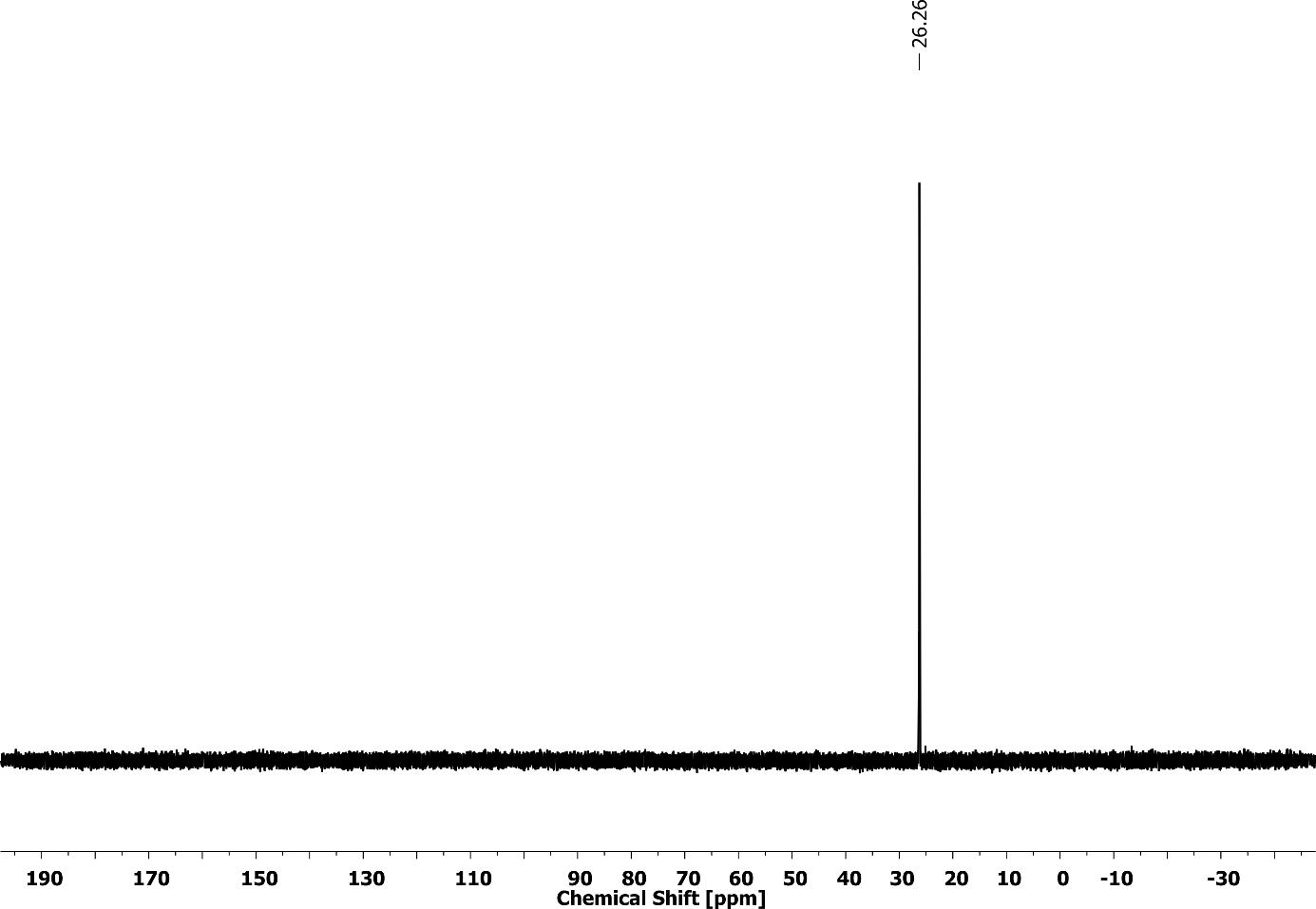


Fig. S12: ^31^P NMR spectrum (DMSO-*d*_6_, 162 MHz, 298 K) of compound 11.


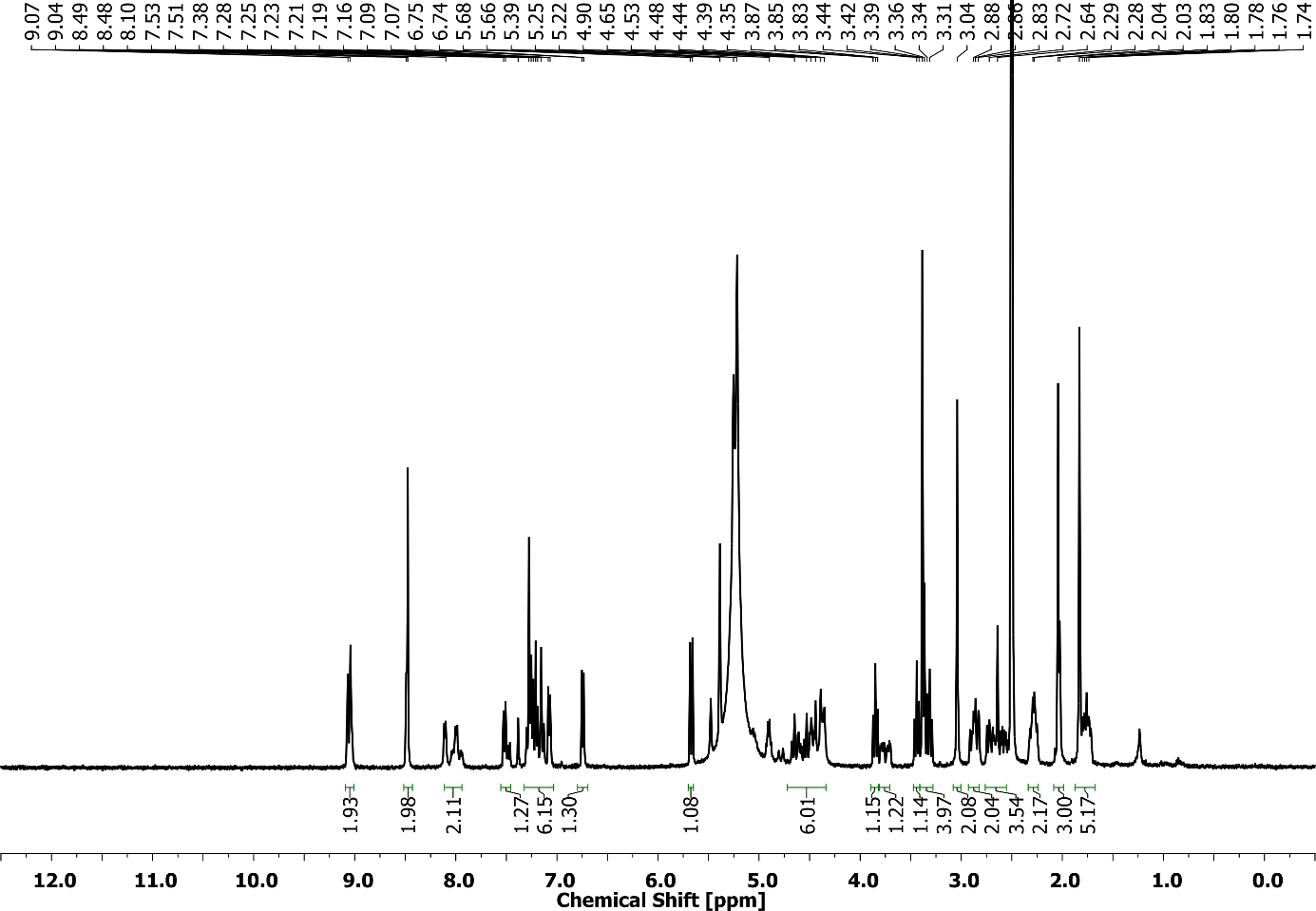


Fig. S13: ^1^H NMR spectrum (DMSO-*d*_6_, 400 MHz, 298 K) of compound 2.


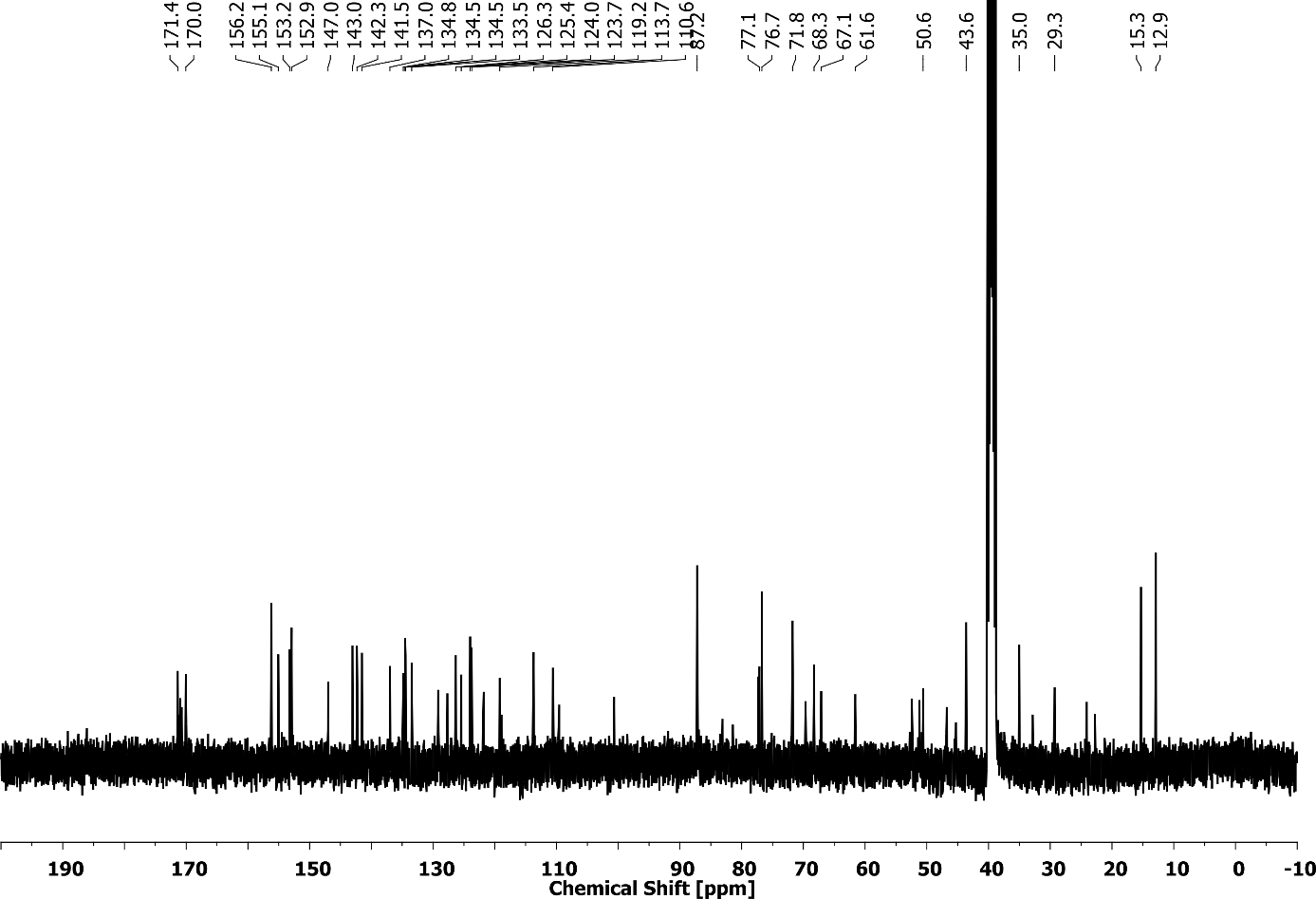


Fig. S14: ^13^C NMR spectrum (DMSO-*d*_6_, 101 MHz, 298 K) of compound 2.


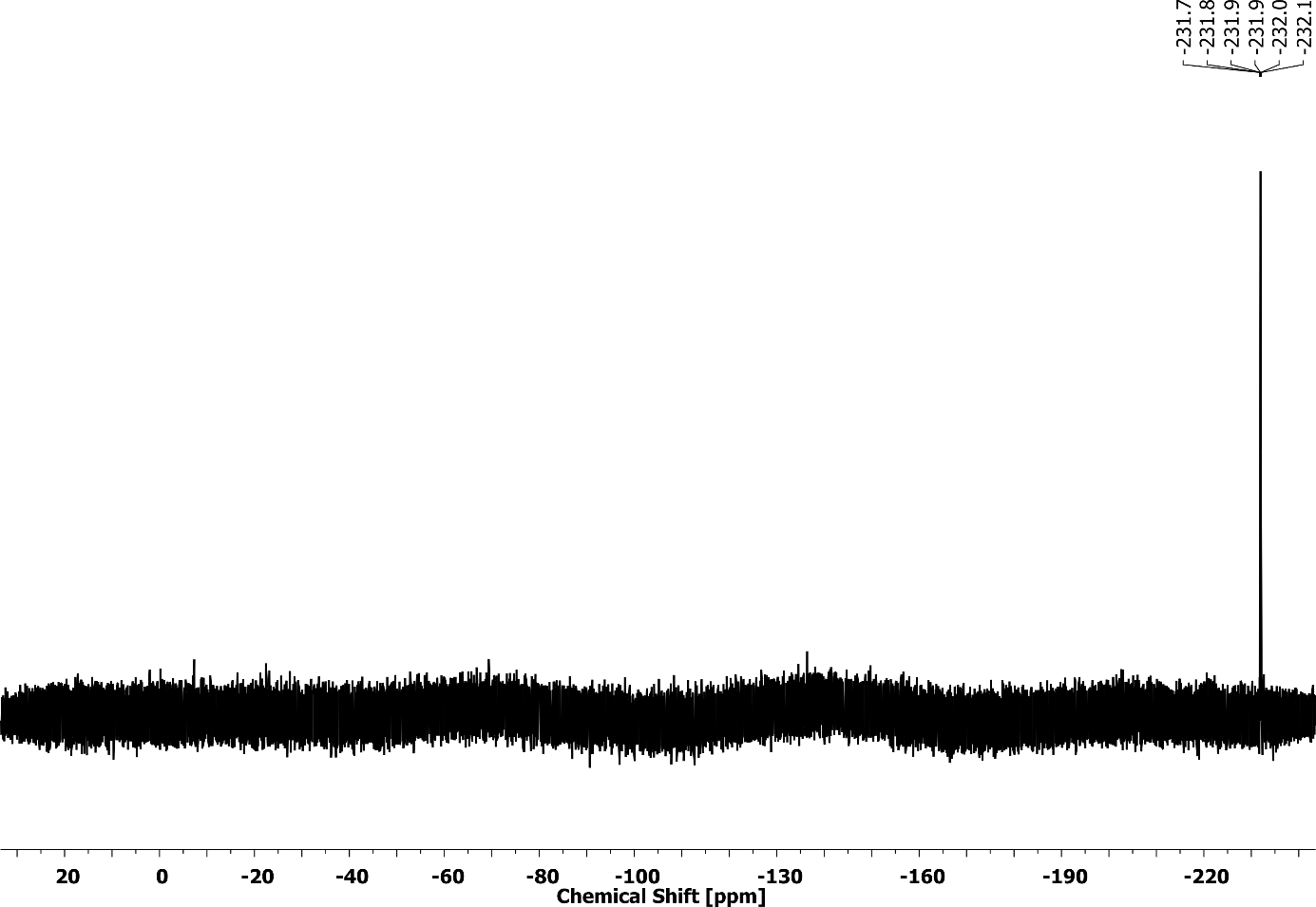


Fig. S15: ^19^F NMR spectrum (DMSO-*d*_6_, 376 MHz, 298 K) of compound 2.


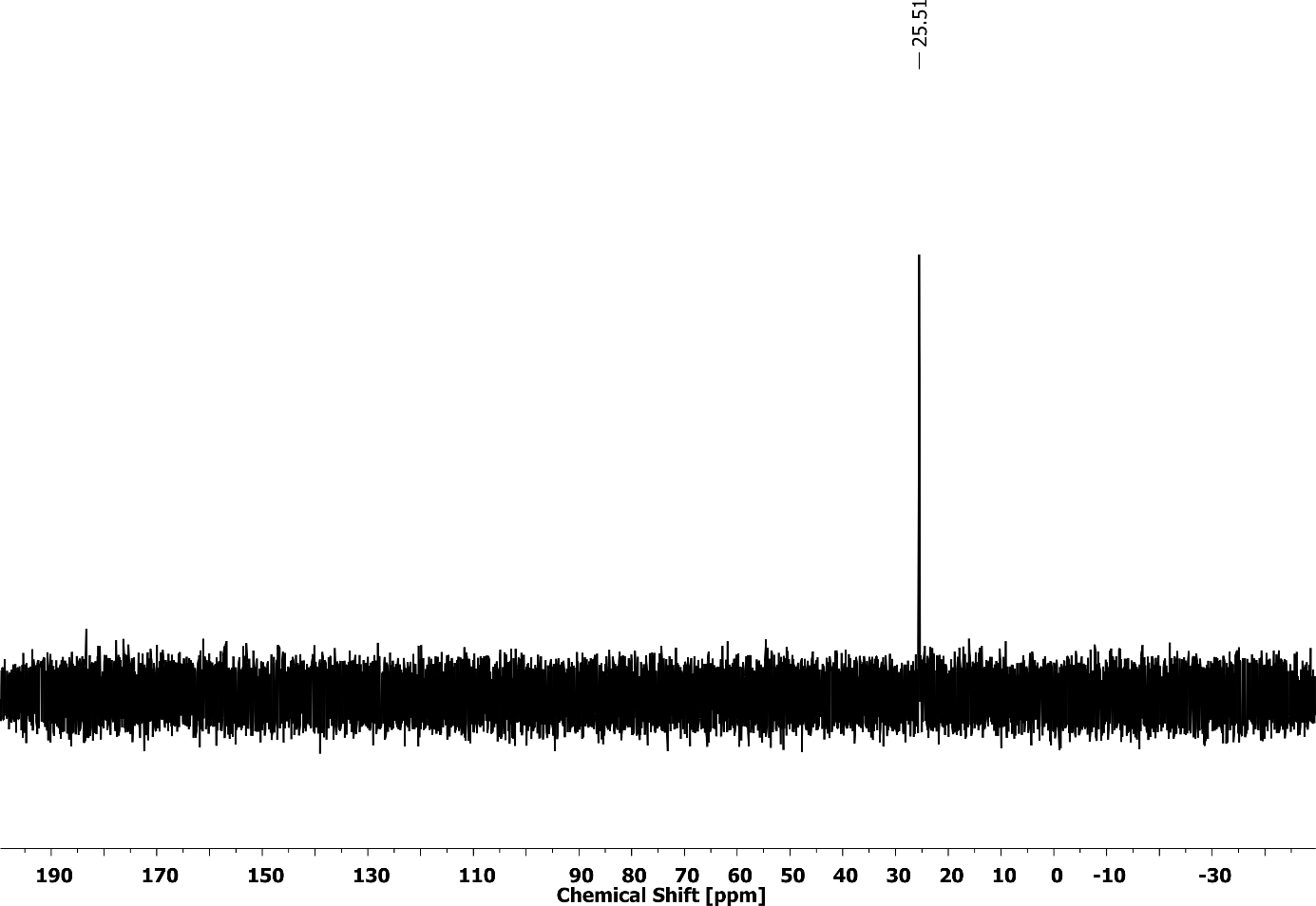


Fig. S16: ^31^P NMR spectrum (DMSO-*d*_6_, 162 MHz, 298 K) of compound 2.


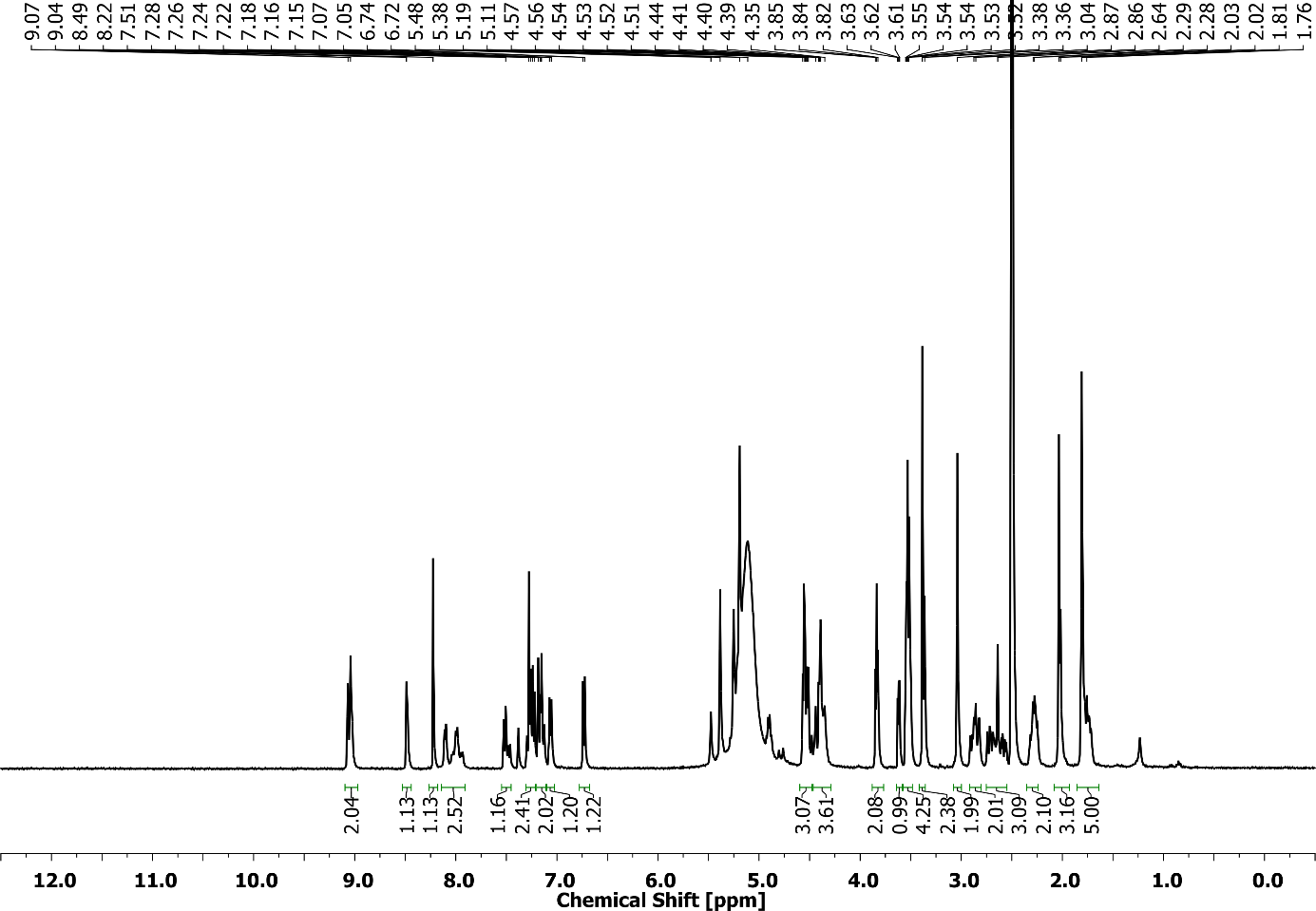


Fig. S17: ^1^H NMR spectrum (DMSO-*d*_6_, 400 MHz, 298 K) of compound 3.


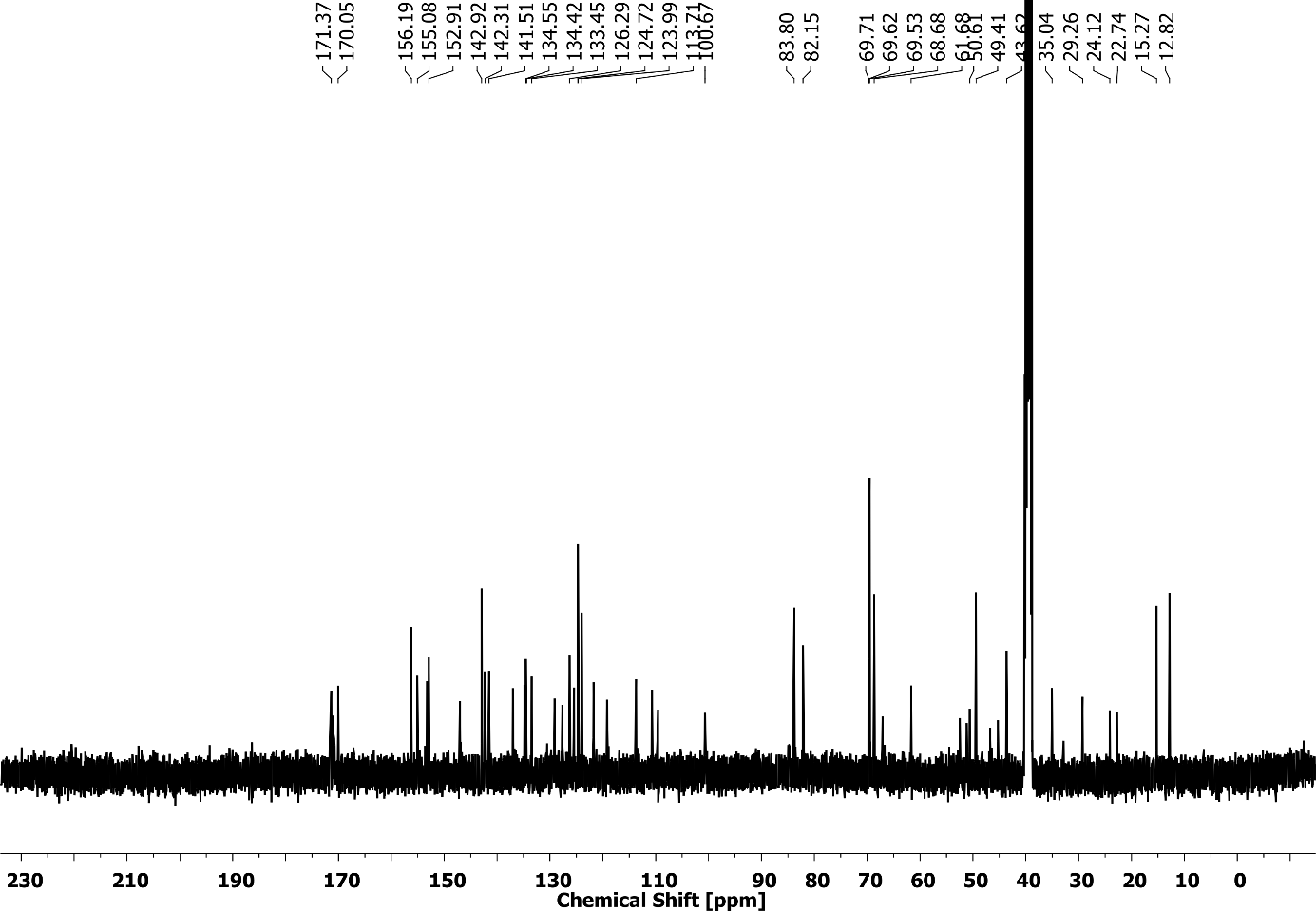


Fig. S18: ^13^C NMR spectrum (DMSO-*d*_6_, 101 MHz, 298 K) of compound 3.


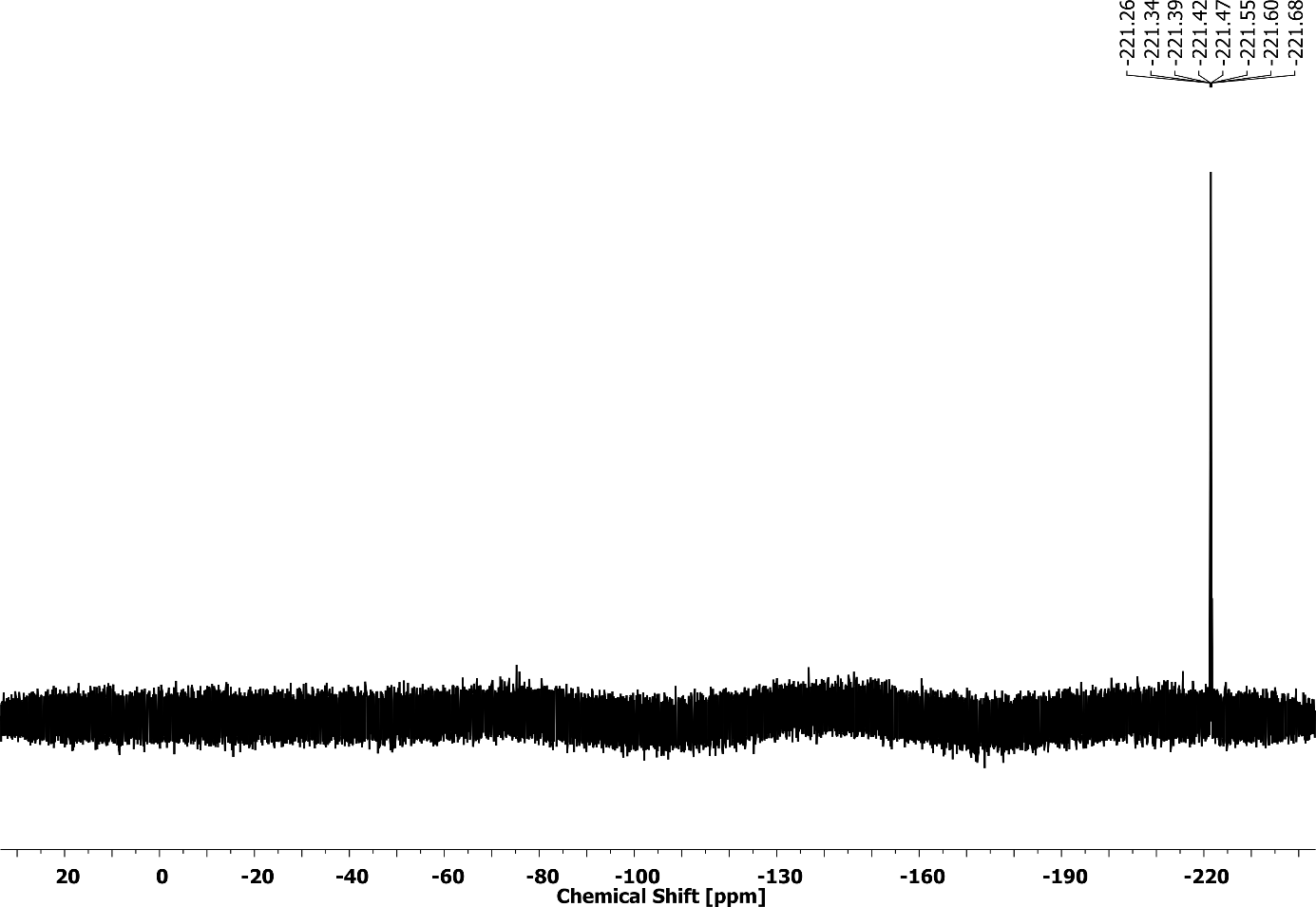


Fig. S19: ^19^F NMR spectrum (DMSO-*d*_6_, 376 MHz, 298 K) of compound 3.


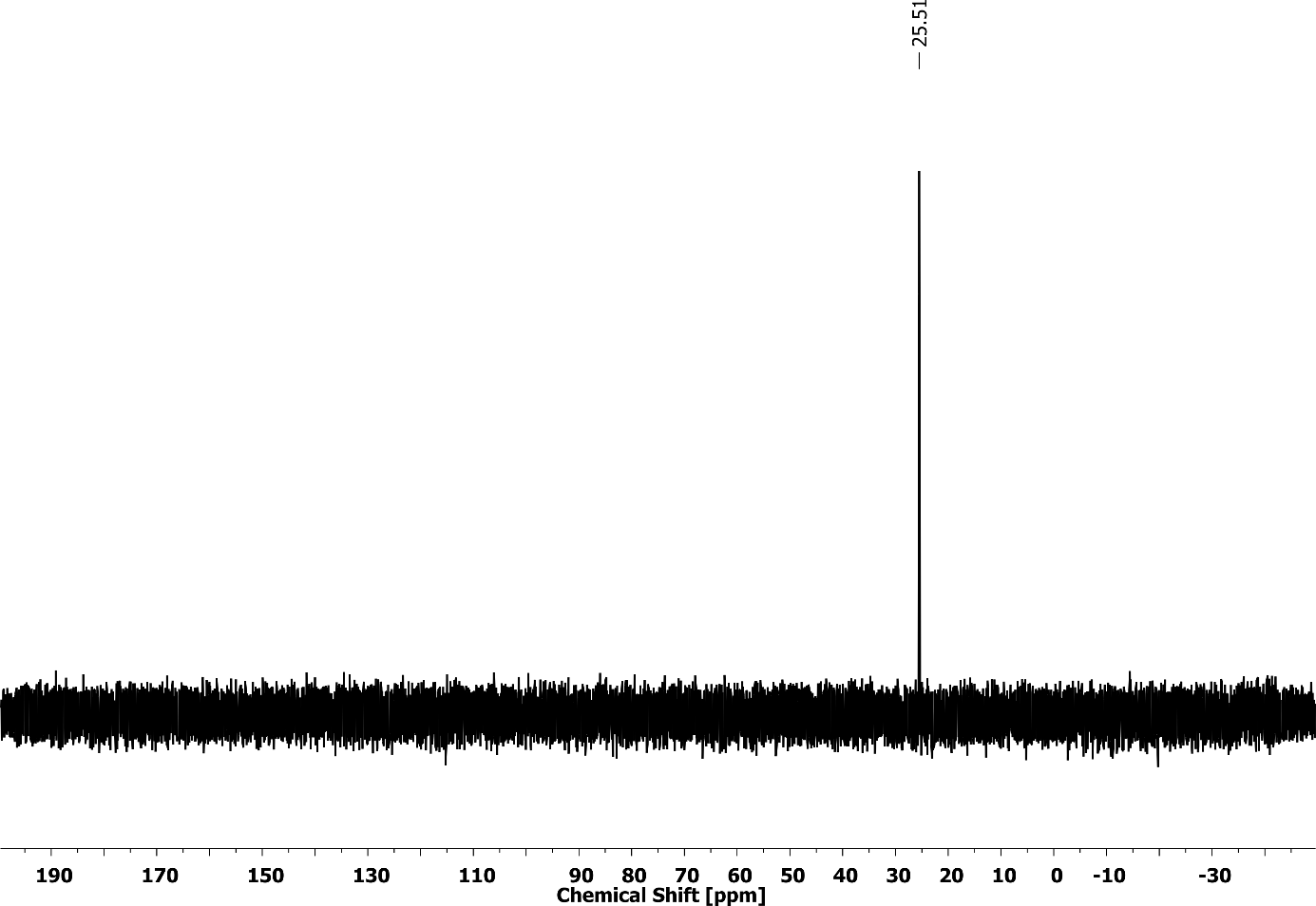


Fig. S20: ^31^P NMR spectrum (DMSO-*d*_6_, 162 MHz, 298 K) of compound 3.


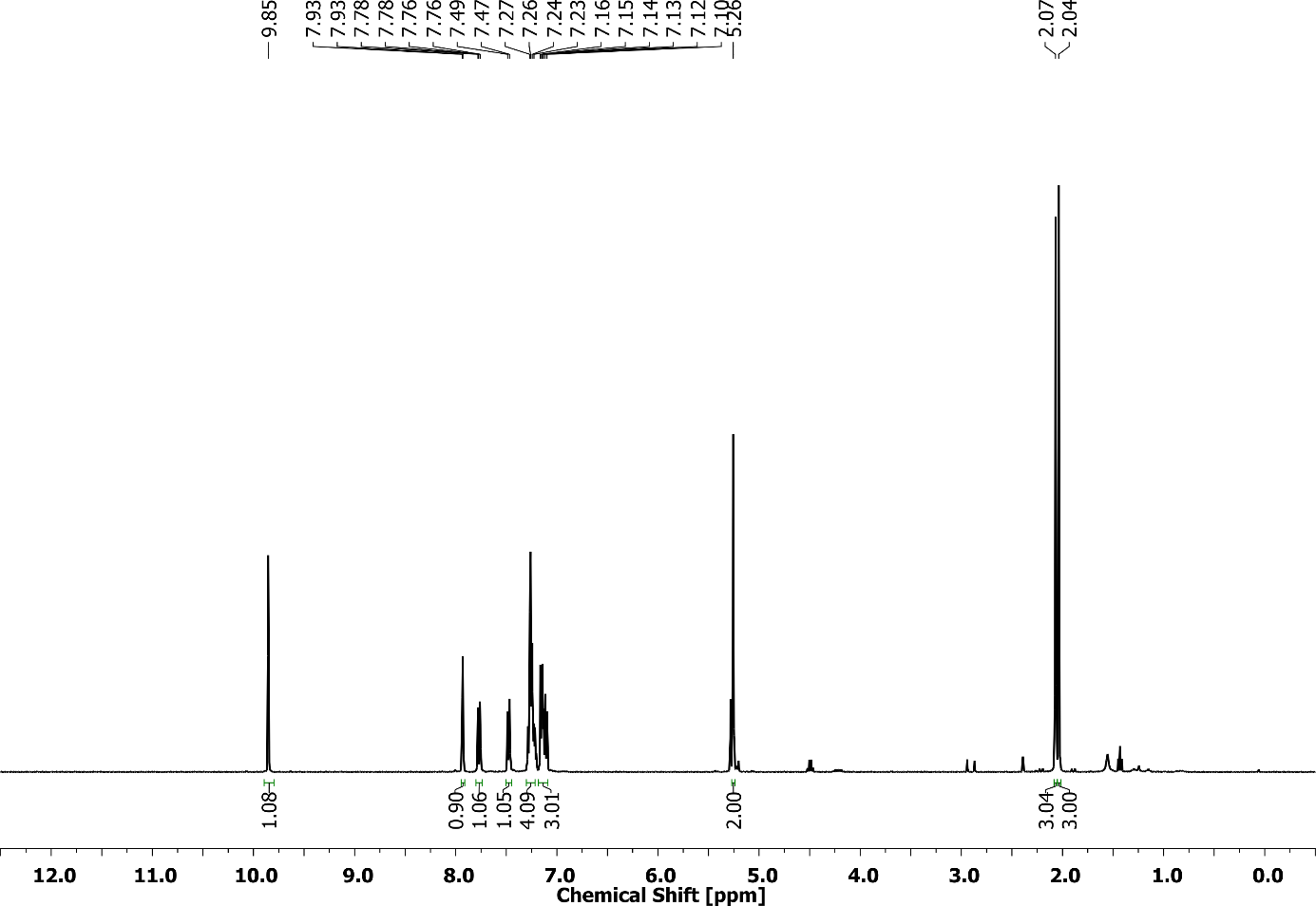


Fig. S21: ^1^H NMR spectrum (CDCl_3_, 400 MHz, 298 K) of compound 24.


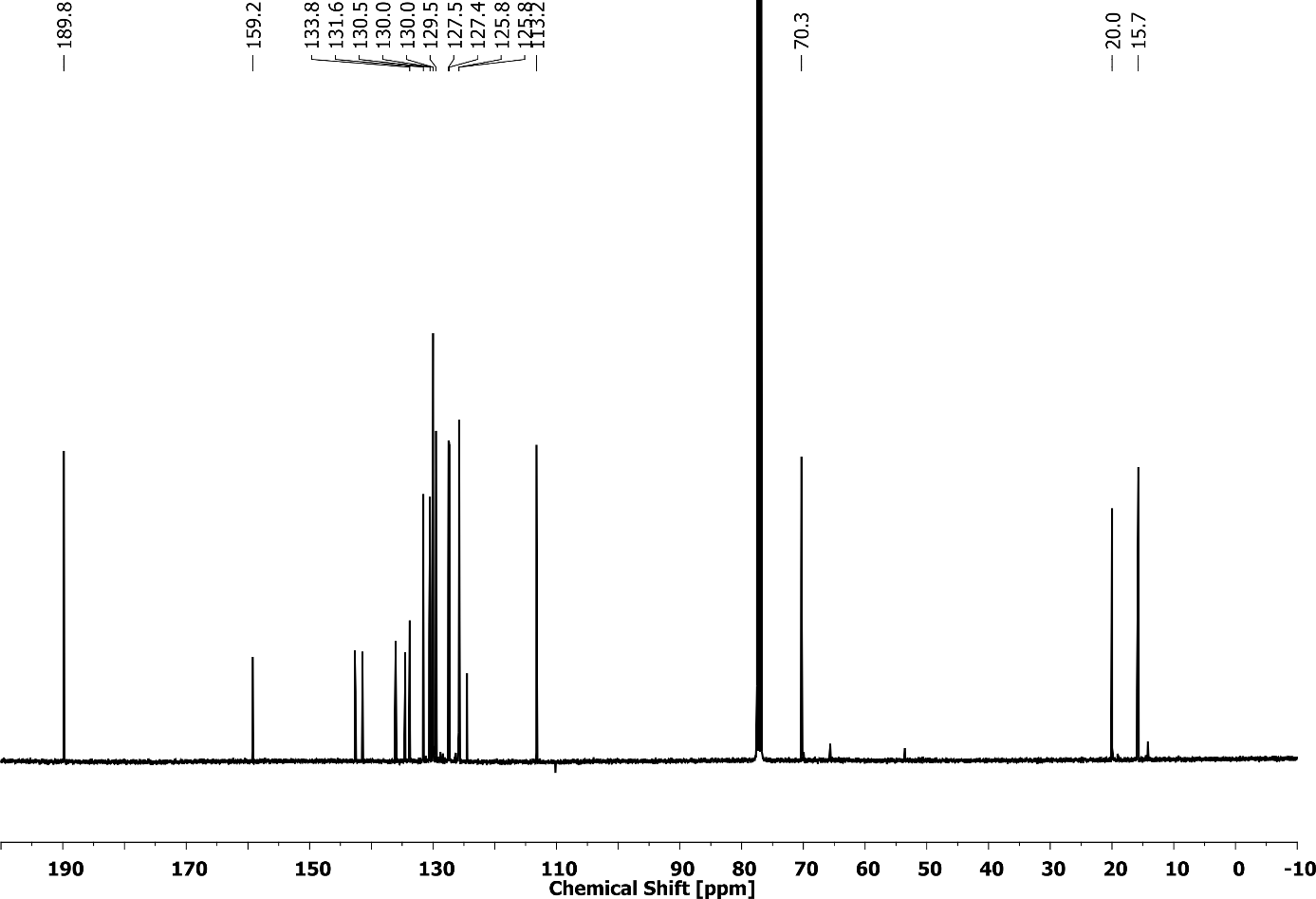


Fig. S22: ^13^C NMR spectrum (CDCl_3_, 101 MHz, 298 K) of compound 24.


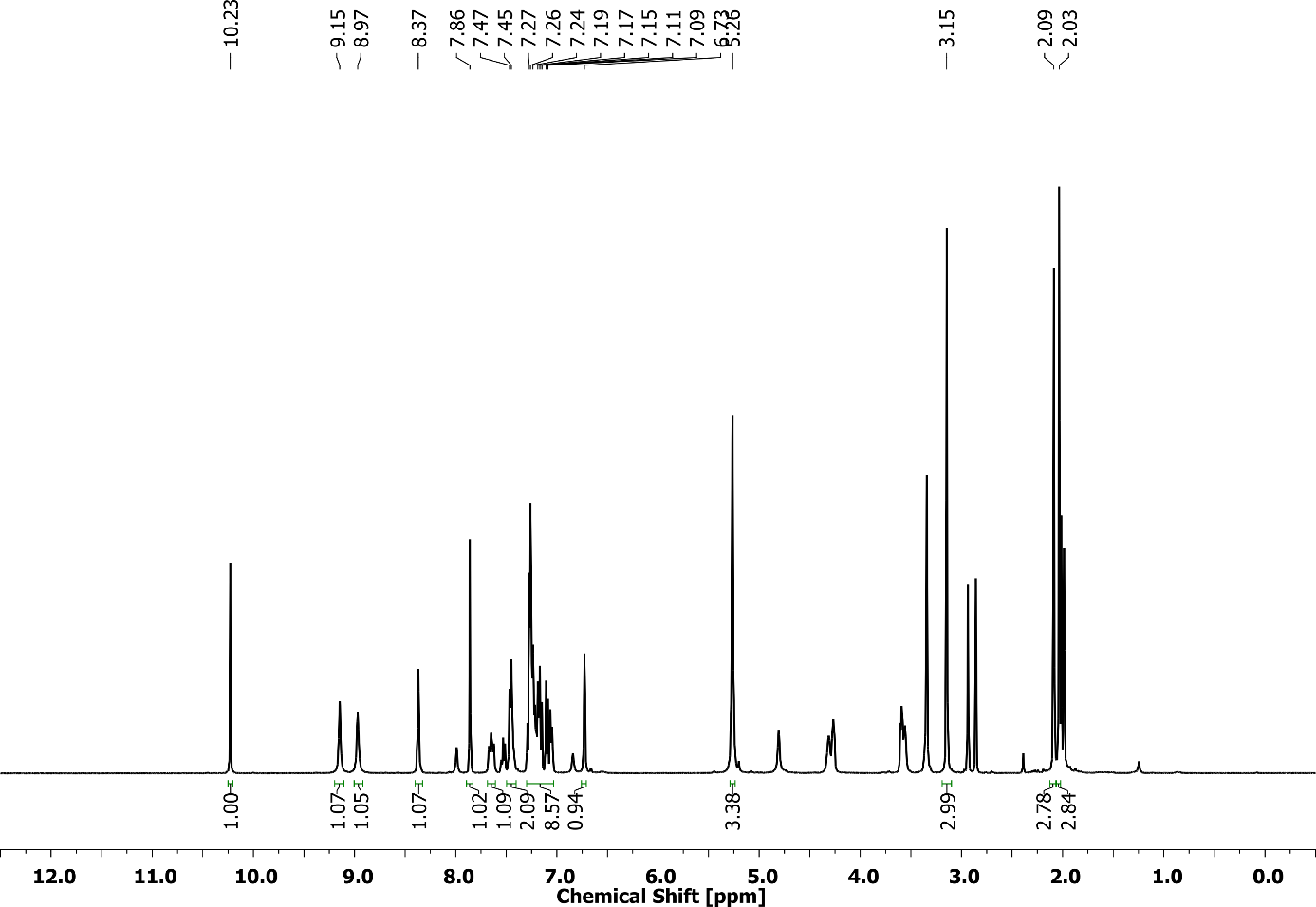


Fig. S23: ^1^H NMR spectrum (CDCl_3_, 400 MHz, 298 K) of compound 25.


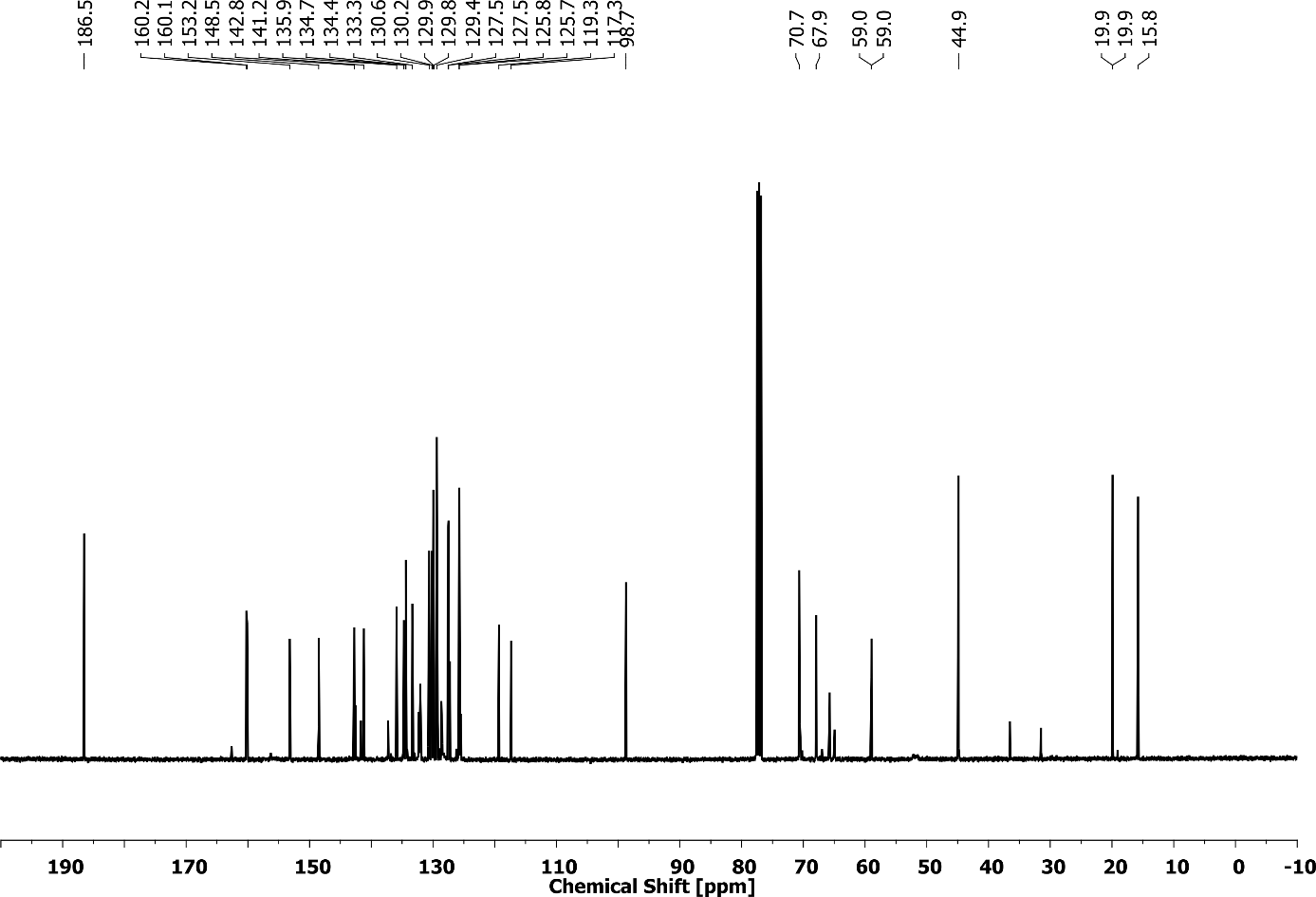


Fig. S24: ^13^C NMR spectrum (CDCl_3_, 101 MHz, 298 K) of compound 25.


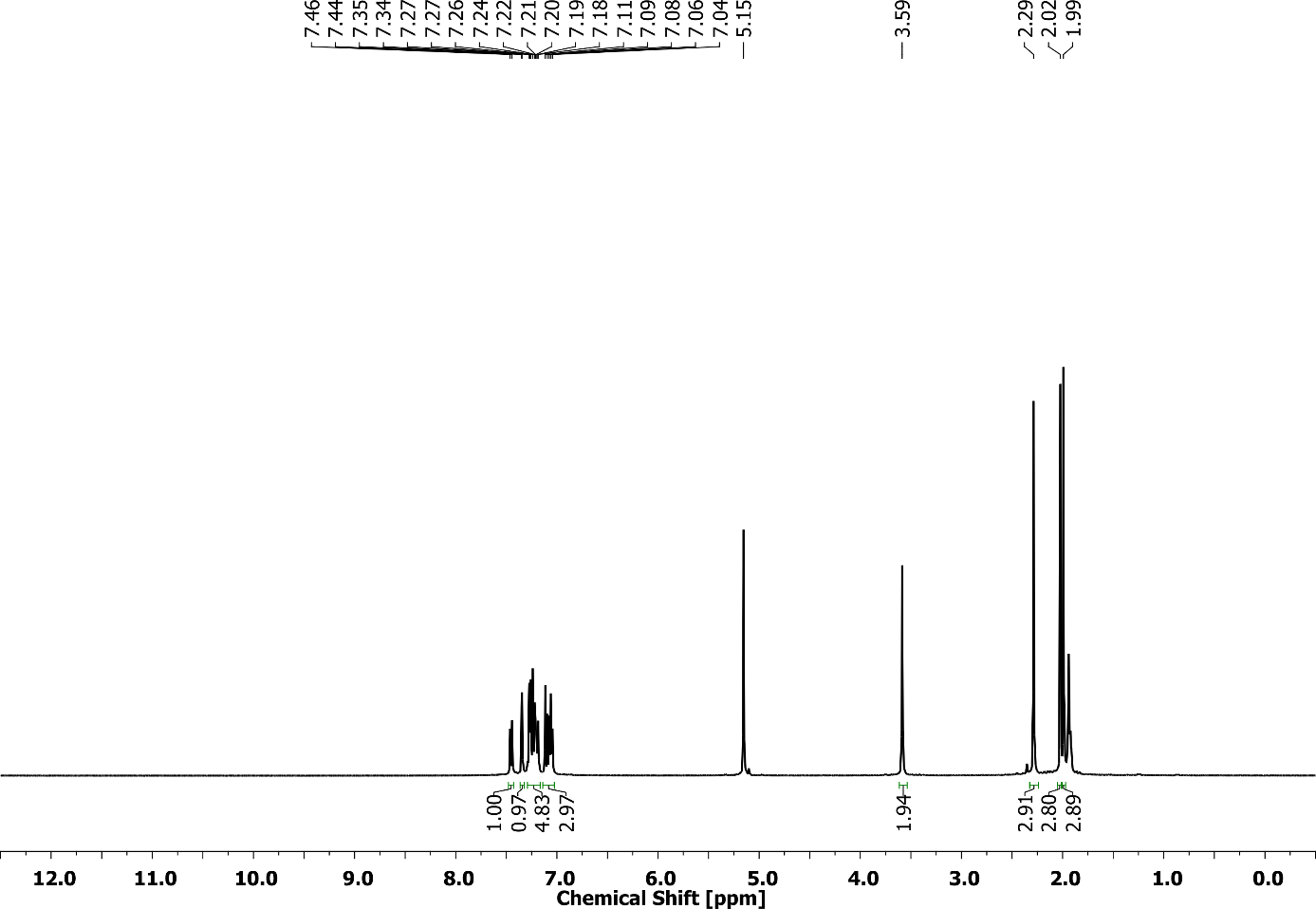


Fig. S25: ^1^H NMR spectrum (MeCN-*d*_3_, 400 MHz, 298 K) of compound 26.


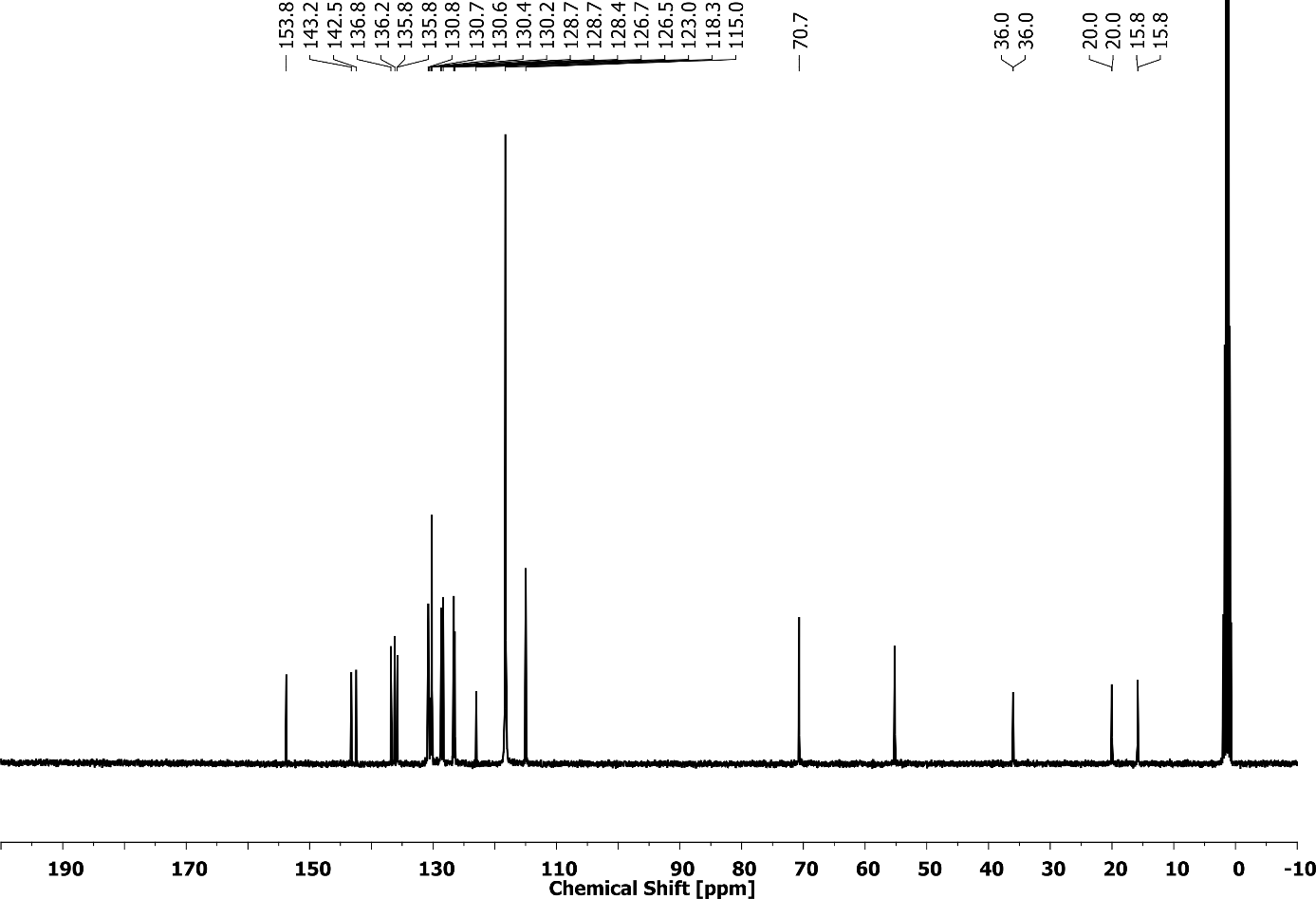


Fig. S26: ^13^C NMR spectrum (MeCN-*d*_3_, 101 MHz, 298 K) of compound 26.


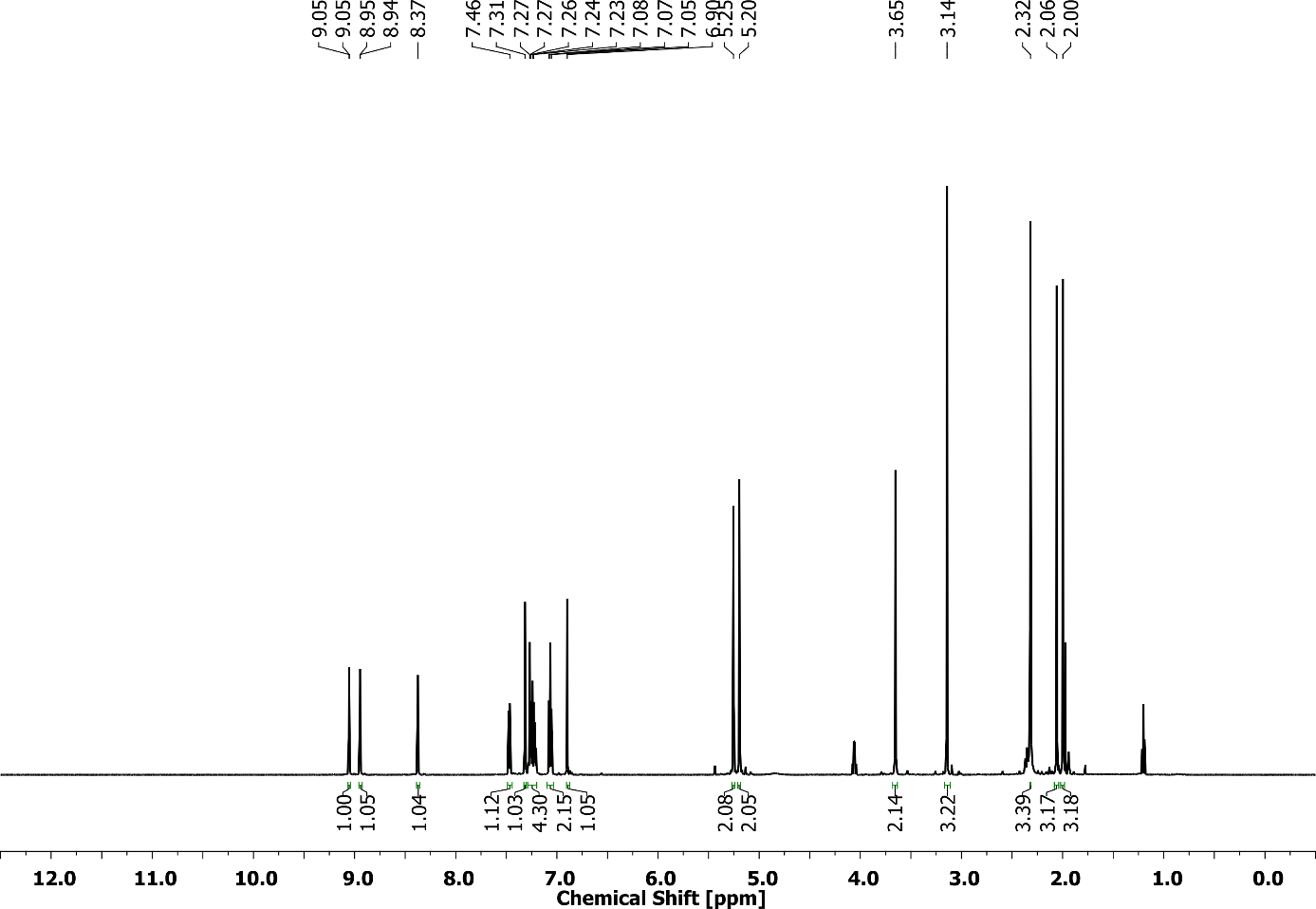


Fig. S27: ^1^H NMR spectrum (MeCN-*d*_6_, 400 MHz, 298 K) of compound 27.


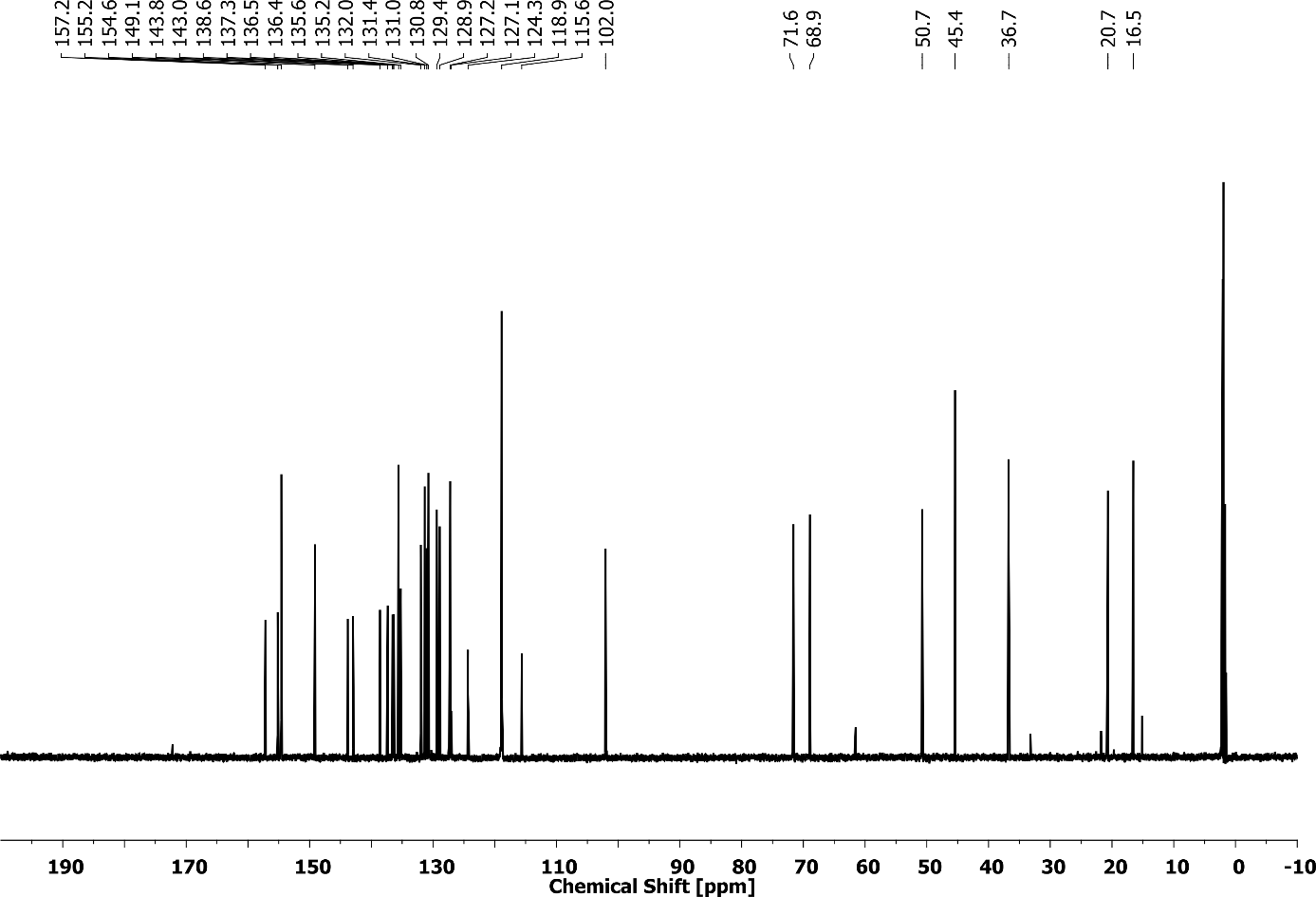


Fig. S28: ^13^C NMR spectrum (MeCN-*d*_3_, 101 MHz, 298 K) of compound 27.


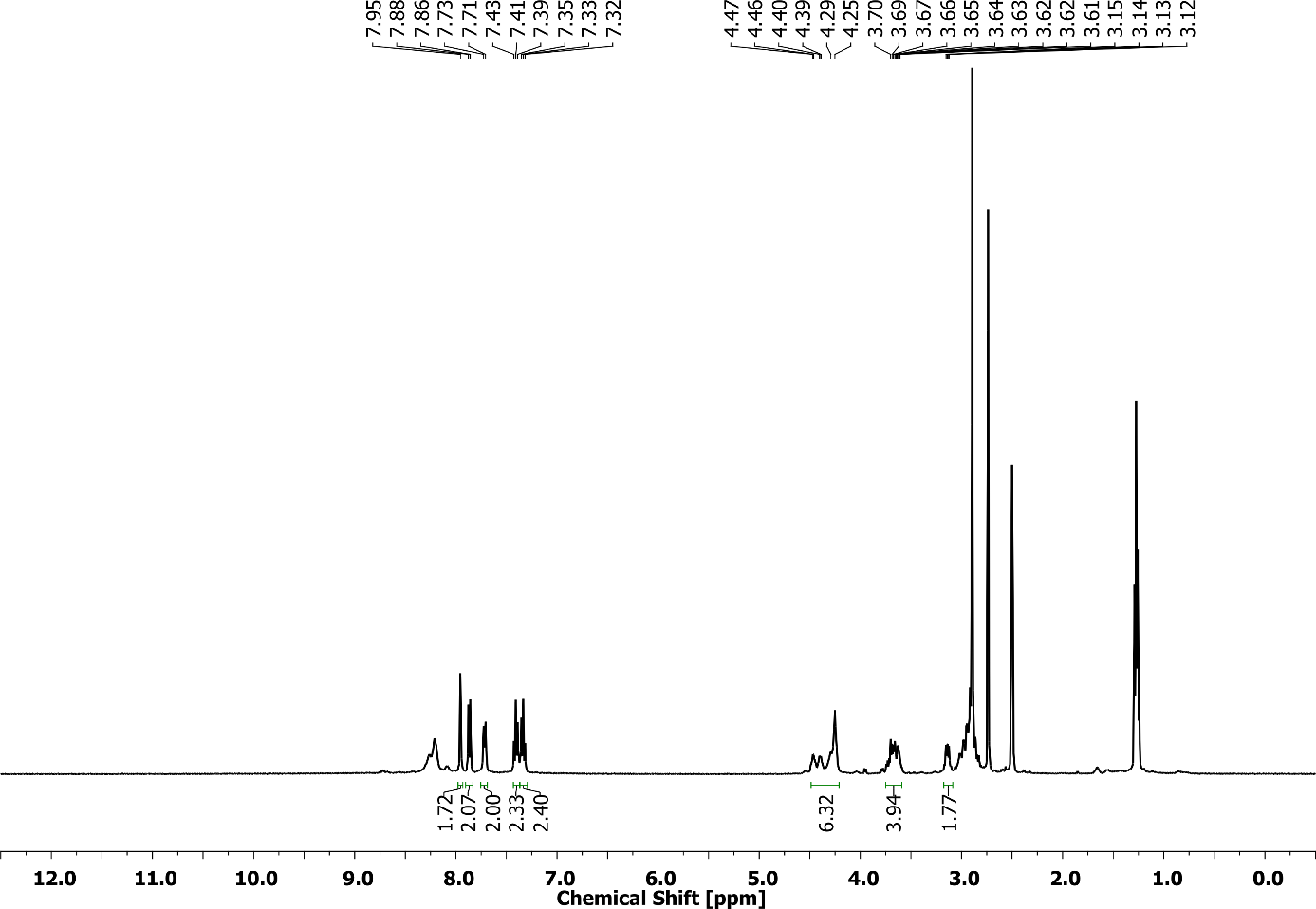


Fig. S29: ^1^H NMR spectrum (DMSO-*d*_6_, 400 MHz, 298 K) of compound 20.


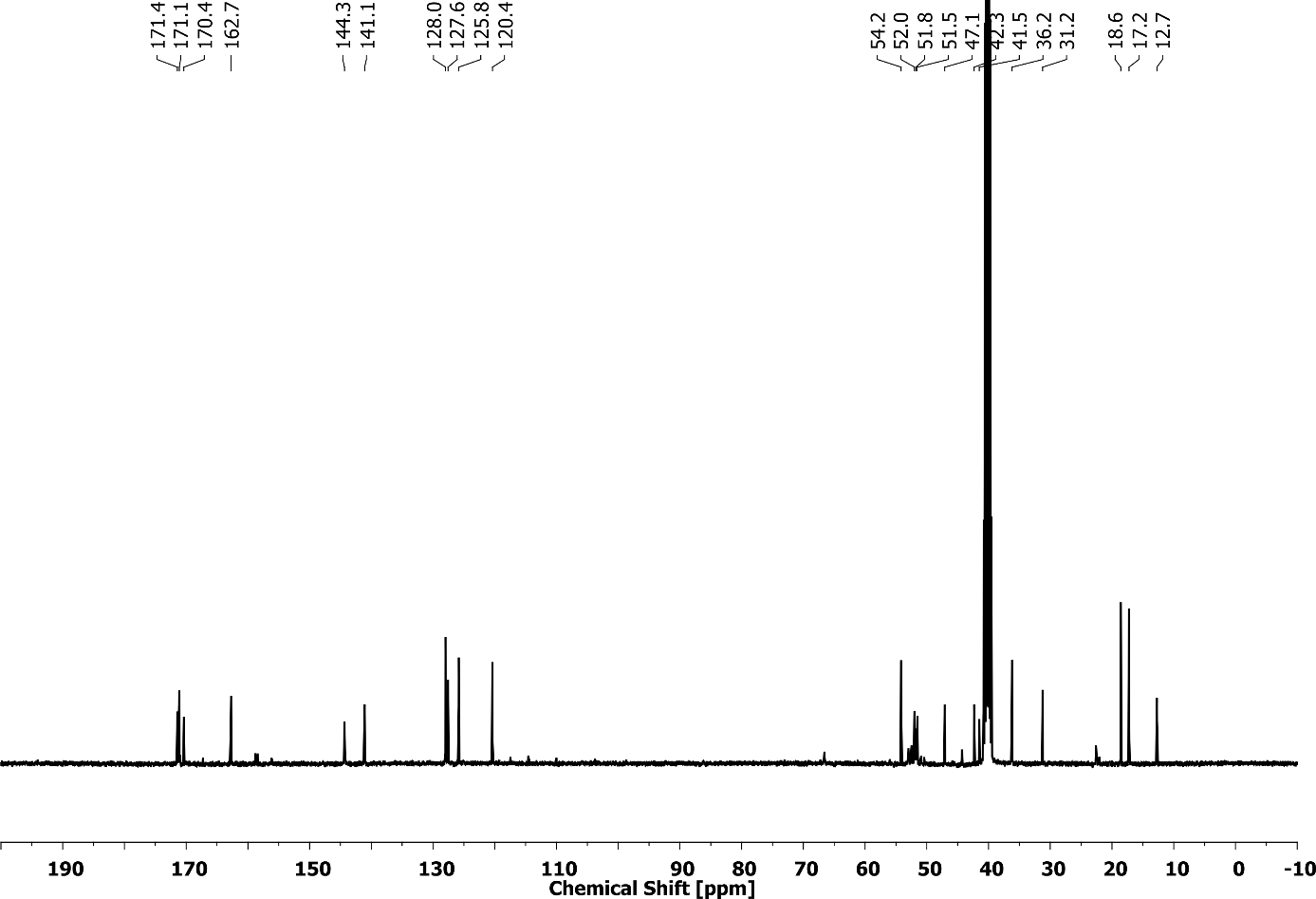


Fig. S30: ^13^C NMR spectrum (DMSO-*d*_6_, 101 MHz, 298 K) of compound 20.


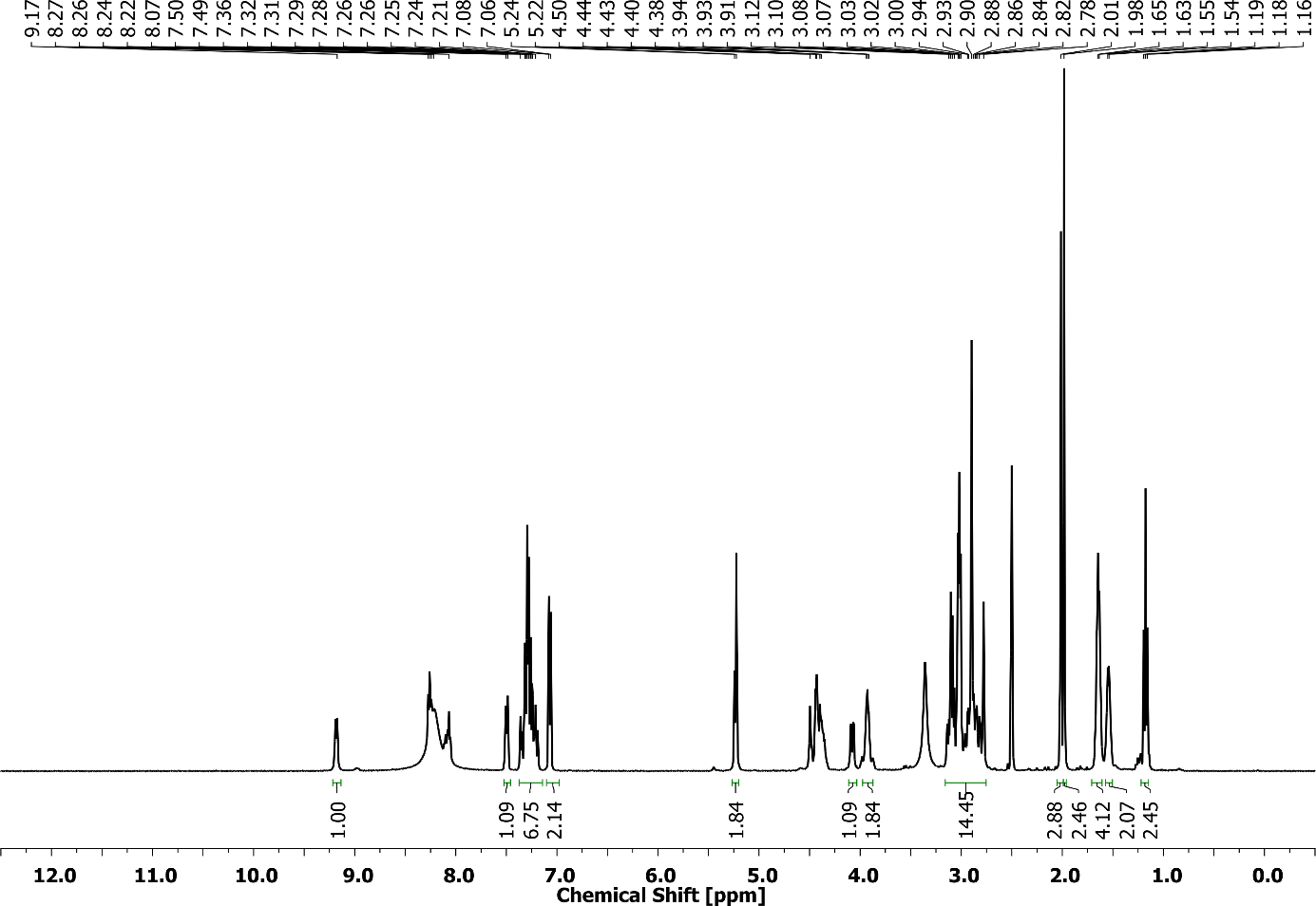


Fig. S31: ^1^H NMR spectrum (DMSO-*d*_6_, 400 MHz, 298 K) of compound 30.


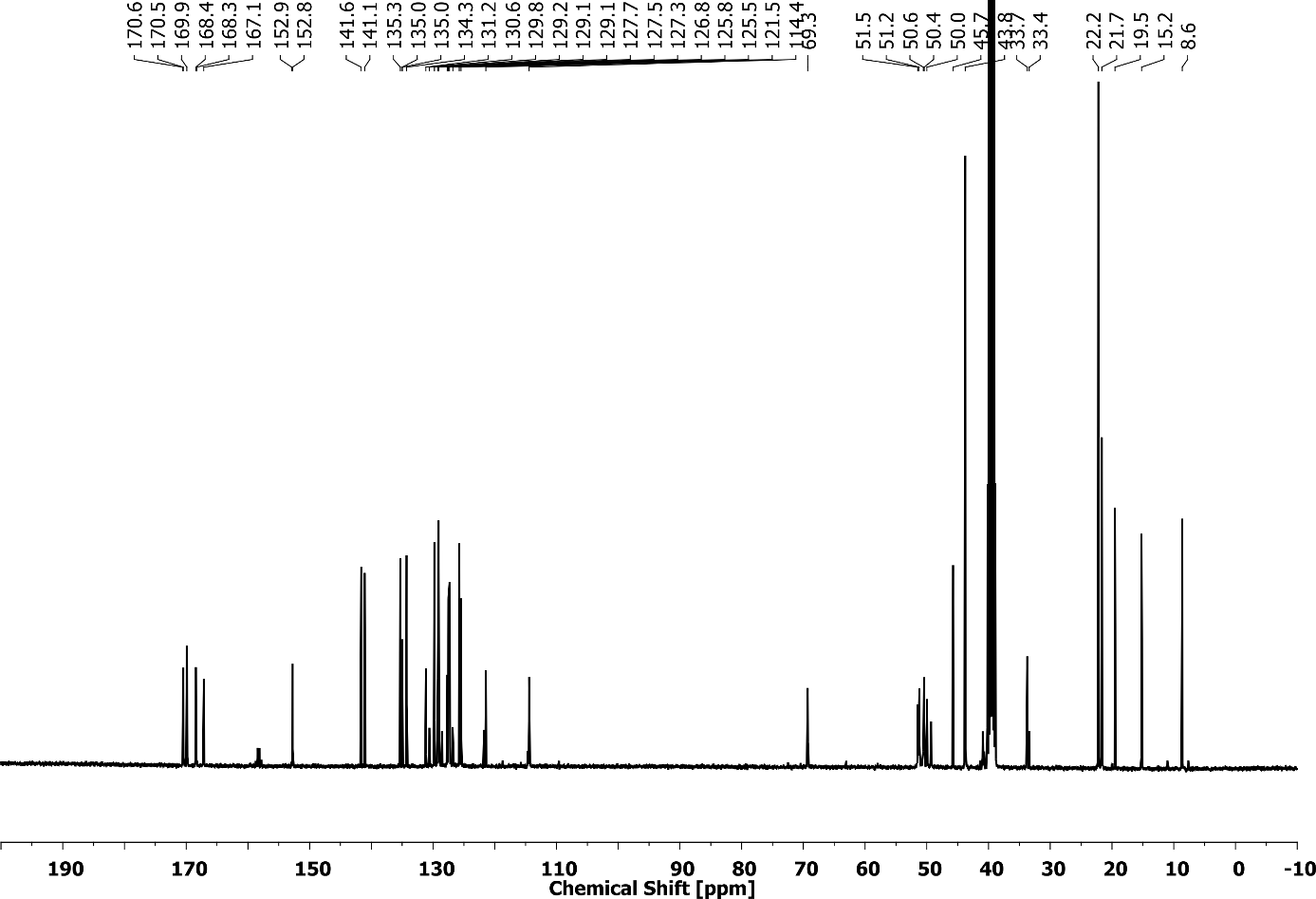


Fig. S32: ^13^C NMR spectrum (DMSO-*d*_6_, 101 MHz, 298 K) of compound 30.


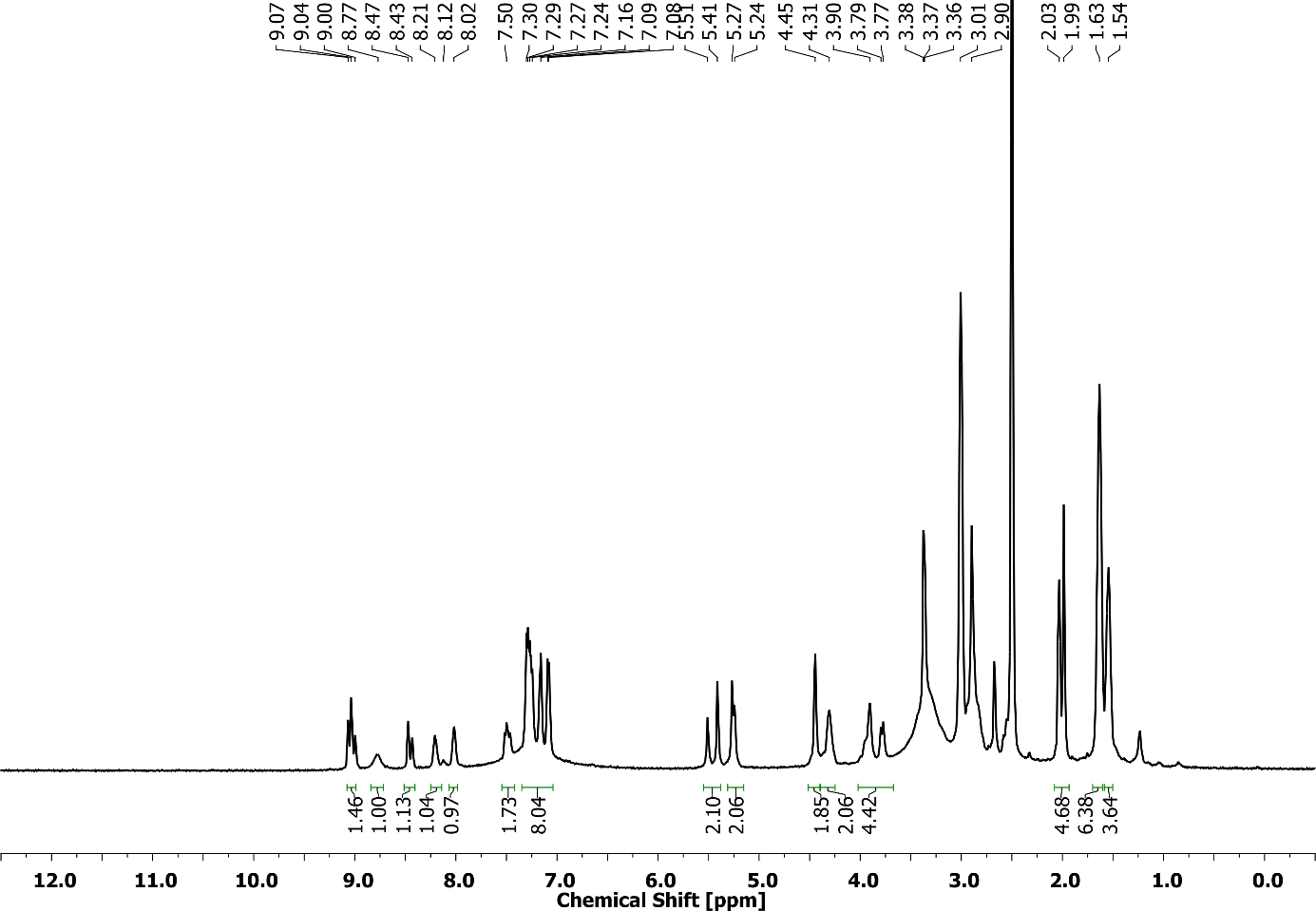


Fig. S33: ^1^H NMR spectrum (DMSO-*d*_6_, 400 MHz, 298 K) of compound 31.


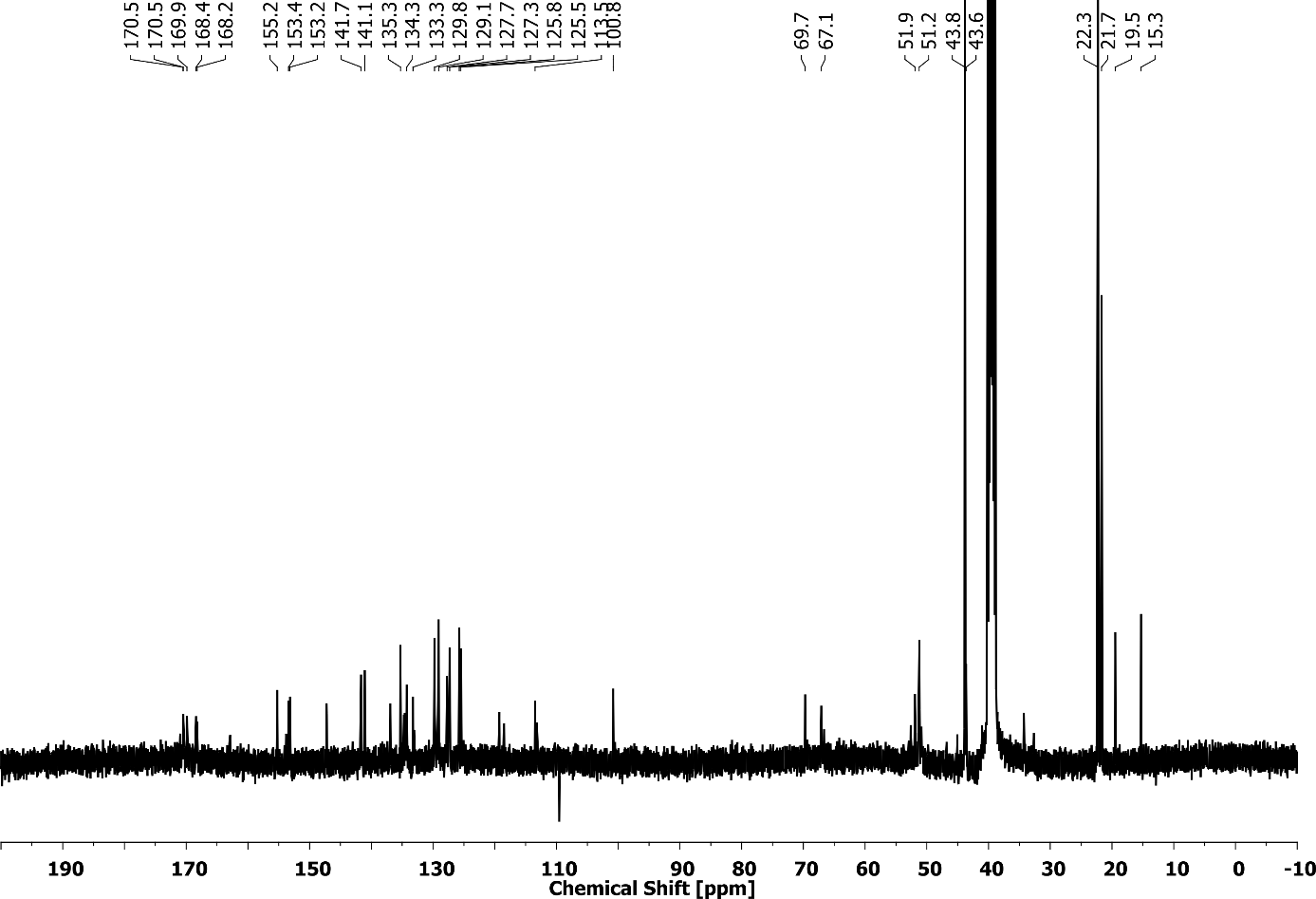


Fig. S34: ^13^C NMR spectrum (DMSO-*d*_6_, 101 MHz, 298 K) of compound 31.

# HPLC-chromatograms


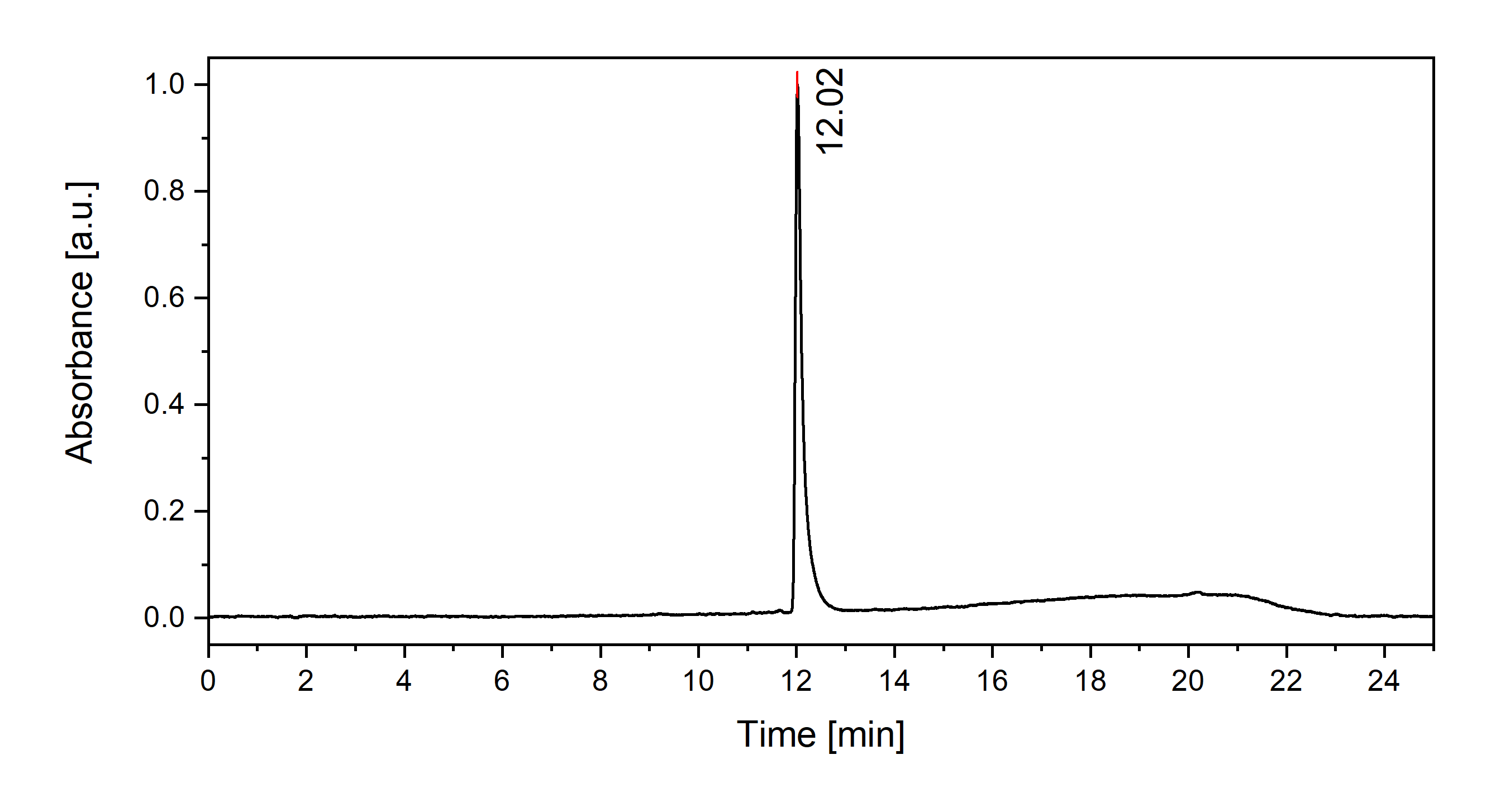


Fig. S35: Analytical RP-HPLC chromatogram (System A) of compound 7.


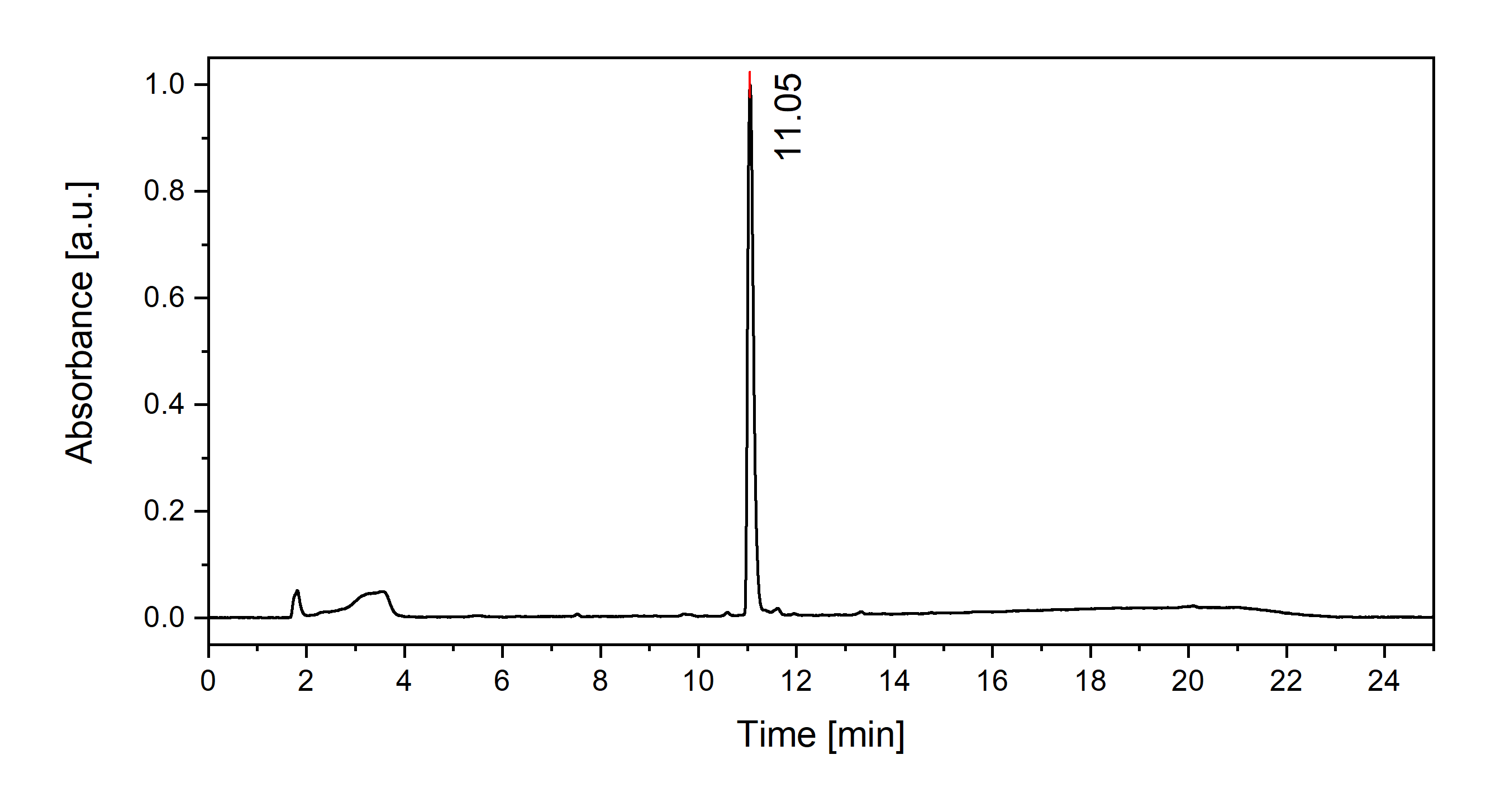


Fig. S36: Analytical RP-HPLC chromatogram (System A) of compound 9.


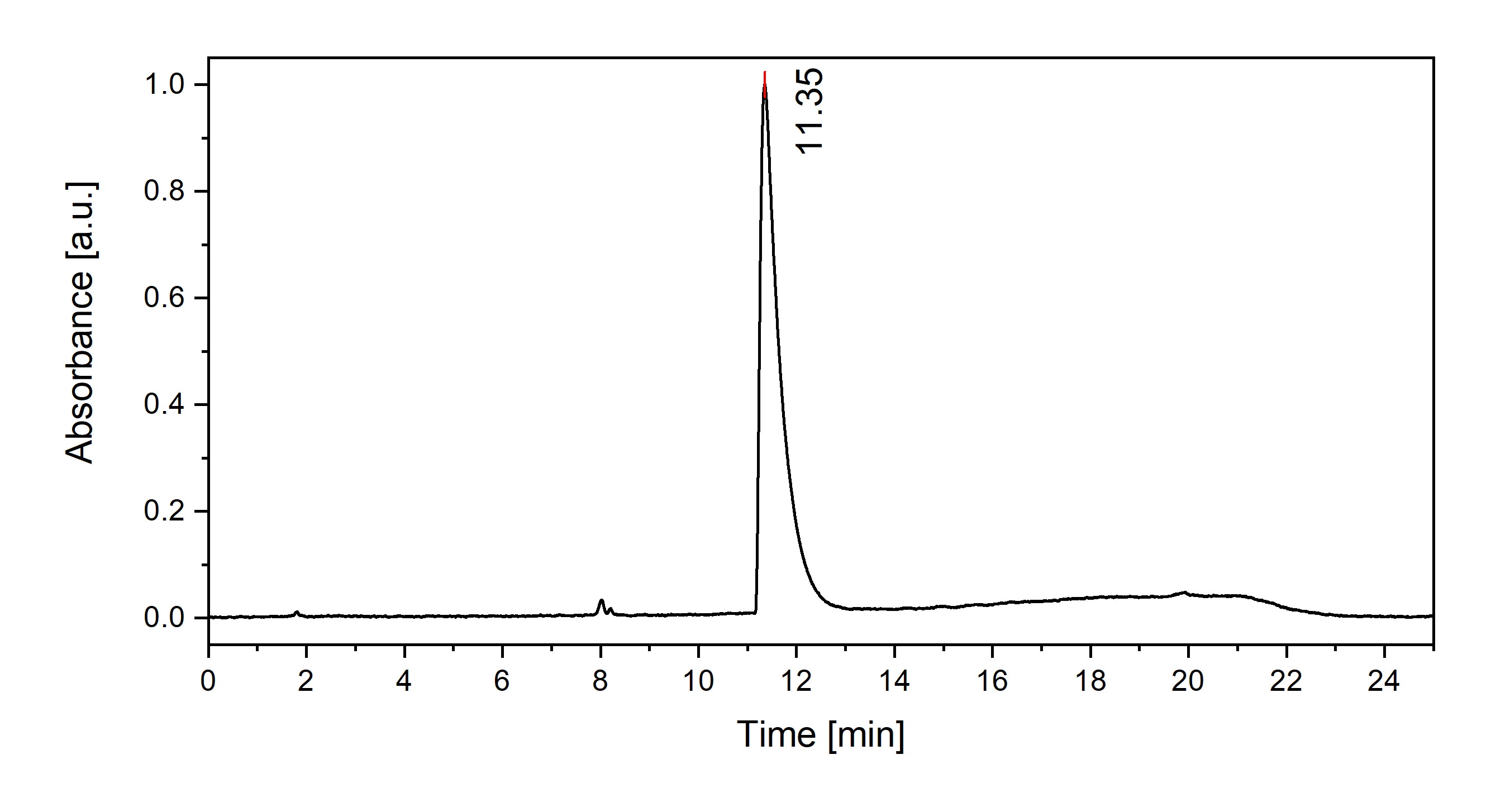


Fig. S37: Analytical RP-HPLC chromatogram (System A) of compound 10.


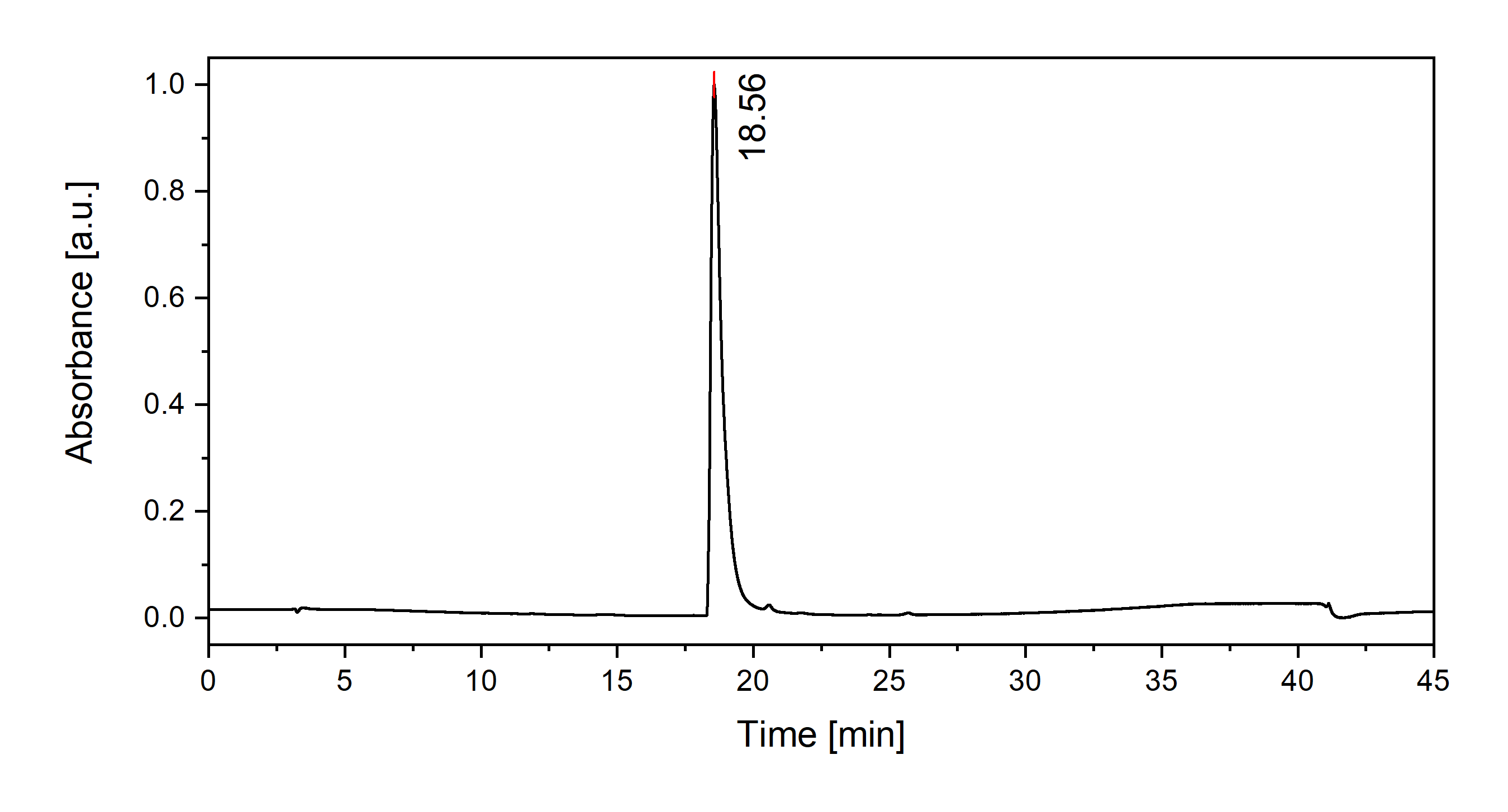


Fig. S38: Analytical RP-HPLC chromatogram (System A) of compound 11.


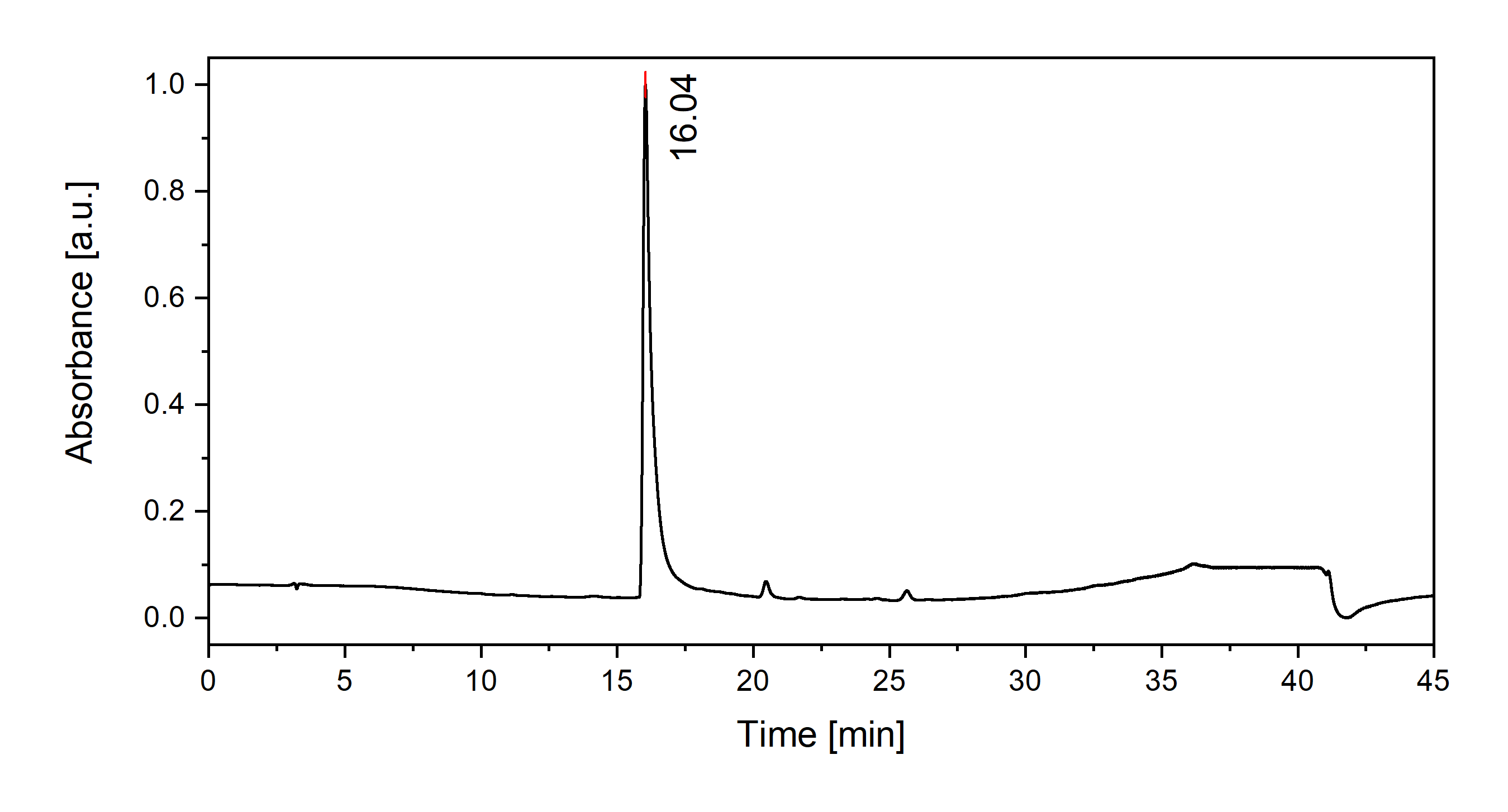


Fig. S39: Analytical RP-HPLC chromatogram (System B) of compound 2.


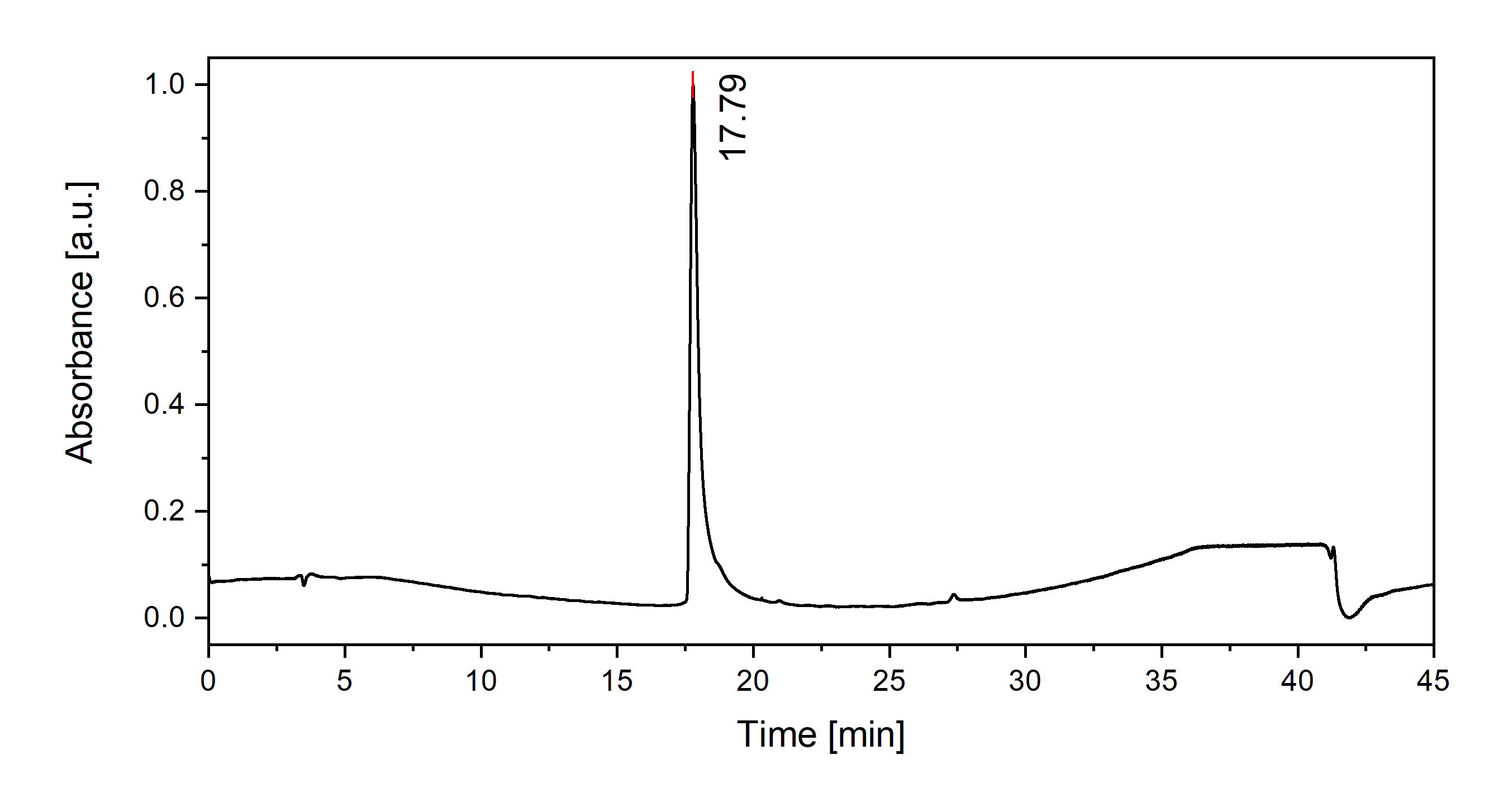


Fig. S40: Analytical RP-HPLC chromatogram (System B) of compound 3.


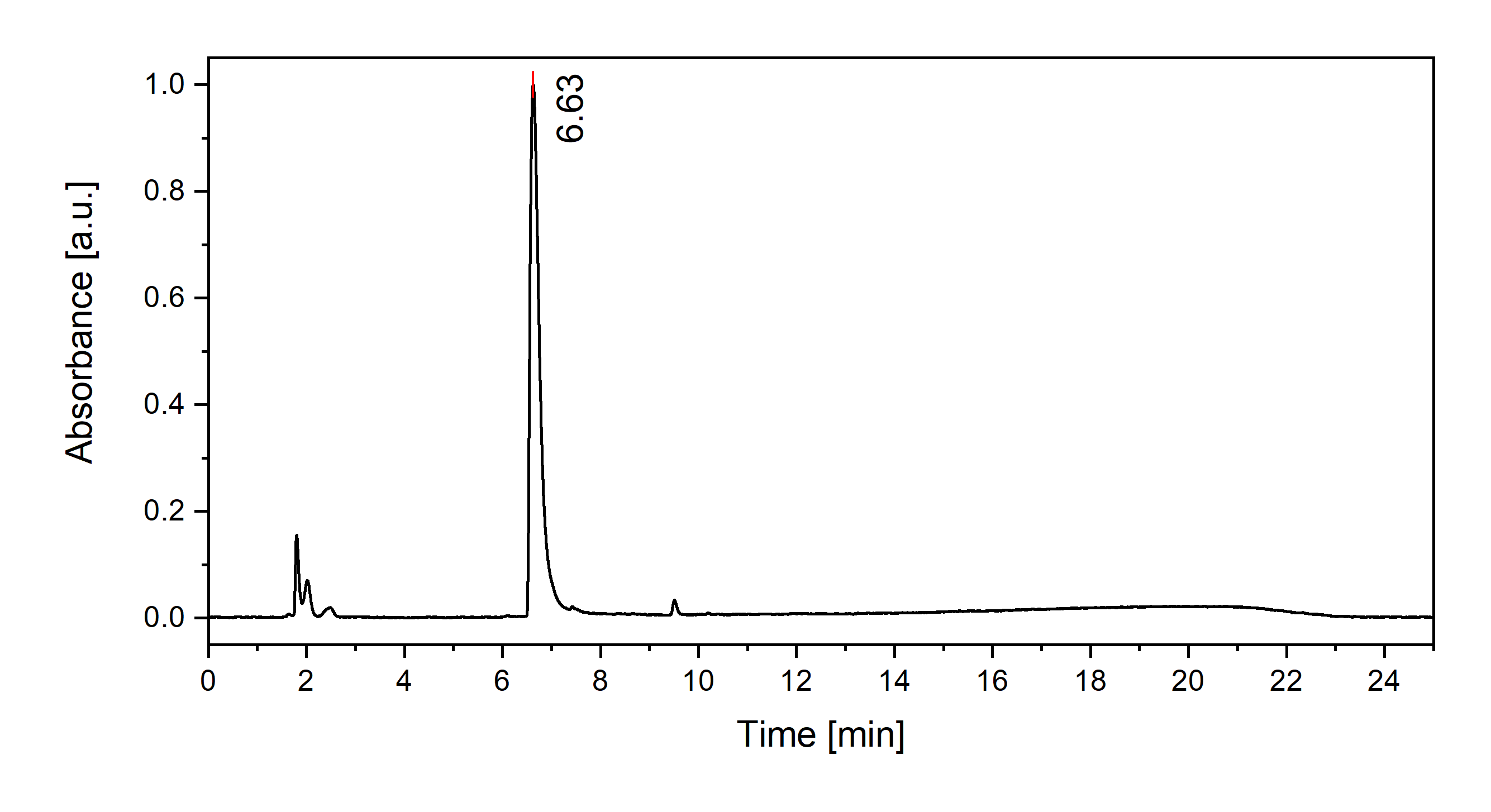


Fig. S41: Analytical RP-HPLC chromatogram (System A) of compound 20.


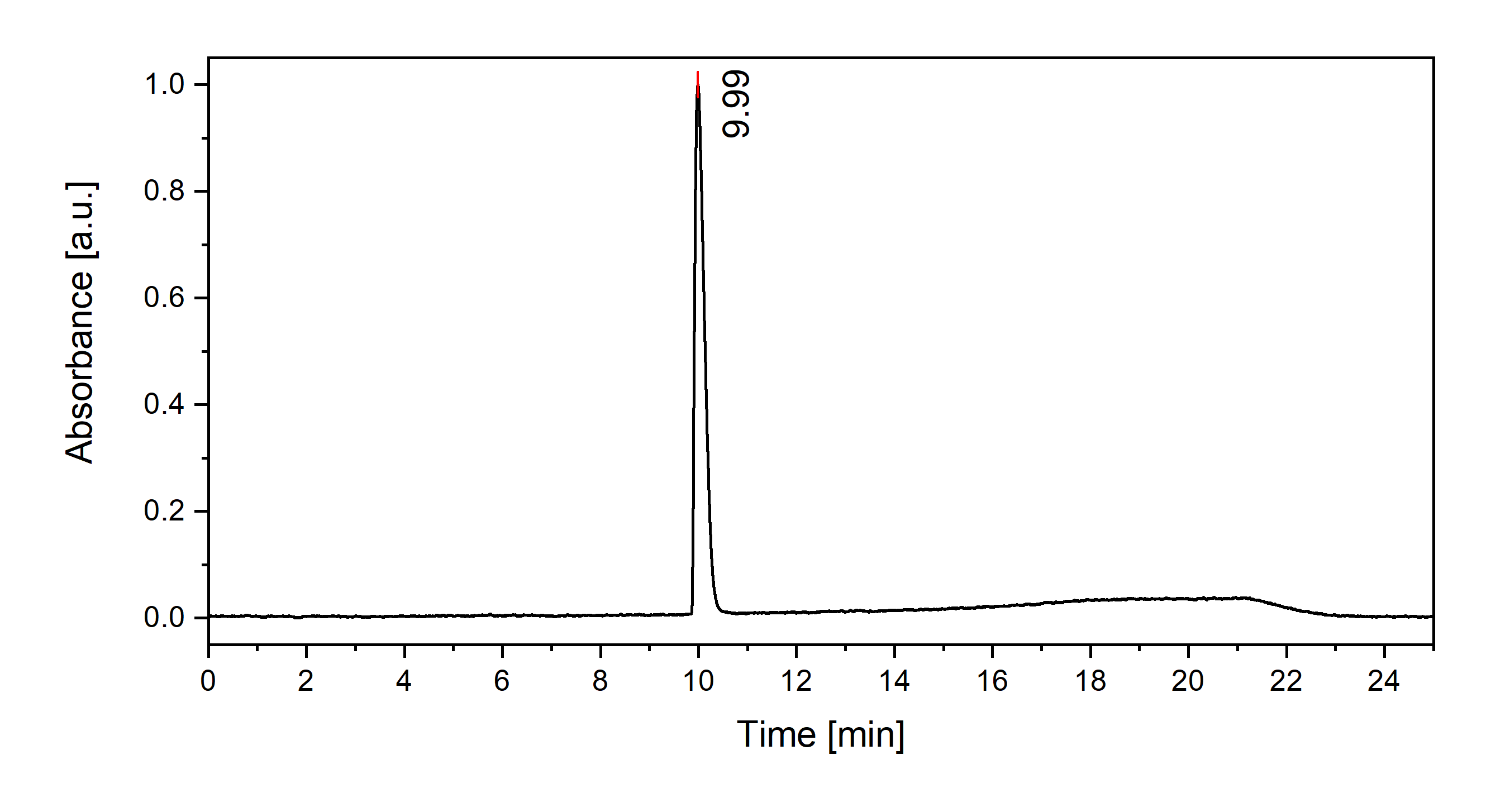


Fig. S42: Analytical RP-HPLC chromatogram (System A) of compound 30.


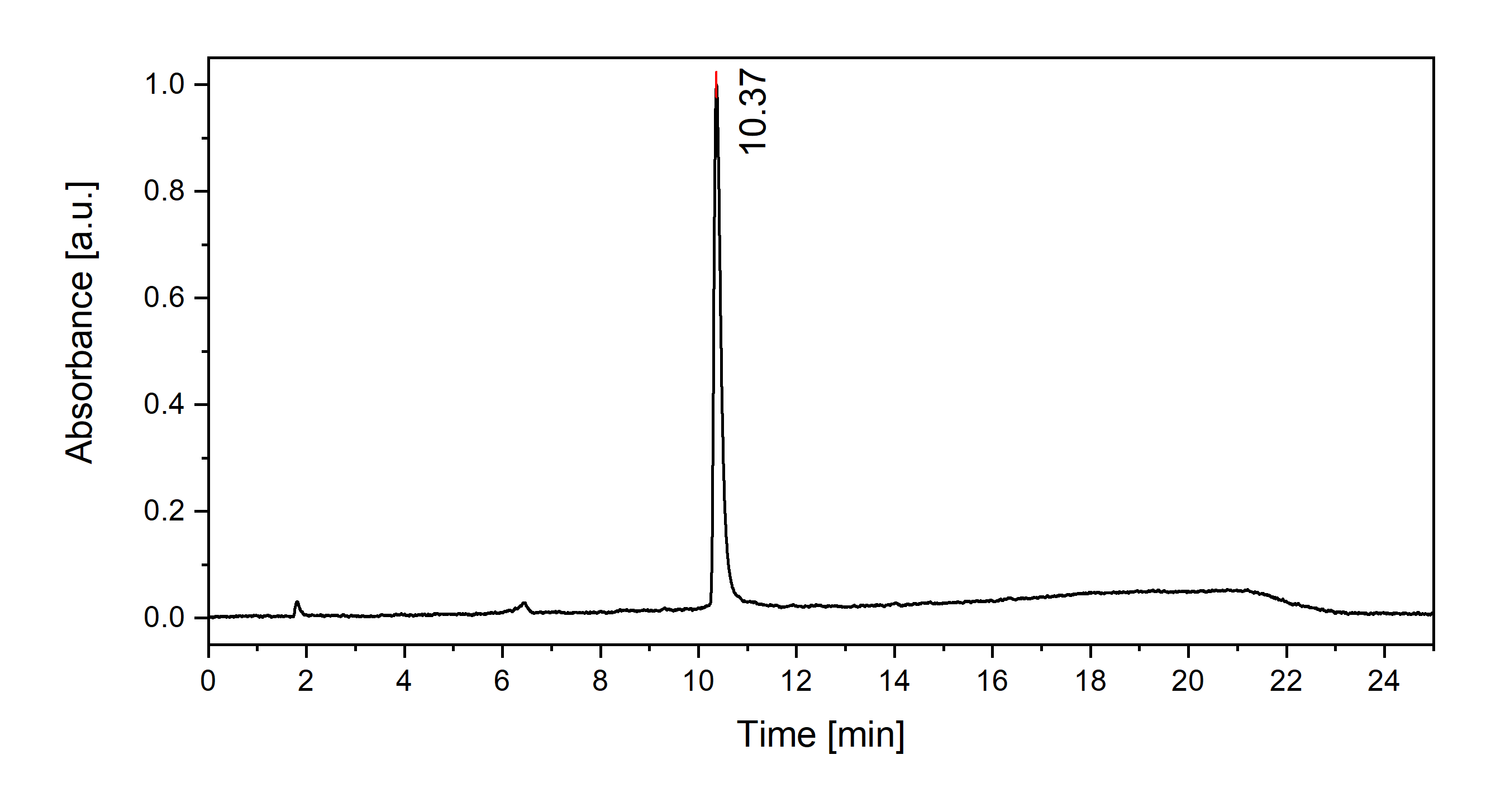


Fig. S43: Analytical RP-HPLC chromatogram (System A) of compound 31.


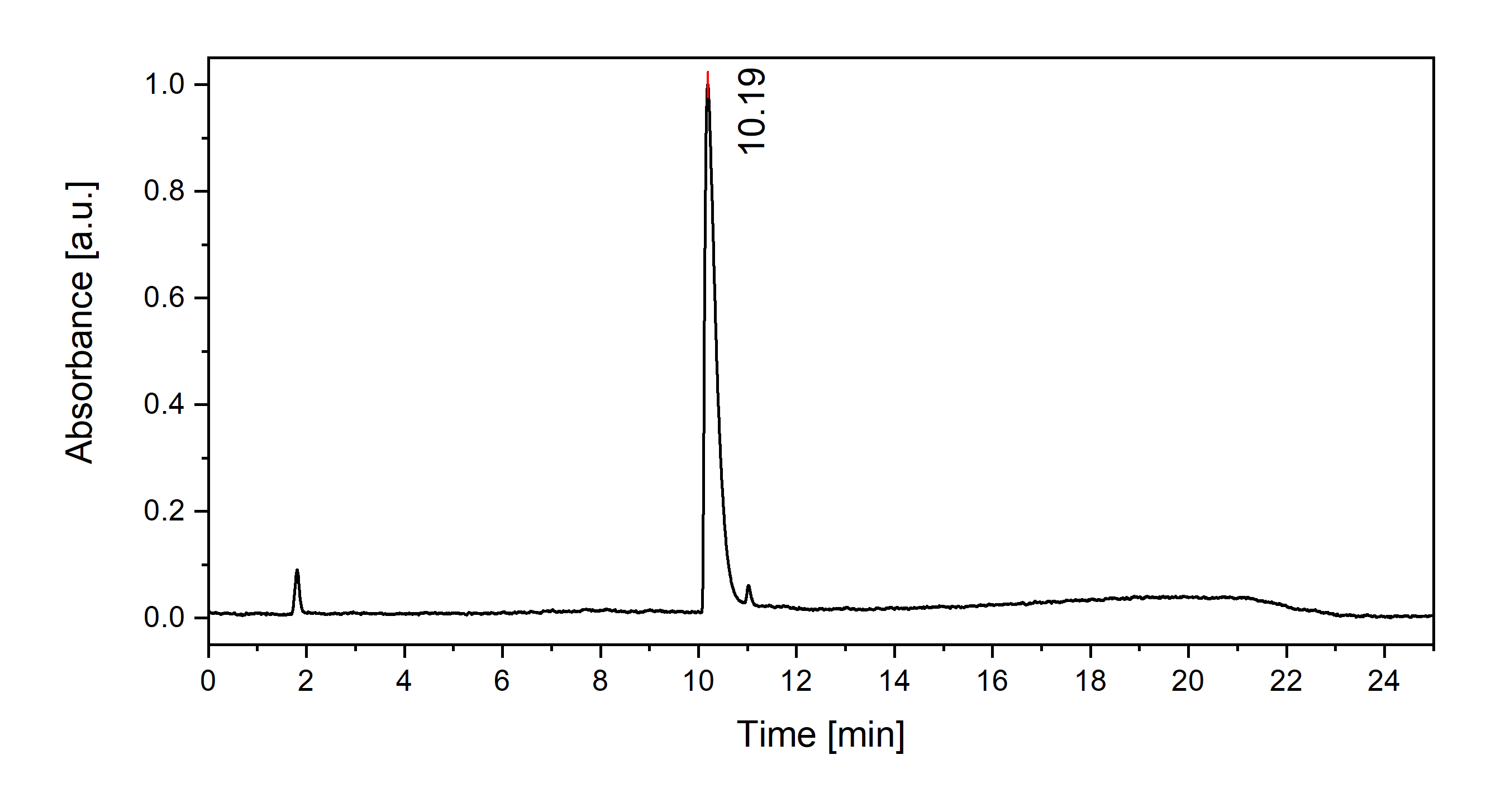


Fig. S44: Analytical RP-HPLC chromatogram (System A) of compound 34.


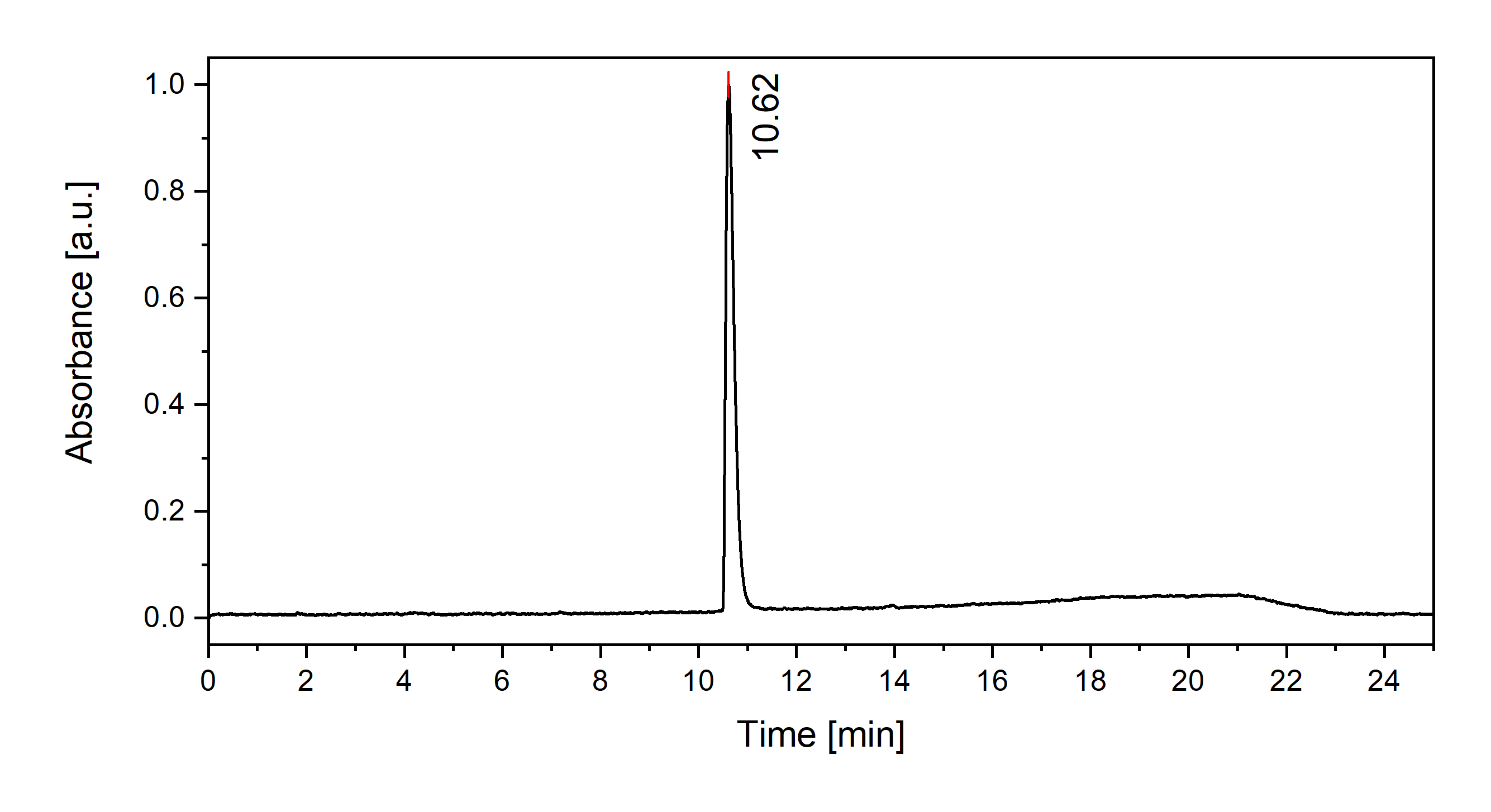


Fig. S45: Analytical RP-HPLC chromatogram (System A) of compound 35.


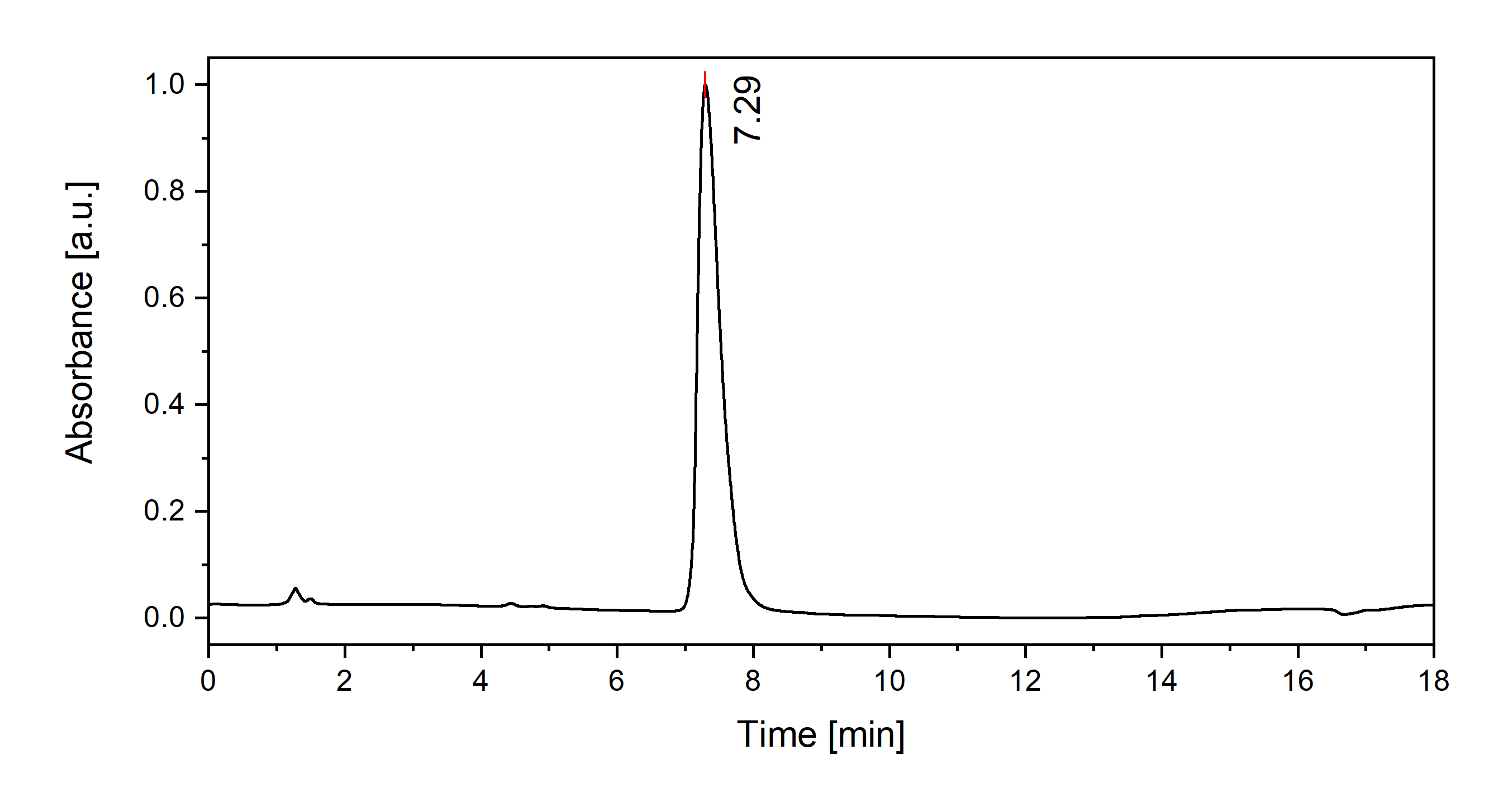


Fig. S46: Analytical RP-HPLC chromatogram (System C) of compound 36.


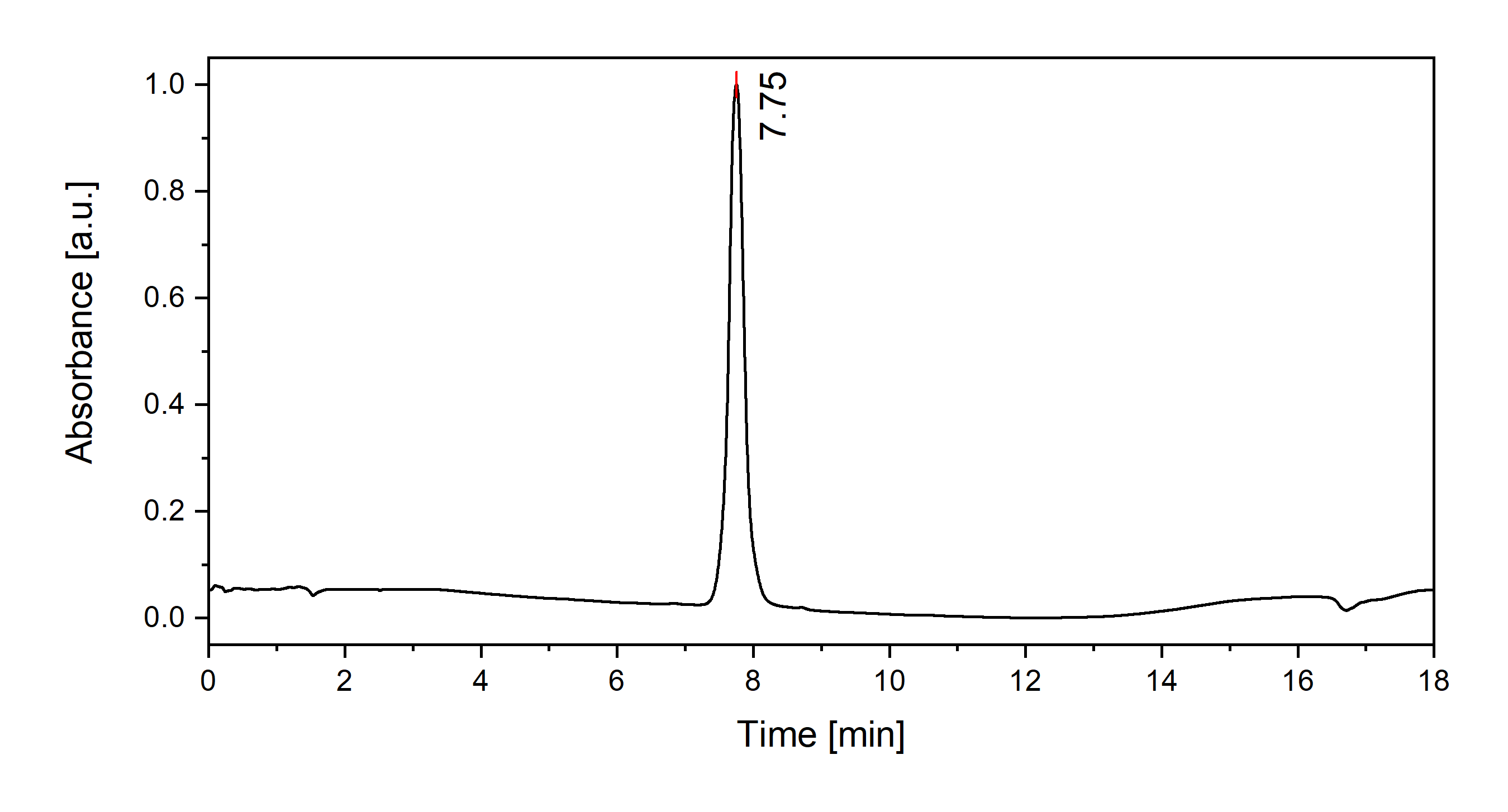


Fig. S47: Analytical RP-HPLC chromatogram (System C) of compound 37.

# High ResolutionMass Spectra


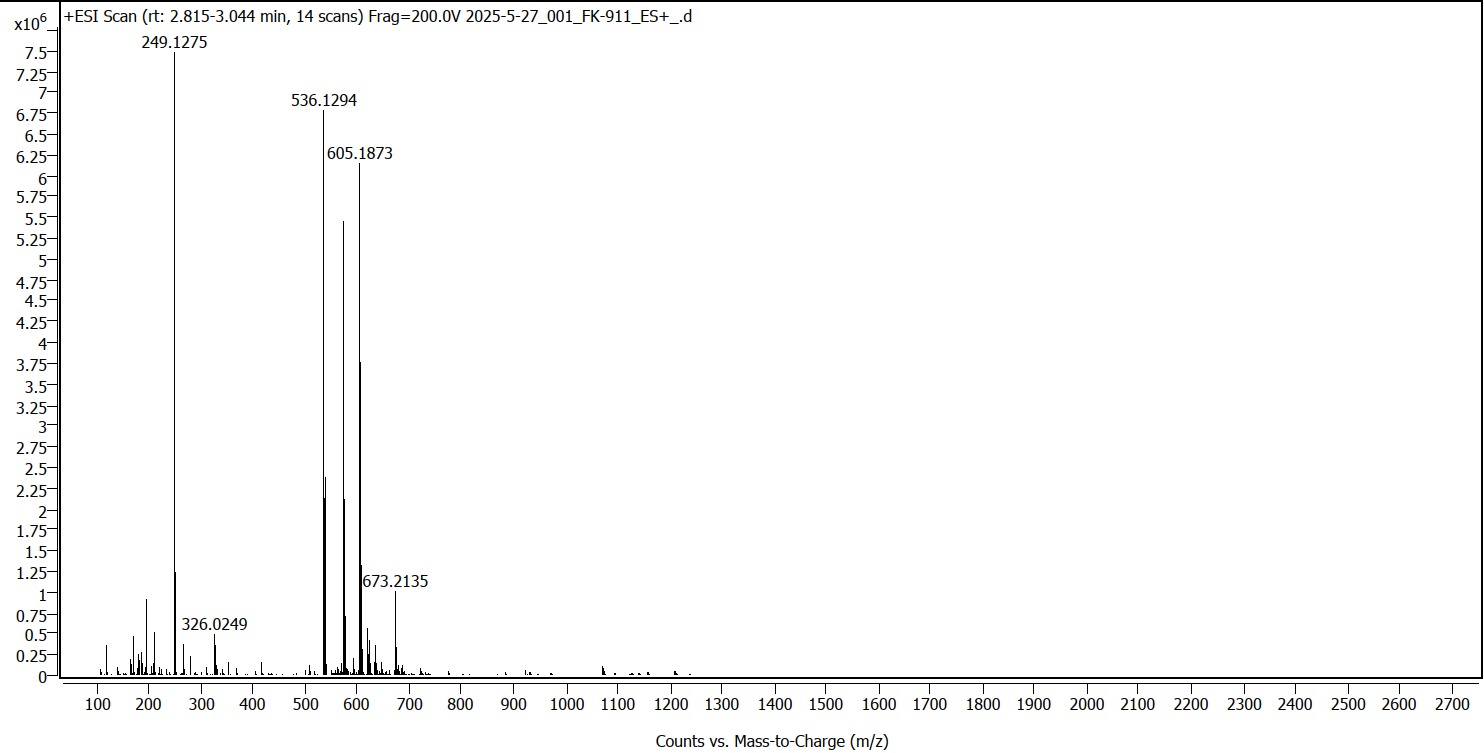


Fig. S48: HR-MS-Spectrum (ESI+) of compound 5.


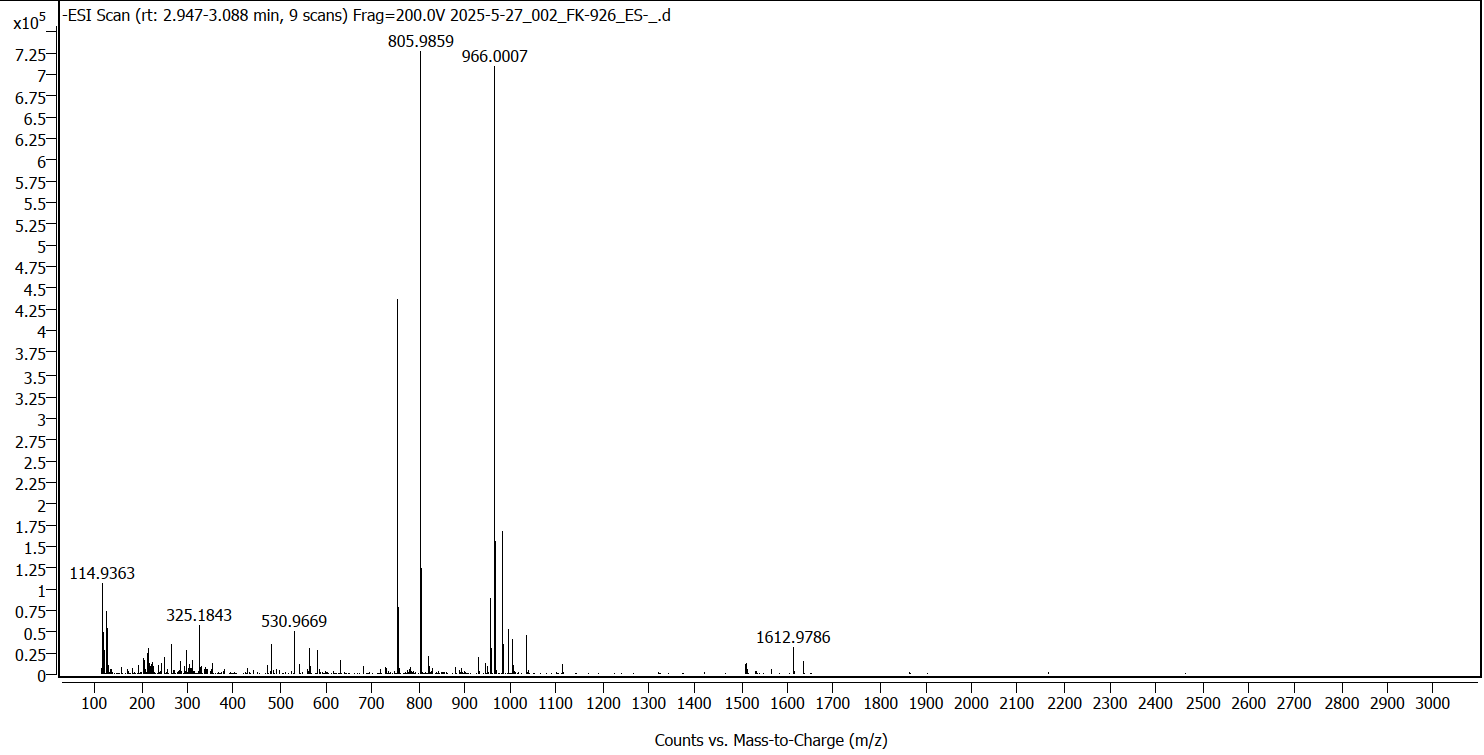


Fig. S49: HR-MS-Spectrum (ESI-) of compound 7.


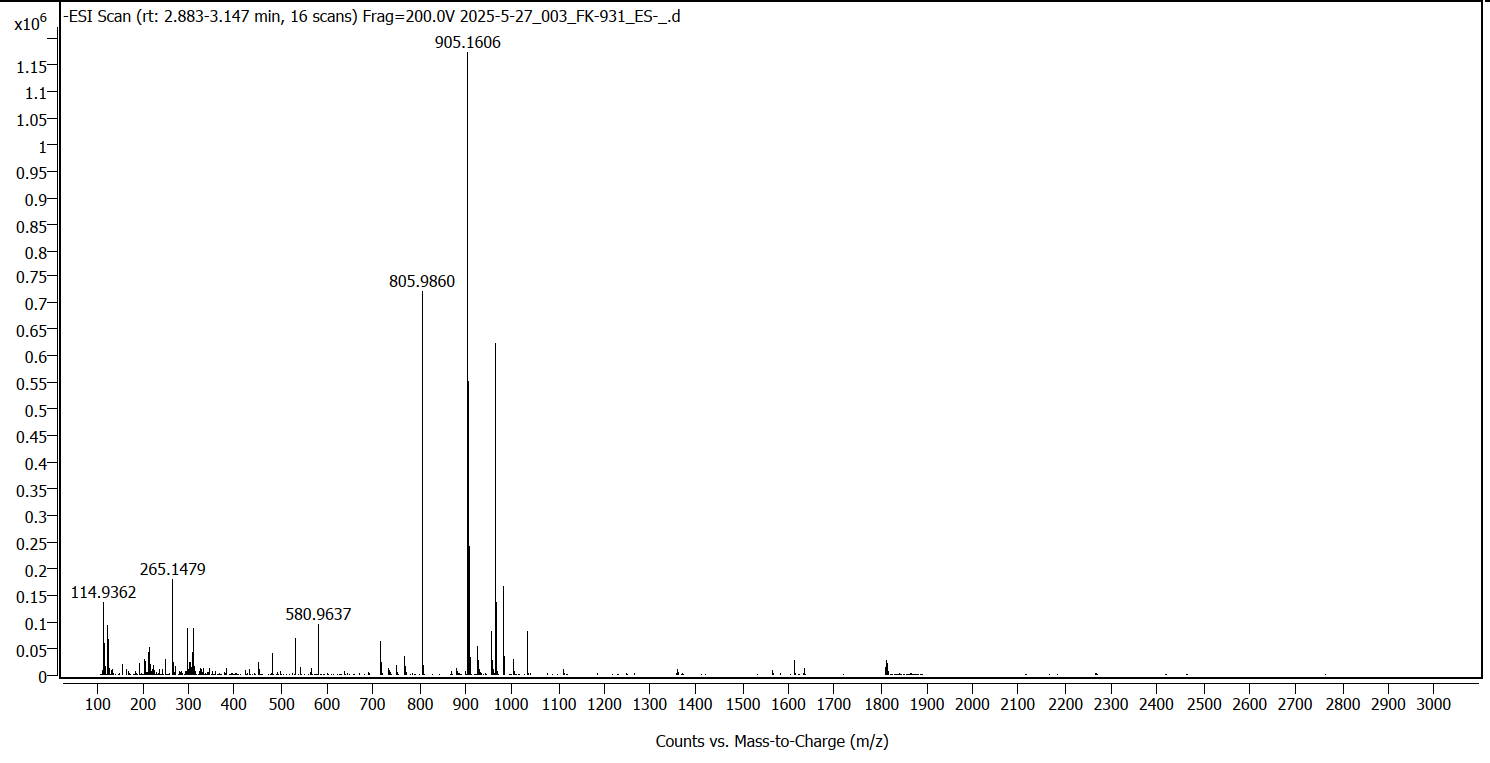


Fig. S50: HR-MS-Spectrum (ESI-) of compound 9.


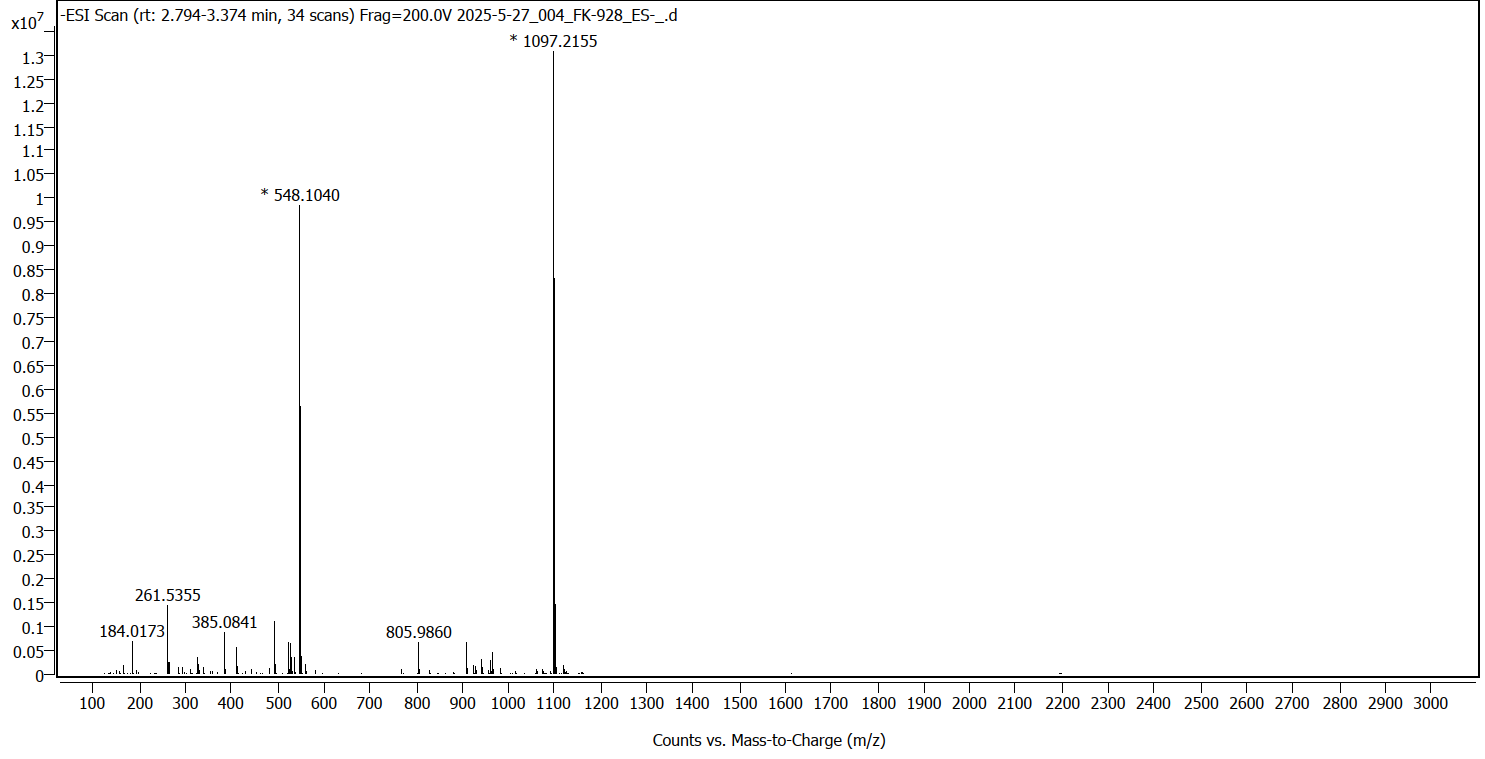


Fig. S51: HR-MS-Spectrum (ESI-) of compound 10.


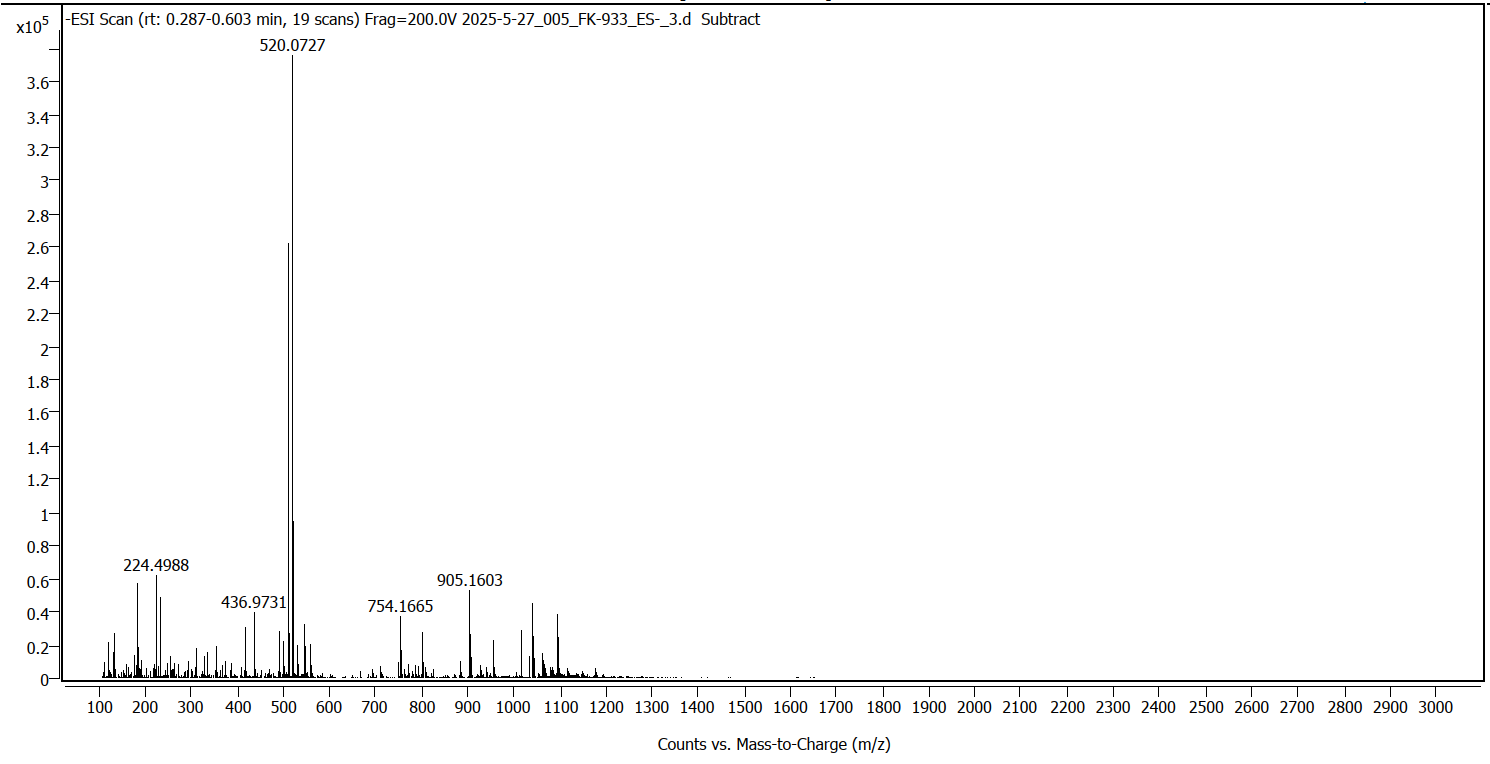


Fig. S52: HR-MS-Spectrum (ESI-) of compound 11.


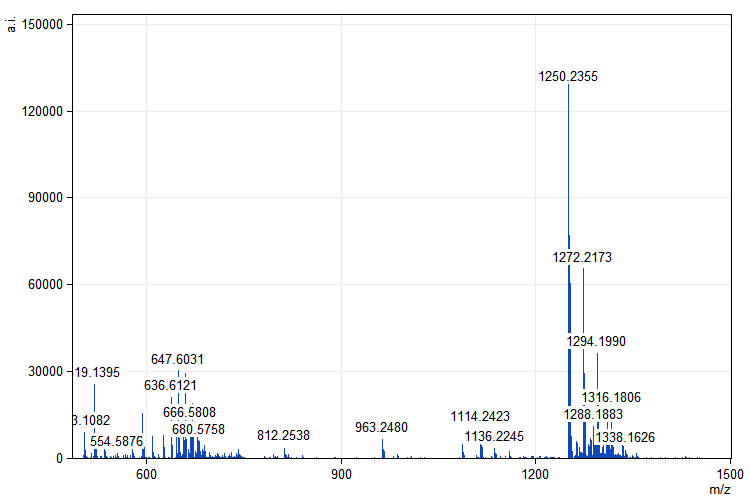


Fig. S53: HR-MS-Spectrum (ESI+) of compound 2.


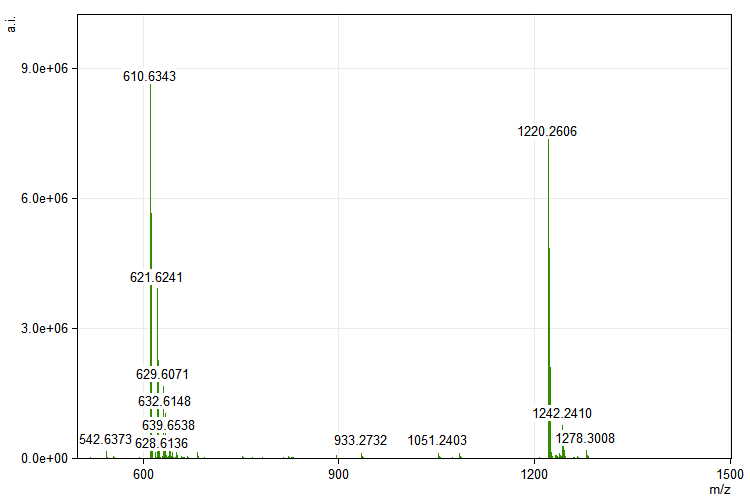


Fig. S54: HR-MS-Spectrum (ESI+) of compound 3.


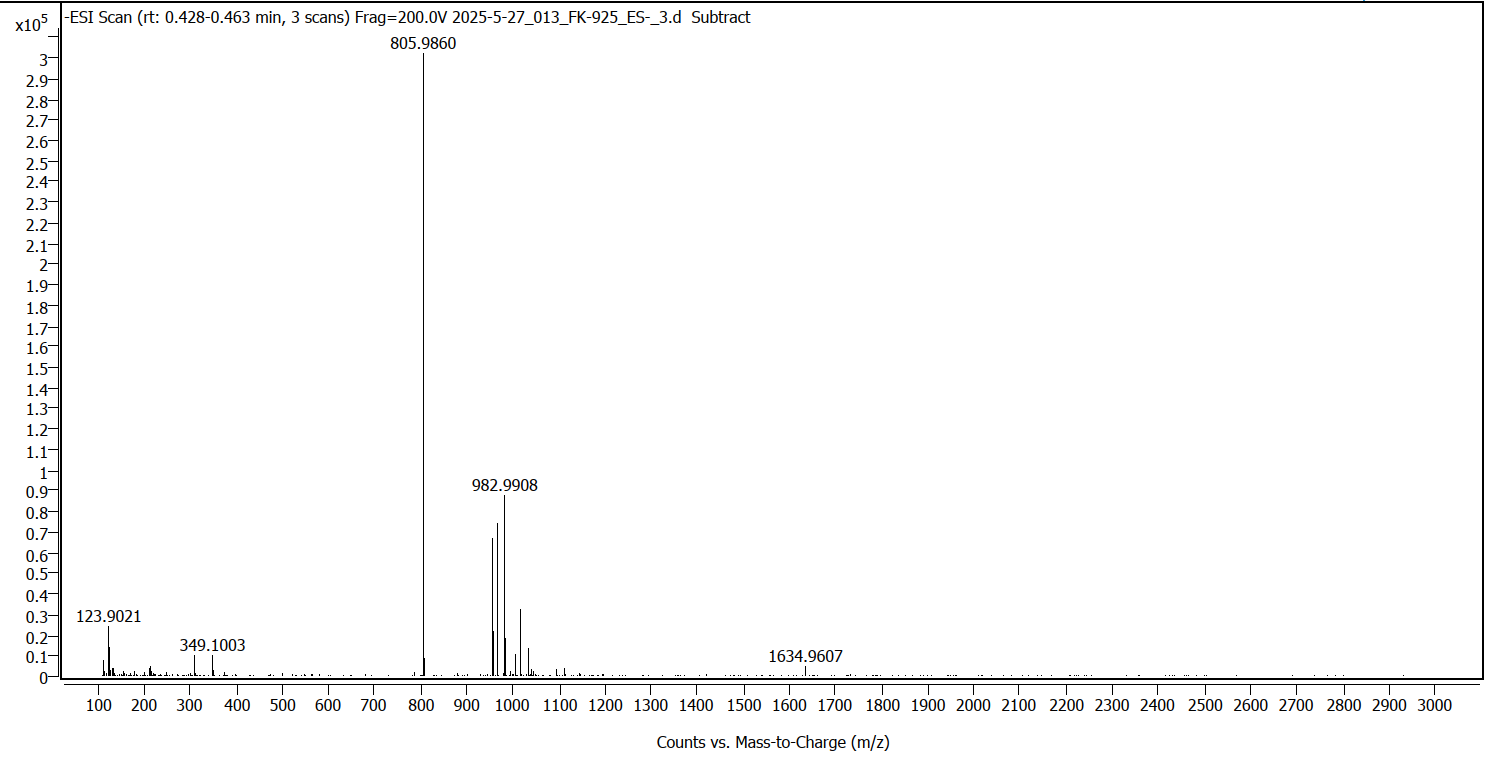


Fig. S55: HR-MS-Spectrum (ESI-) of compound 24.


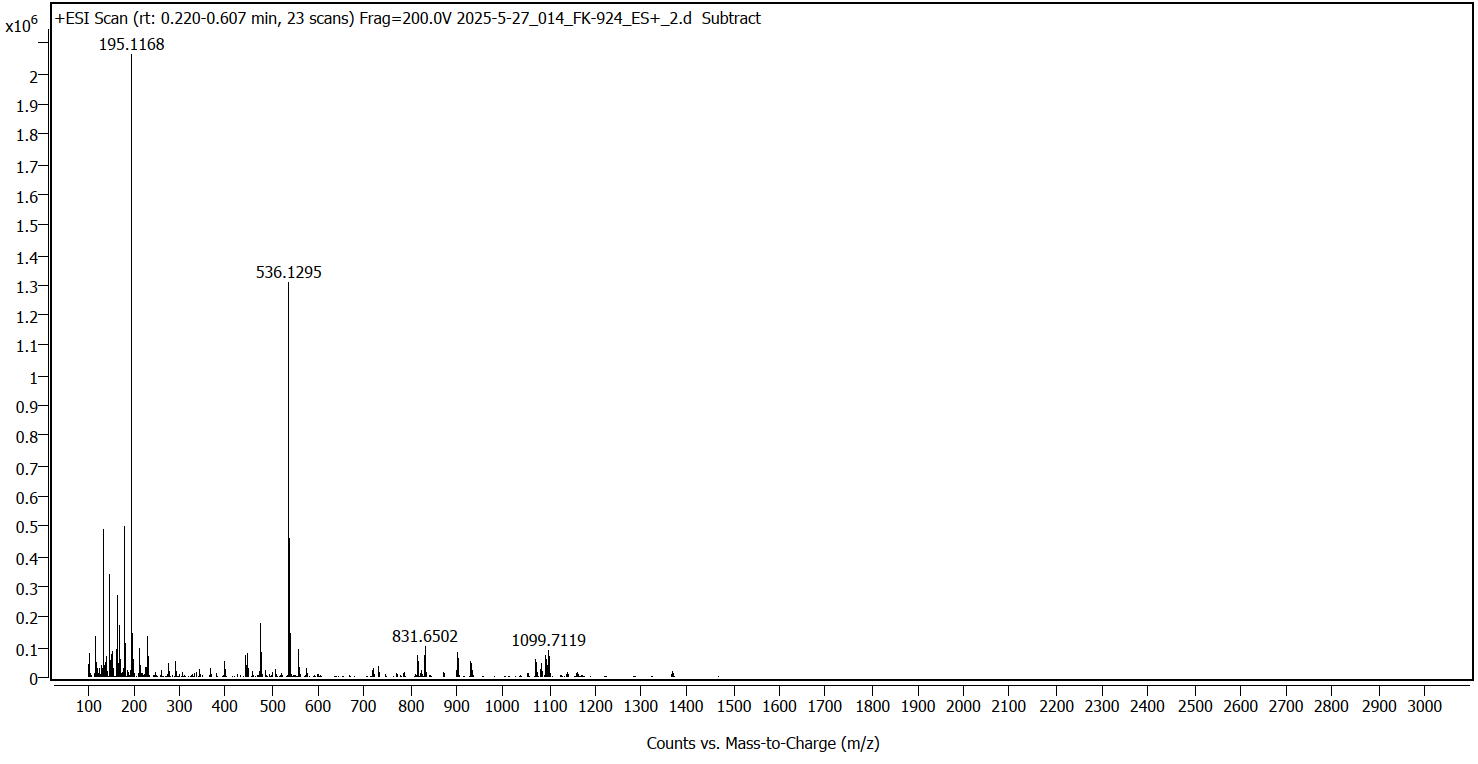


Fig. S56: HR-MS-Spectrum (ESI+) of compound 25.


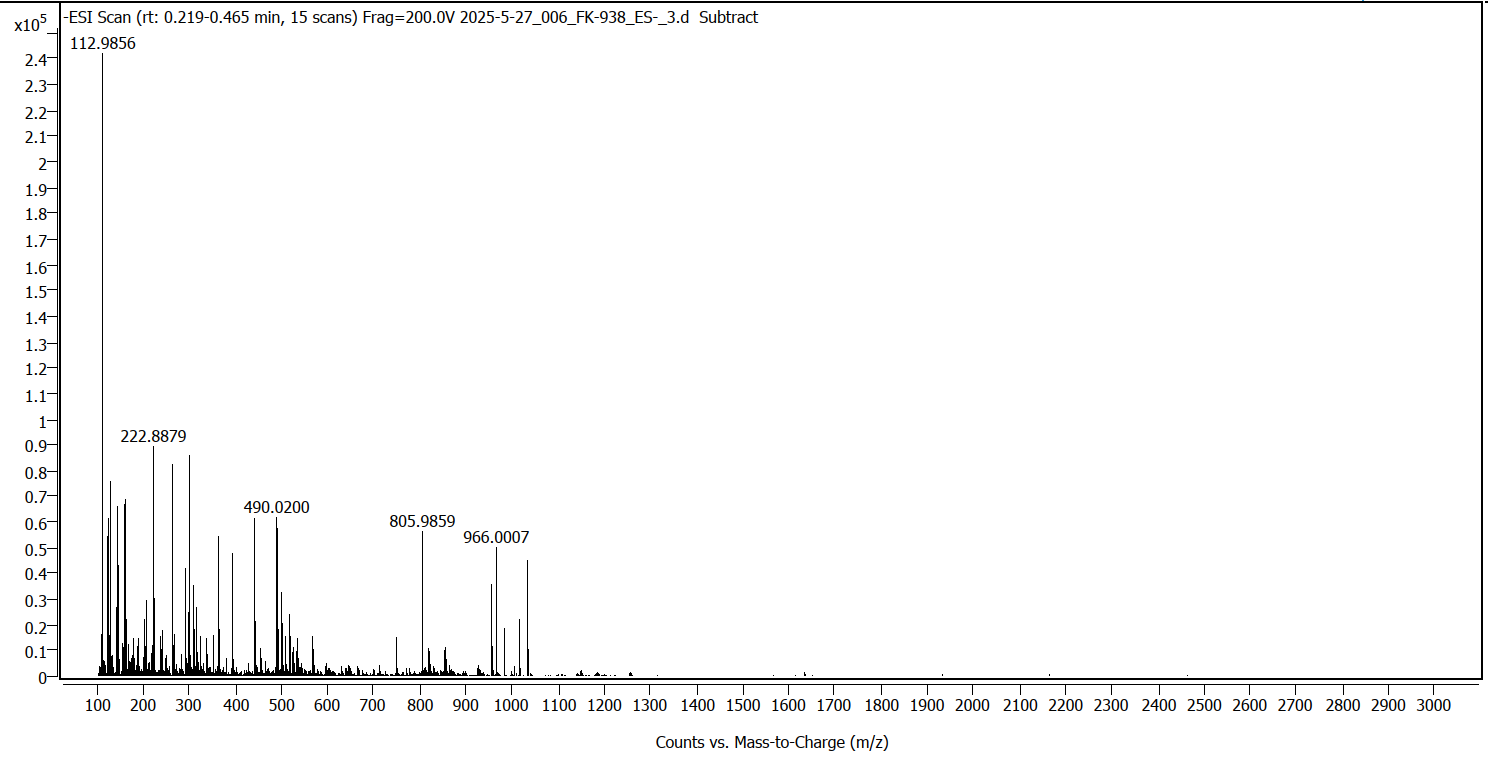


Fig. S57: HR-MS-Spectrum (ESI-) of compound 26.


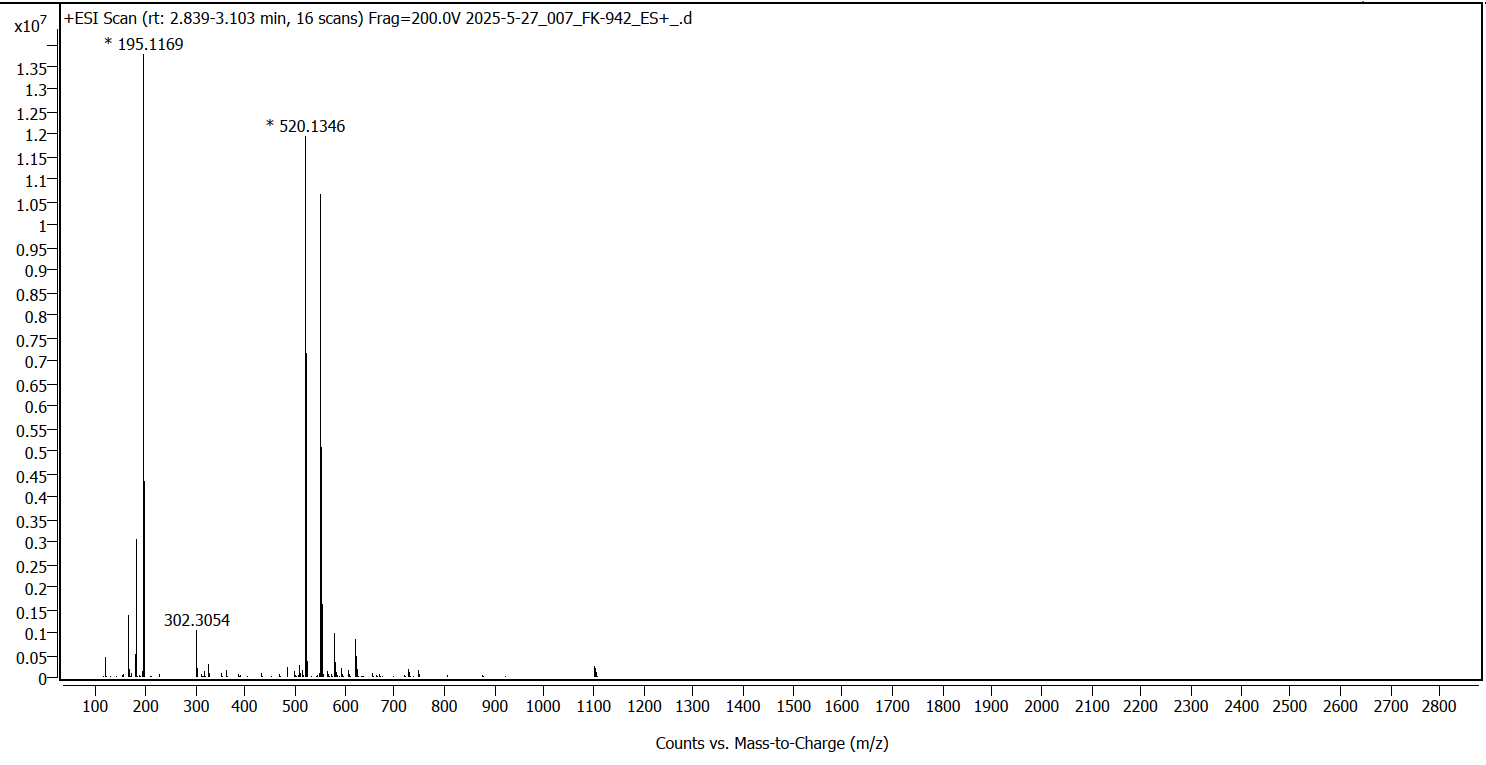


Fig. S58: HR-MS-Spectrum (ESI+) of compound 27.


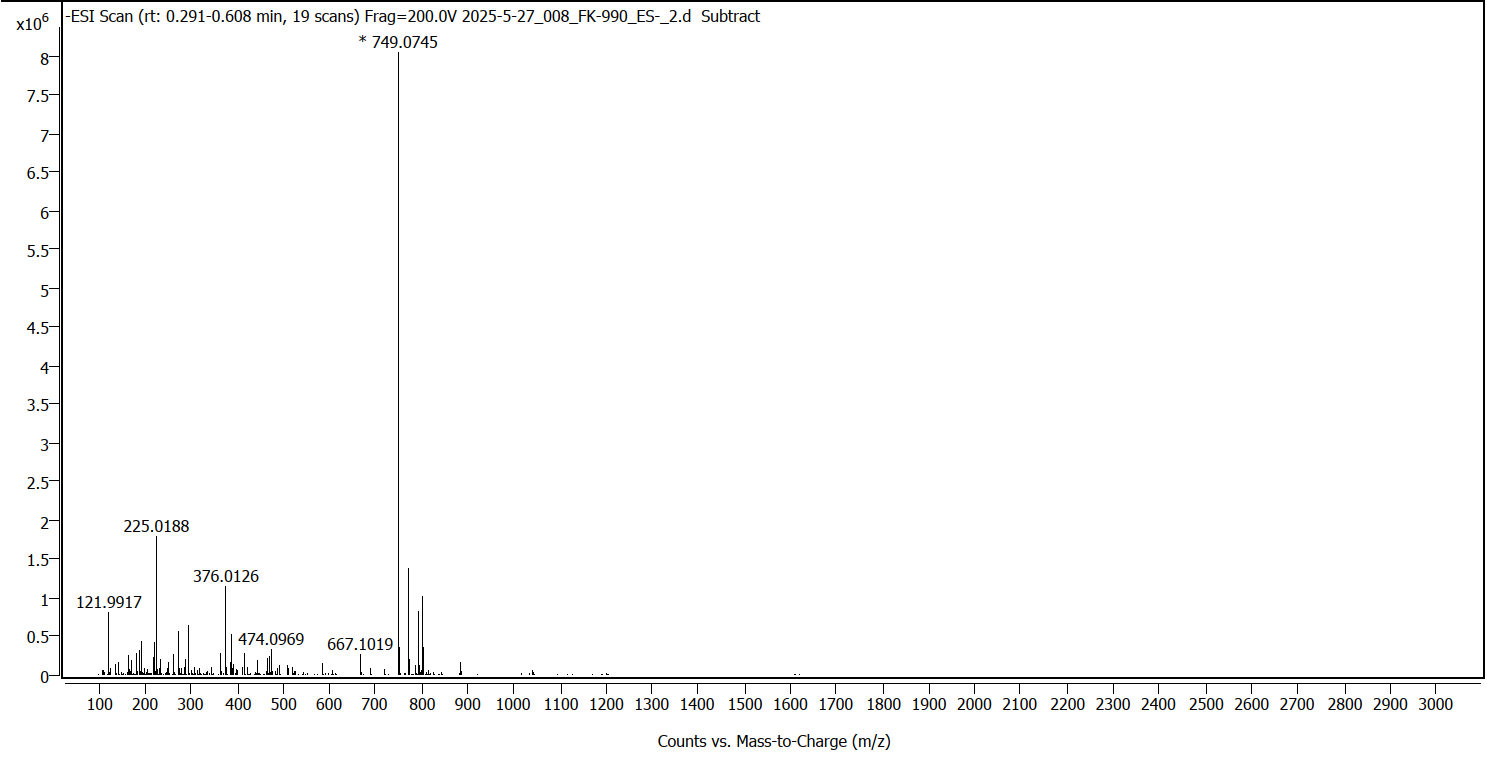


Fig. S59: HR-MS-Spectrum (ESI-) of compound 20.


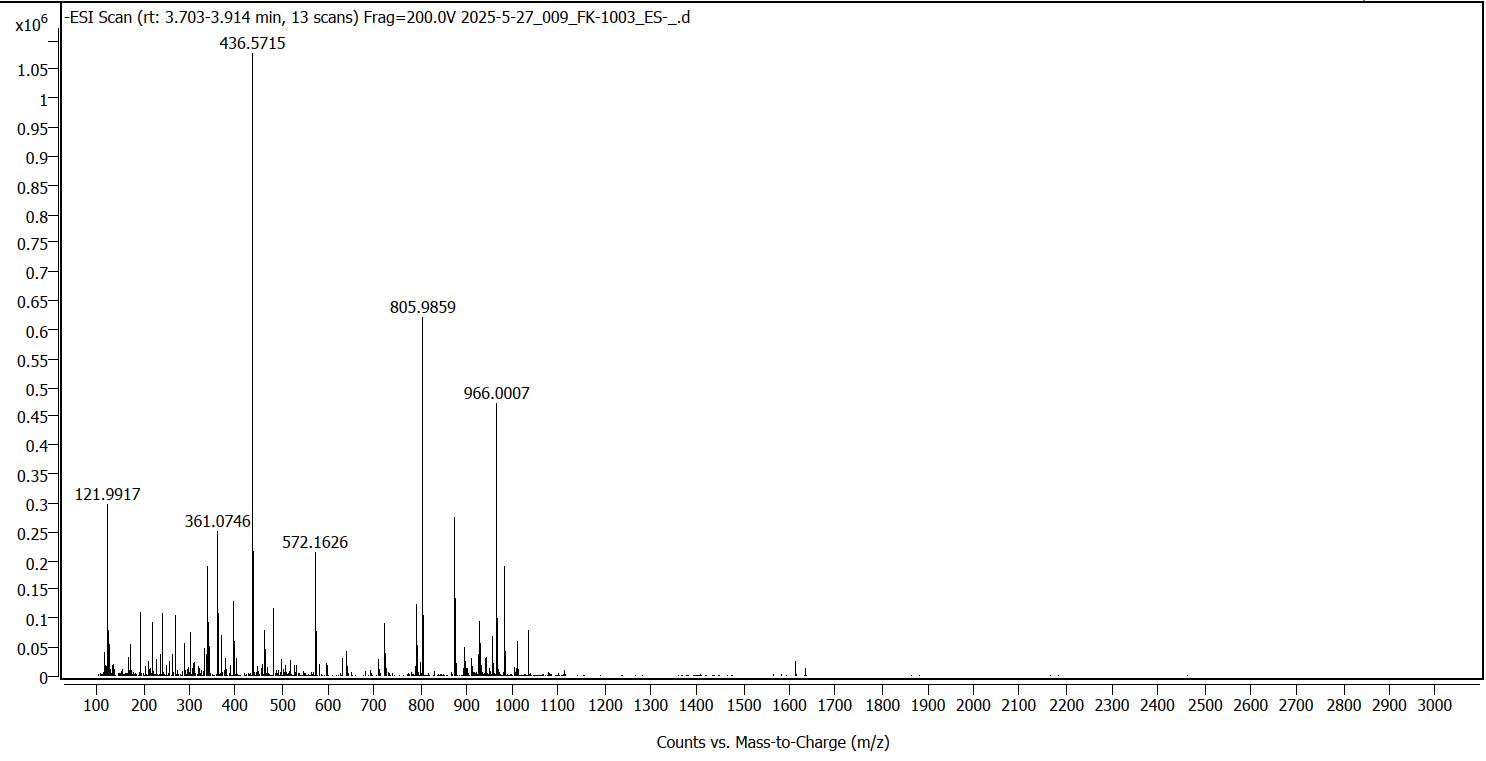


Fig. S60: HR-MS-Spectrum (ESI-) of compound 30.


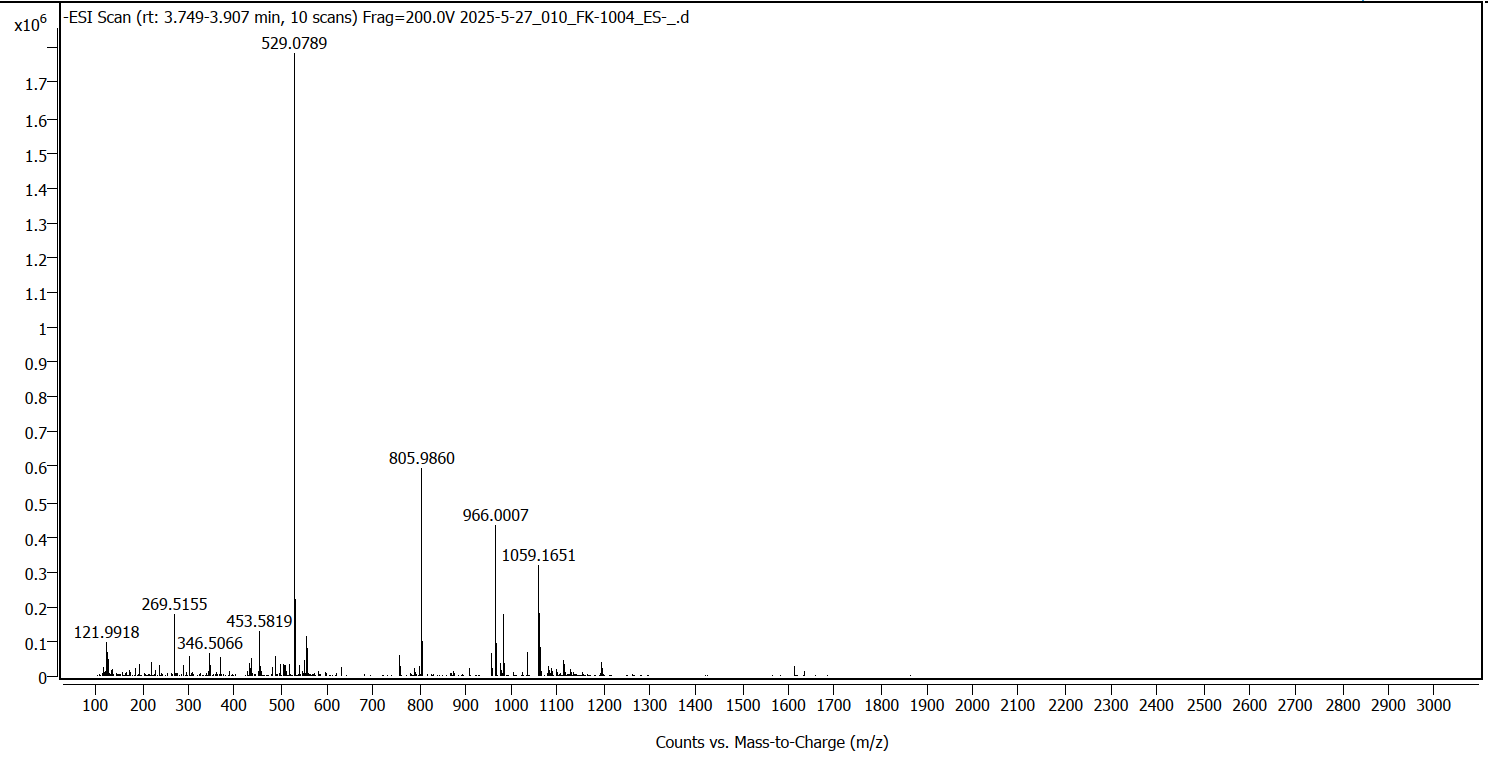


Fig. S61: HR-MS-Spectrum (ESI-) of compound 31.


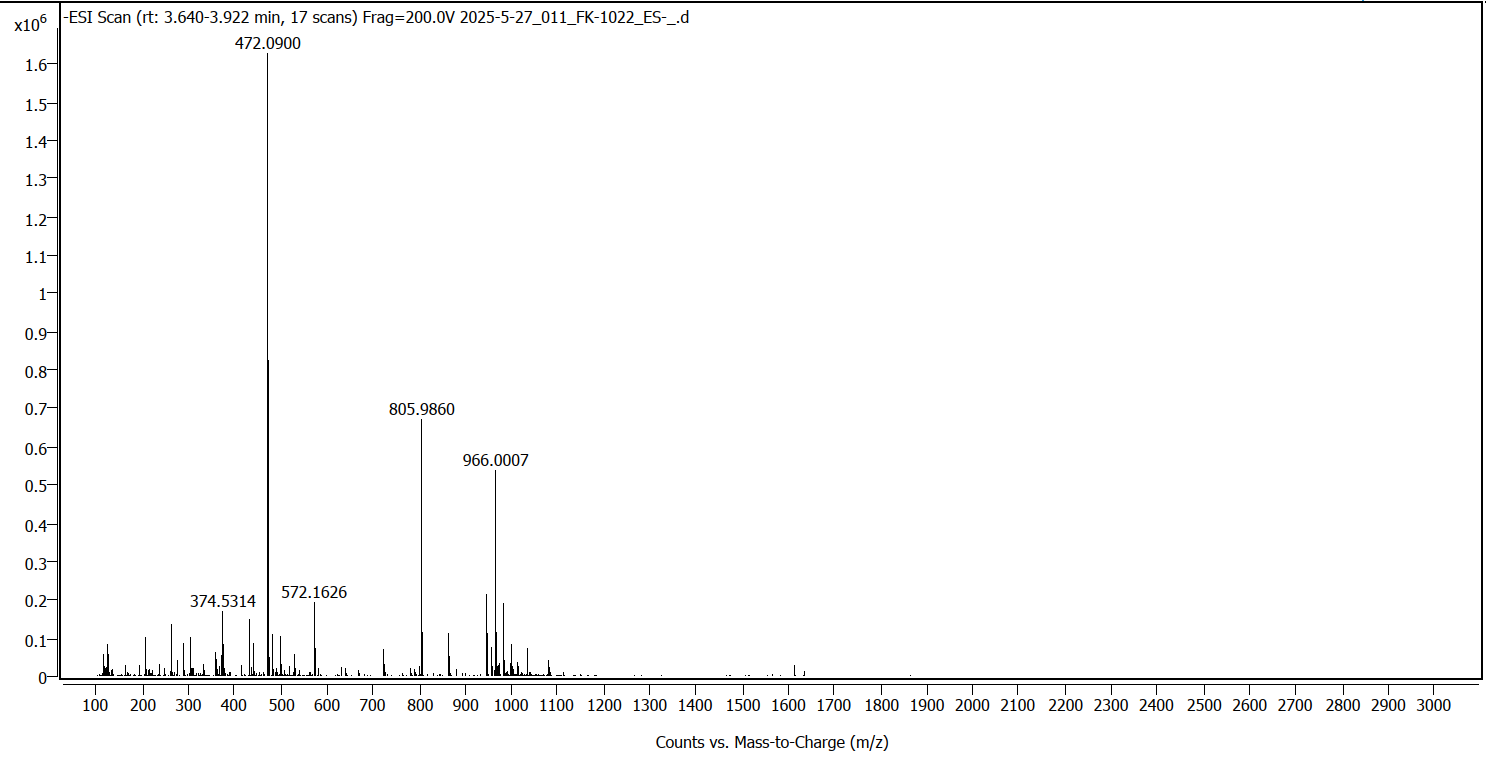


Fig. S62: HR-MS-Spectrum (ESI-) of compound 34.


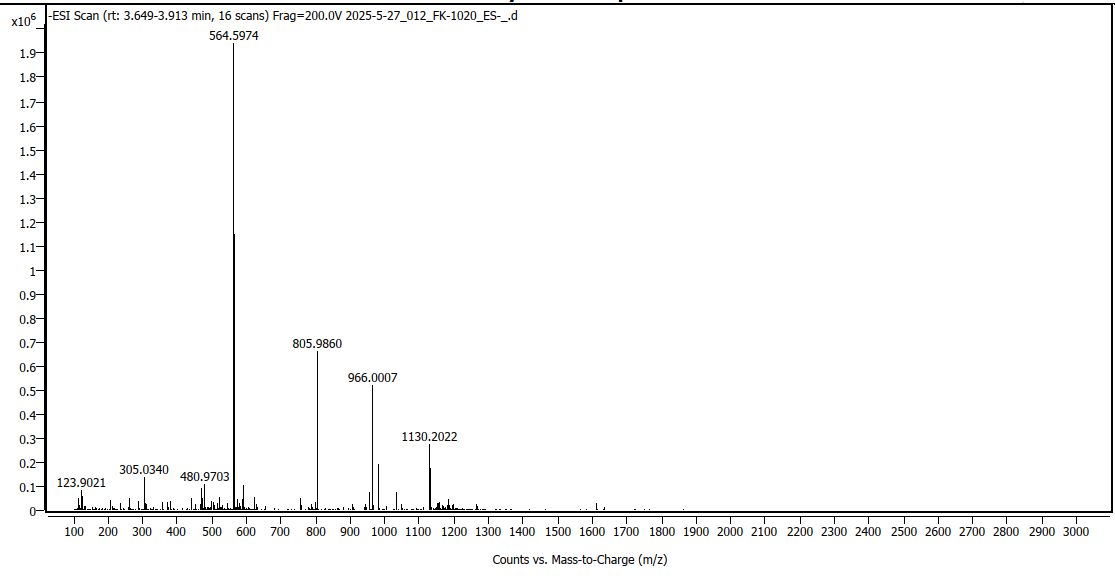


Fig. S63: HR-MS-Spectrum (ESI-) of compound 35.


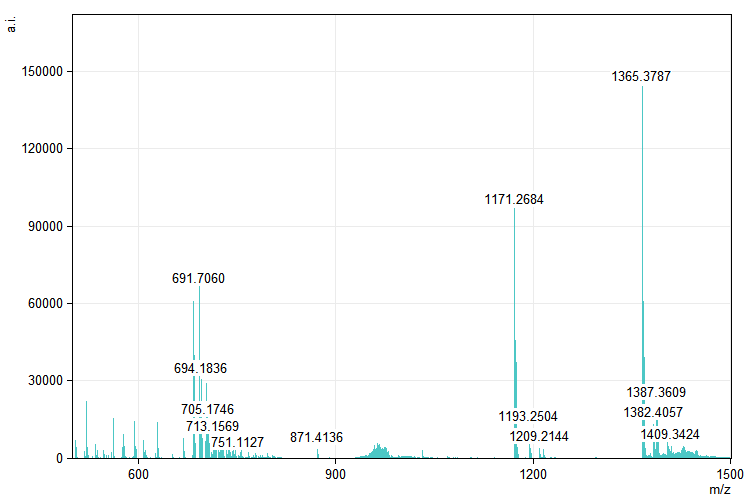


Fig. S64: HR-MS-Spectrum (ESI+) of compound 36.


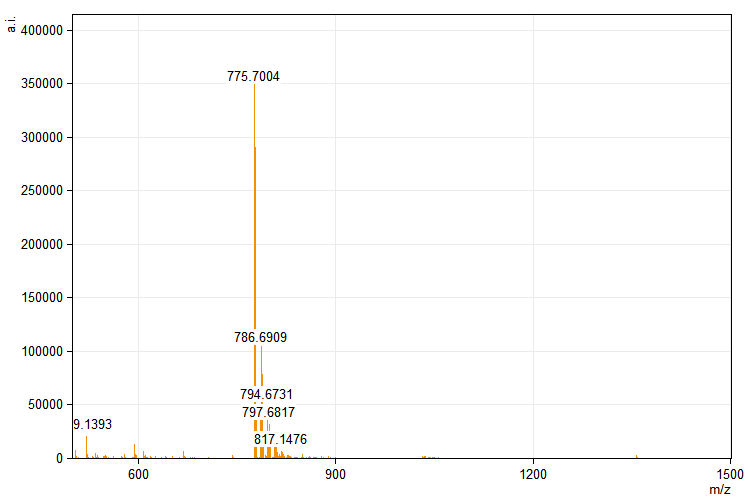


Fig. S65: HR-MS-Spectrum (ESI-) of compound 37.

# Radiochemistry


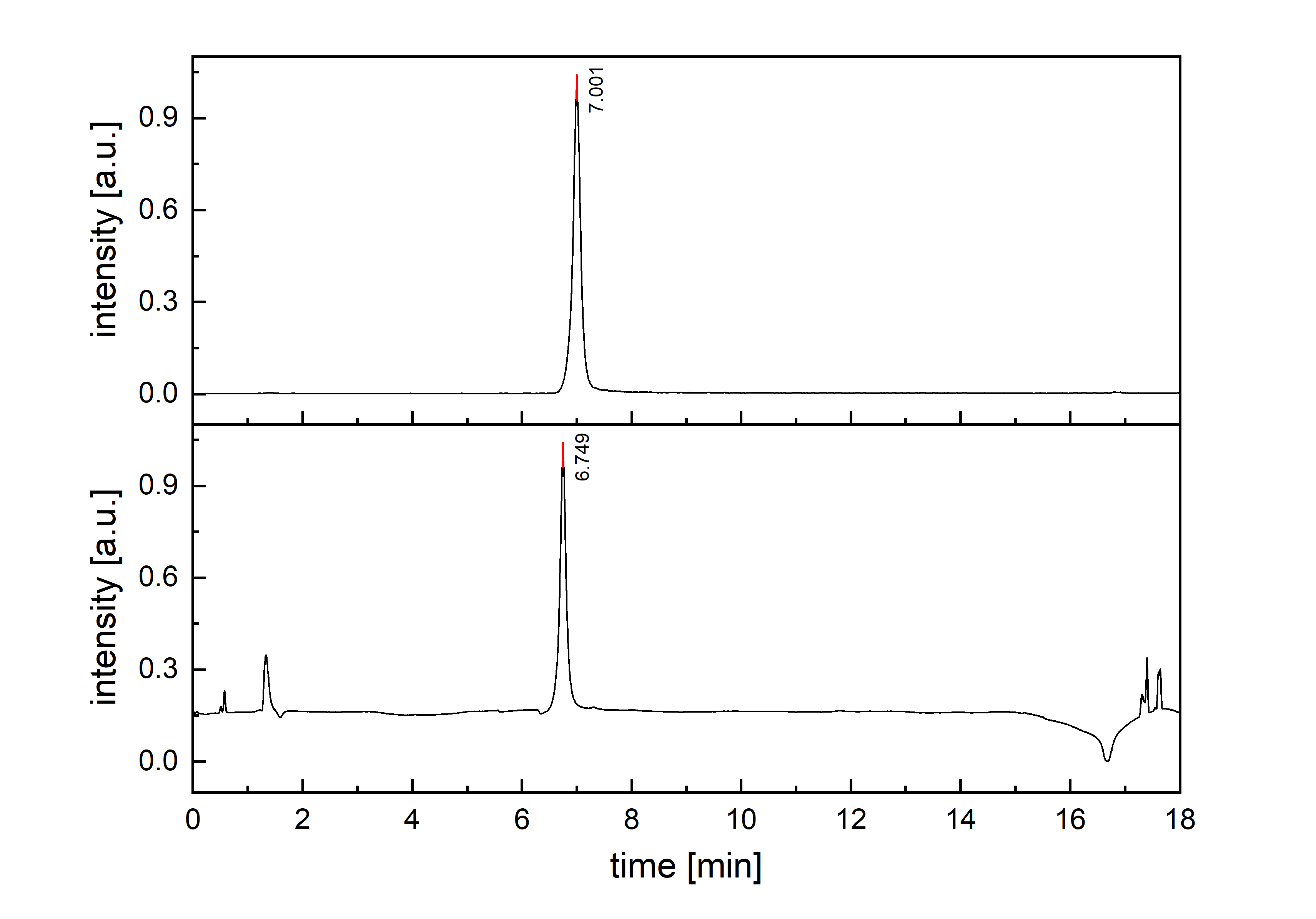


Fig. S66: Analytical HPLC-chromatograms (System C) of coninjected [^18^F]F-2 and its ^19^F-labelled analogue 2. Top trace: γ-channel, bottom trace: UV-channel.


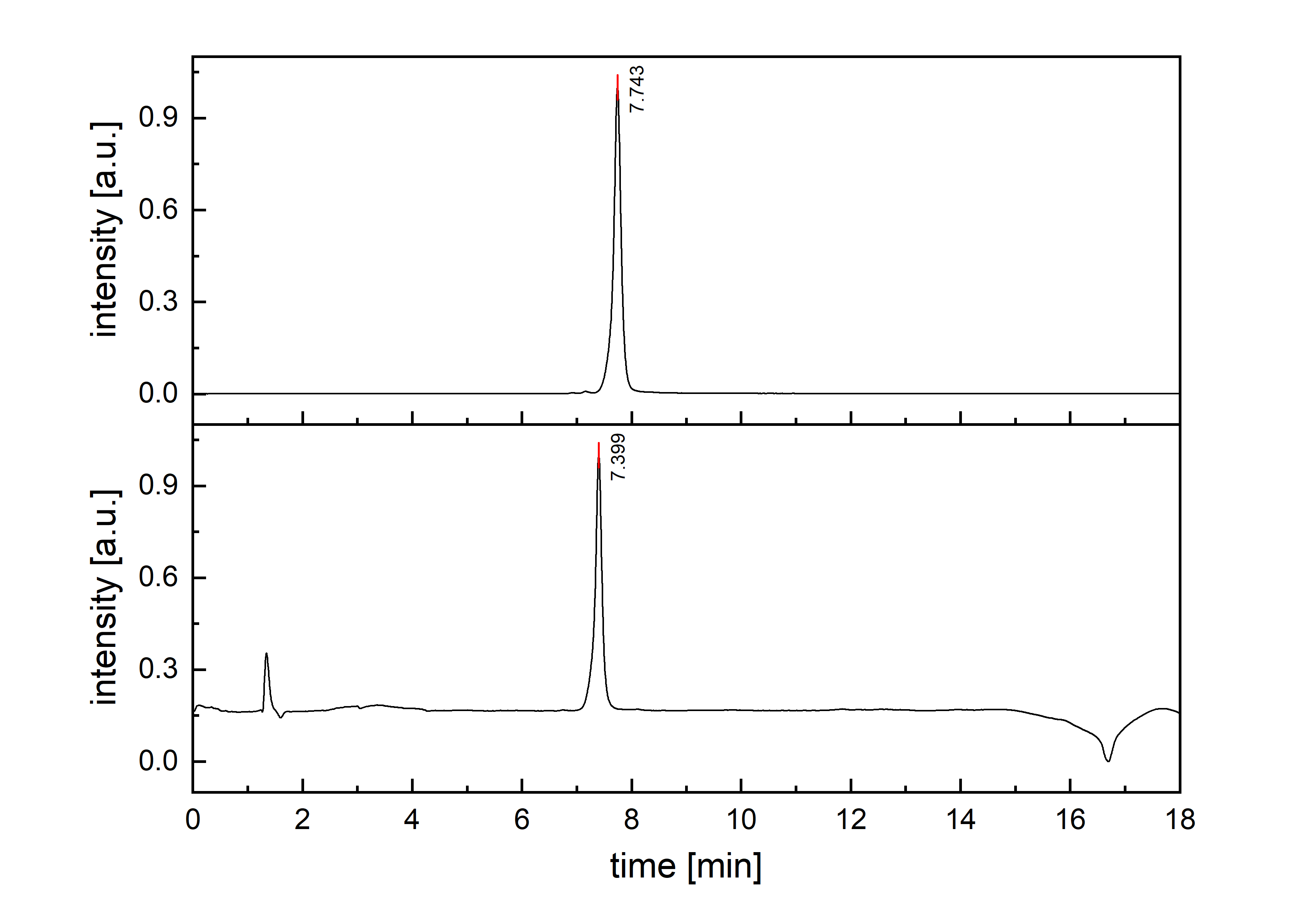


Fig. S67: Analytical HPLC-chromatograms (System C) of coninjected [^18^F]F-3 and its ^19^F-labelled analogue 3. Top trace: γ-channel, bottom trace: UV-channel.


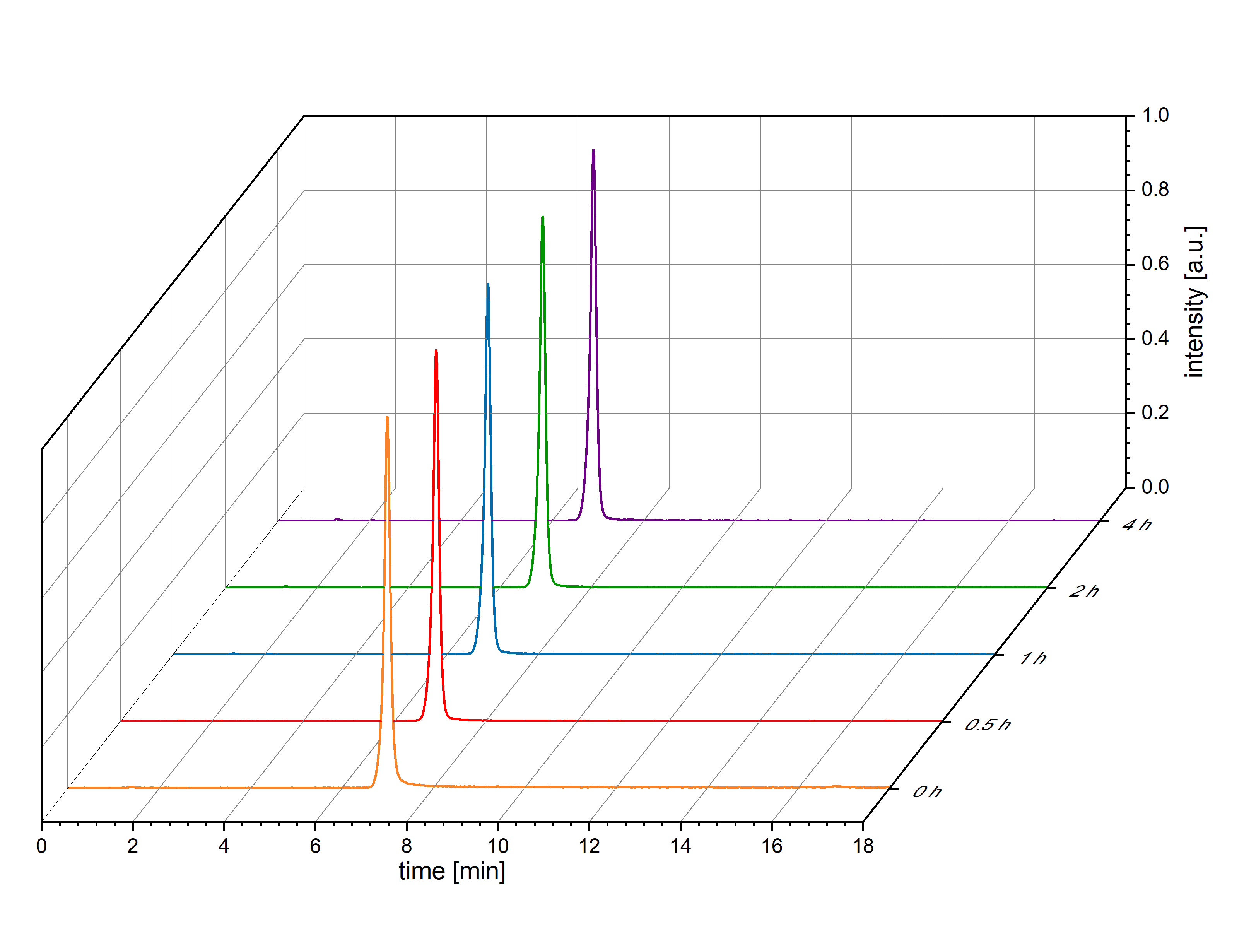


Fig. S68: Radio-HPLC chromatograms of [^18^F]F-2 after incubation in human serum at 37 °C and subsequent protein precipitation at 0 h (orange), 0.5 h (red), 1 h (blue), 2 h (green) and 4 h (purple).


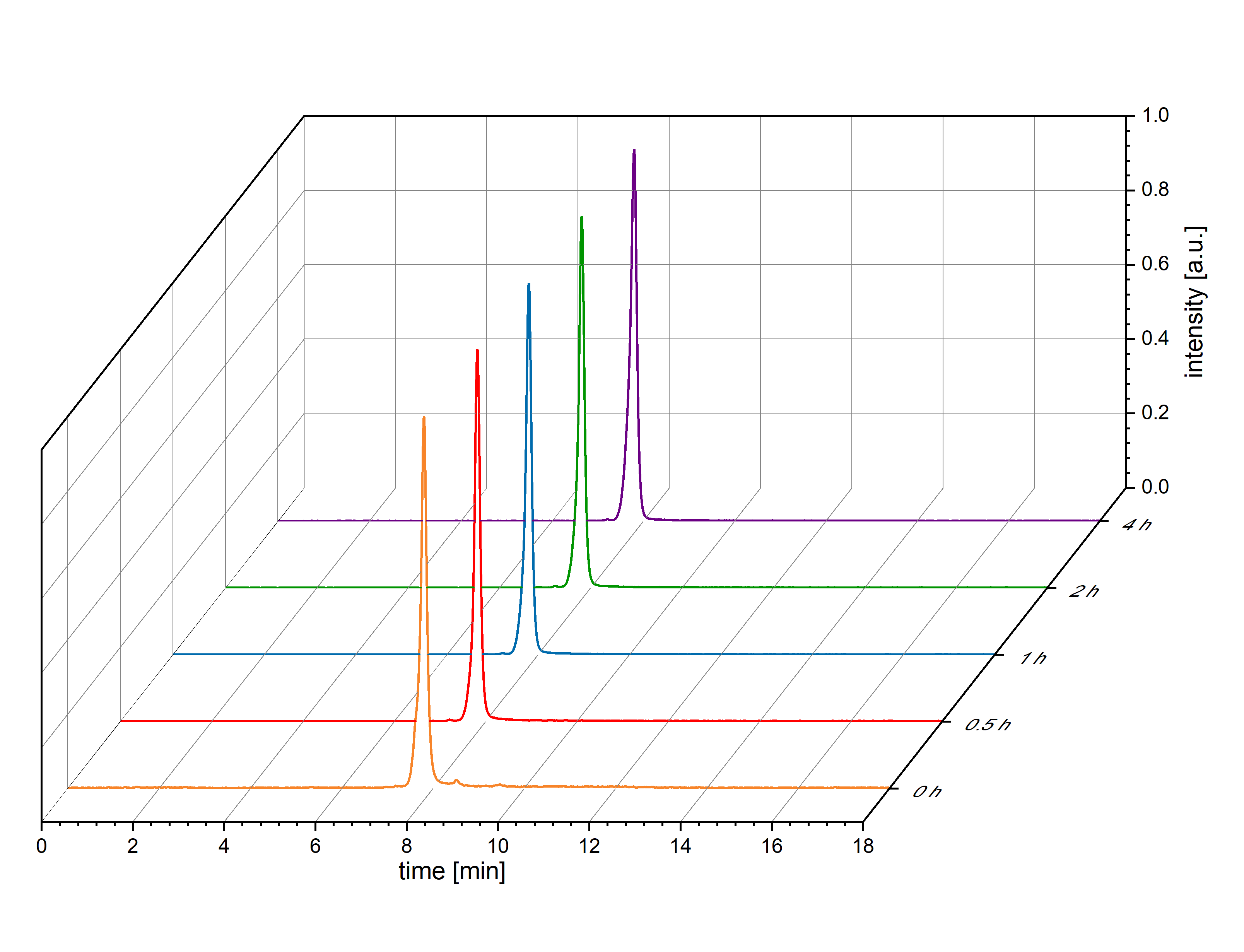


Fig. S69: Radio-HPLC chromatograms of [^18^F]F-3 after incubation in human serum at 37 °C and subsequent protein precipitation at 0 h (orange), 0.5 h (red), 1 h (blue), 2 h (green) and 4 h (purple).


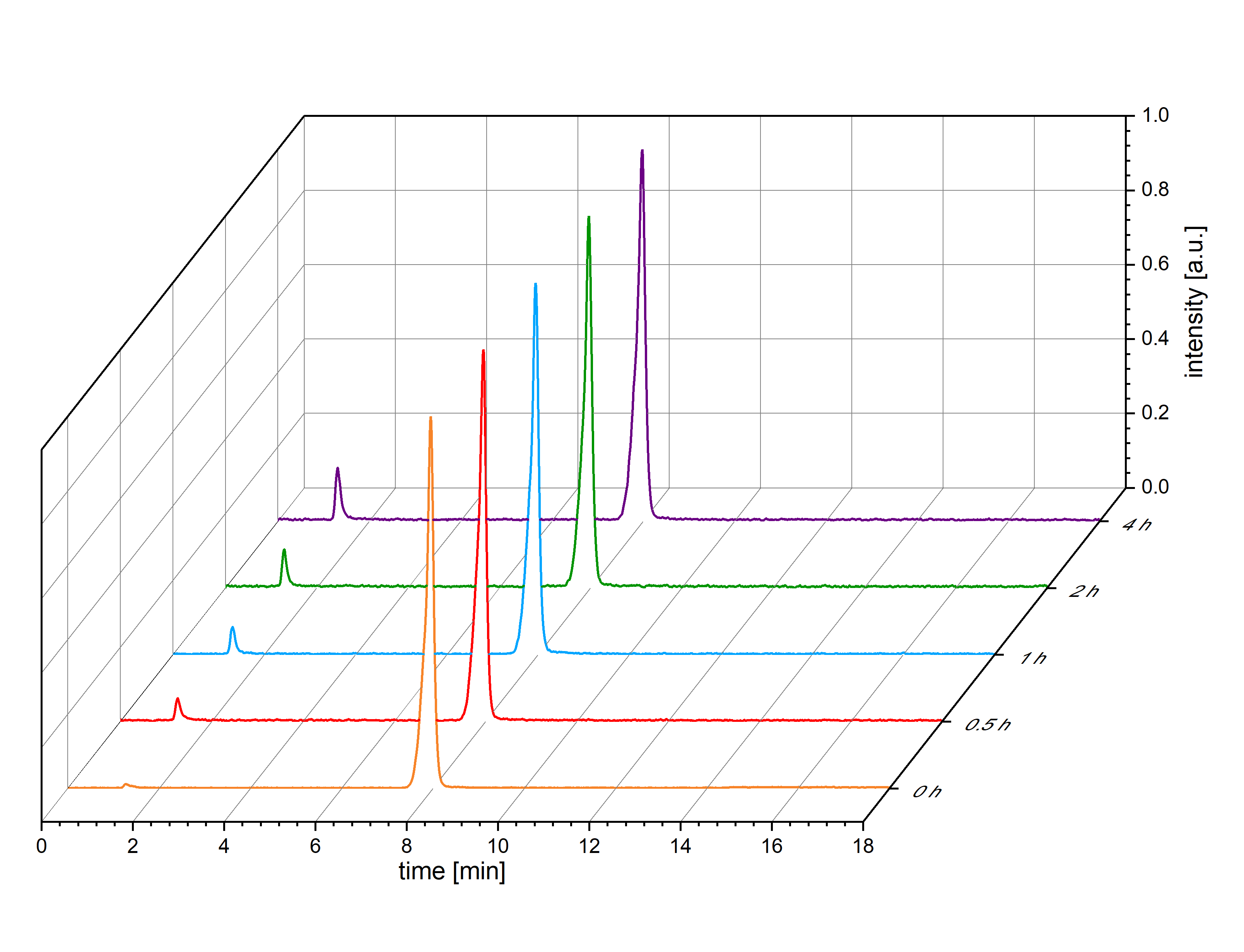


Fig. S70: Radio-HPLC chromatograms of Al[^18^F]F-36 after incubation in human serum at 37 °C and subsequent protein precipitation at 0 h (orange), 0.5 h (red), 1 h (blue), 2 h (green) and 4 h (purple).


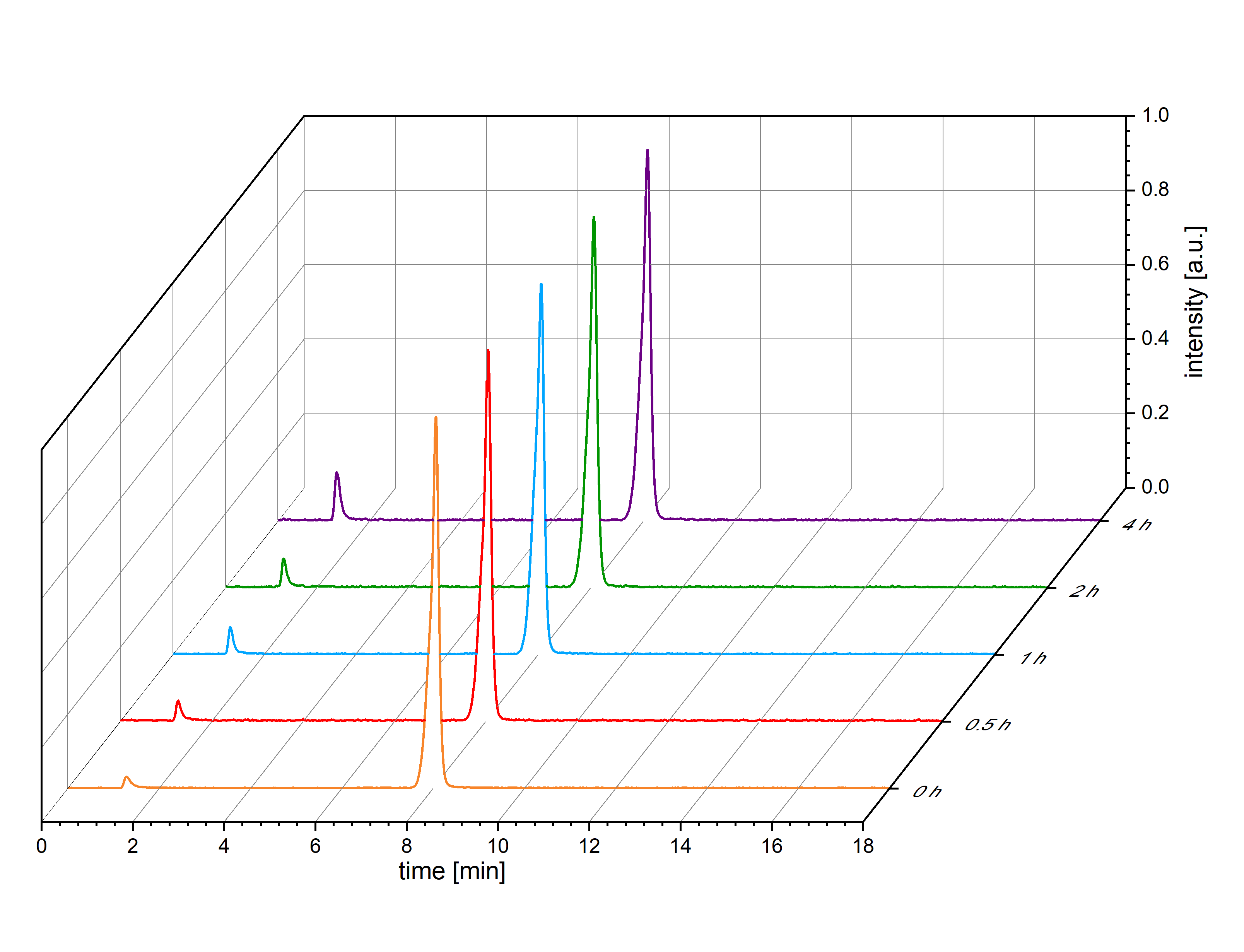


Fig. S71: Radio-HPLC chromatograms of Al[^18^F]F-37 after incubation in human serum at 37 °C and subsequent protein precipitation at 0 h (orange), 0.5 h (red), 1 h (blue), 2 h (green) and 4 h (purple).

# Biology

**Real-time radioligand binding**

*Methodology*

The LigandTracer system (Ridgeview Instruments AB, Uppsala, Sweden) allows assessing the binding kinetics (association rate constant *k*_a_ and dissociation rate constant *k*_d_) and therefore dissociation constant (*K*_D_) of radiolabeled compounds. For that purpose, target-positive PC3 PD-L1 cells are seeded to a quadrant of a petri dish (Thermo Scientific Nunclon Delta #150350). In our initial tests, 1.2x10^6^ cells were seeded one day prior to the experiment.

The petri dish is then placed in the instrument, the culture medium removed and replaced with 3 mL CO2-independent medium (Gibco #18045088, ThermoFisher Germany). In the LigandTracer system, the dish rotates on an inclined base, with cells in medium (3 mL) located on one side (target), while no cells (background) are located on the other side. Radioactivity (counts per second) is then constantly measured for 30 seconds on either side. This allows continuous measurements of two (or more) alternating parts of the dish for bound radioactivity, with target – background indicating binding over time.

All experiments were performed at room temperature. Association of compounds was observed following incubation with two increasing concentrations of the tracer. The tracer-containing solution was then exchanged for fresh medium, to allow observation of dissociation for at least 120 min.

*Binding of [^18^F]F-****3*** *and of Al[^18^F]F-****36*** *to PC3 cells, overexpressing PD-L1*

Similar to our previously reported compounds, we attempted to determine kinetic parameters of the herein reported candidates using real-time radioligand binding. However, initial findings yielded no or only a low signal increase over time, complicating accurate fitting of the trace (Suppl. Fig 72). This is demonstrated by [^18^F]F-**3** in Figure 72A. Traces there show bound radioactivity over time [derived from radioactivity measured in the target region (PC3 PD-L1 cells) of the petri dish with radioactivity in the background region subtracted], indicating that almost no binding takes place. Al[^18^F]F-**36** (Figure 72B) fares marginally better, with some signal increase over time. However, the dissociation rate constant is determined to be much slower than all of our previously reported PD-L1 small-molecule tracers. This led us to the conclusion that the binding affinities of these candidates must be rather low and that a competition binding assay with our previously reported compound [^64^Cu]Cu-**1** would be more suitable.


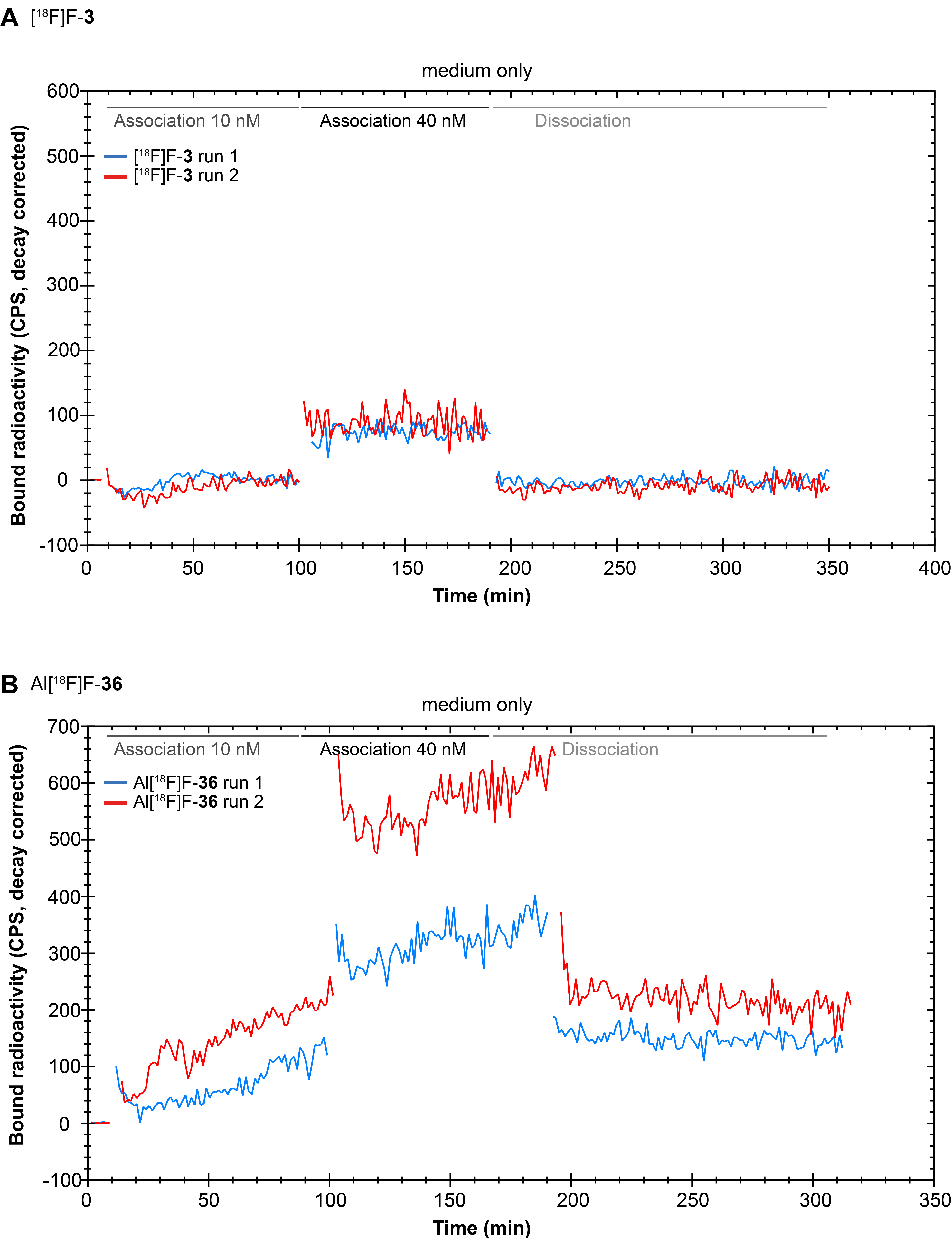


Fig. S72: Example data from real-time radioligand binding data of compounds [^18^F]F-3 and Al[^18^F]F-36. Traces show no ([^18^F]F-3) and very little (Al[^18^F]F-36) signal increase over time, interfering with reliable fitting of the curve.
